# Battle of the artificial intelligence: a comprehensive comparative analysis of DeepSeek and ChatGPT for urinary incontinence-related questions

**DOI:** 10.3389/fpubh.2025.1605908

**Published:** 2025-07-23

**Authors:** Huawei Cao, Changzhen Hao, Tao Zhang, Xiang Zheng, Zihao Gao, Jiyue Wu, Lijian Gan, Yu Liu, Xiangjun Zeng, Wei Wang

**Affiliations:** ^1^Department of Urology, Beijing Chao-yang Hospital, Capital Medical University, Beijing, China; ^2^Department of Urology, Peking University International Hospital, Beijing, China; ^3^Department of Pathology, School of Basic Medical Sciences, Capital Medical University, Beijing, China; ^4^Urinary and Nephropathy Center, Beijing Chao-yang Hospital, Capital Medical University, Beijing, China; ^5^Department of Physiology and Pathophysiology, Capital Medical University, Beijing, China

**Keywords:** artificial intelligence, urinary incontinence, ChatGPT, DeepSeek, comparative analysis

## Abstract

**Background:**

With the rapid advancement and widespread adoption of artificial intelligence (AI), patients increasingly turn to AI for initial medical guidance. Therefore, a comprehensive evaluation of AI-generated responses is warranted. This study aimed to compare the performance of DeepSeek and ChatGPT in answering urinary incontinence-related questions and to delineate their respective strengths and limitations.

**Methods:**

Based on the American Urological Association/Society of Urodynamics, Female Pelvic Medicine & Urogenital Reconstruction (AUA/SUFU) and European Association of Urology (EAU) guidelines, we designed 25 urinary incontinence-related questions. Responses from DeepSeek and ChatGPT-4.0 were evaluated for reliability, quality, and readability. Fleiss' kappa was employed to calculate inter-rater reliability. For clinical case scenarios, we additionally assessed the appropriateness of responses. A comprehensive comparative analysis was performed.

**Results:**

The modified DISCERN (mDISCERN) scores for DeepSeek and ChatGPT-4.0 were 28.24 ± 0.88 and 28.76 ± 1.56, respectively, showing no practically meaningful difference [*P* = 0.188, Cohen's *d* = 0.41 (95% *CI*: −0.15, 0.97)]. Both AI chatbots rarely provided source references. In terms of quality, DeepSeek achieved a higher mean Global Quality Scale (GQS) score than ChatGPT-4.0 (4.76 ± 0.52 vs. 4.32 ± 0.69, *P* = 0.001). DeepSeek also demonstrated superior readability, as indicated by a higher Flesch Reading Ease (FRE) score (76.43 ± 10.90 vs. 70.95 ± 11.16, *P* = 0.039) and a lower Simple Measure of Gobbledygook (SMOG) index (12.26 ± 1.39 vs. 14.21 ± 1.88, *P* < 0.001), suggesting easier comprehension. Regarding guideline adherence, DeepSeek had 11 (73.33%) fully compliant responses, while ChatGPT-4.0 had 13 (86.67%), with no significant difference [*P* = 0.651, Cohen's *w* = 0.083 (95% CI: 0.021, 0.232)].

**Conclusion:**

DeepSeek and ChatGPT-4.0 might exhibit comparable reliability in answering urinary incontinence-related questions, though both lacked sufficient references. However, DeepSeek outperformed ChatGPT-4.0 in response quality and readability. While both AI chatbots largely adhered to clinical guidelines, occasional deviations were observed. Further refinements are necessary before the widespread clinical implementation of AI chatbots in urology.

## 1 Introduction

Urinary incontinence (UI) is a prevalent global health issue that significantly impairs quality of life, particularly among female patients (1). Defined by the International Continence Society as “*the involuntary loss of urine”* (2), UI not only affects physical health but also leads to profound psychological and social dysfunction (3). Many patients with UI hesitate to seek professional medical help due to stigma, lack of disease awareness, and concerns about treatment efficacy (4–6), exacerbating health burdens and reducing quality of life. Thus, providing accurate, detailed, and timely UI-related information is critical for both patients and healthcare providers (7).

However, access to high-quality medical information remains inadequate, especially in urology. Recently, artificial intelligence (AI) has gained traction in healthcare, particularly for delivering medical information and aiding decision-making (8). Public interest in AI-driven health consultations is also rapidly growing (9). ChatGPT, a large language model (LLM) developed by OpenAI, has demonstrated robust natural language processing capabilities across diverse fields (10). Yet, its reliability in providing accurate medical information remains debated (11, 12). DeepSeek, an emerging open-source LLM (13), has not been thoroughly evaluated for medical applications, especially in surgical subspecialties like urology (14).

This study aimed to compare the performance of DeepSeek and ChatGPT in answering UI-related questions and to delineate their respective strengths and limitations. The findings can serve as a valuable reference for enhancing medical AI systems and assist the public in making informed healthcare decisions.

## 2 Materials and methods

### 2.1 Study design

This cross-sectional comparative study was designed to systematically evaluate and compare the performance of two popular AI chatbots [ChatGPT-4.0 (OpenAI) and DeepSeek (DeepSeek AI)] in providing medical information related to UI. The evaluation framework was established based on the clinical guidelines issued by the American Urological Association/Society of Urodynamics, Female Pelvic Medicine & Urogenital Reconstruction (AUA/SUFU) and the European Association of Urology (EAU) (15–17).

### 2.2 Questionnaire development

A comprehensive questionnaire comprising 25 UI-related questions was meticulously developed by a panel of urology specialists based on the AUA/SUFU and EAU guidelines (15–17). The questionnaire was strategically structured into two distinct sections:

(1) Fundamental conceptual questions (*n* = 10): encompassing core aspects of UI including definition, classification systems, etiological factors, diagnostic criteria, and therapeutic approaches.
(2) Clinical scenario-based questions (*n* = 15):

- Diagnostic evaluation (4 cases).
- Treatment algorithm selection (8 cases).
- Postoperative complication management (3 cases)

**Figure 1:**
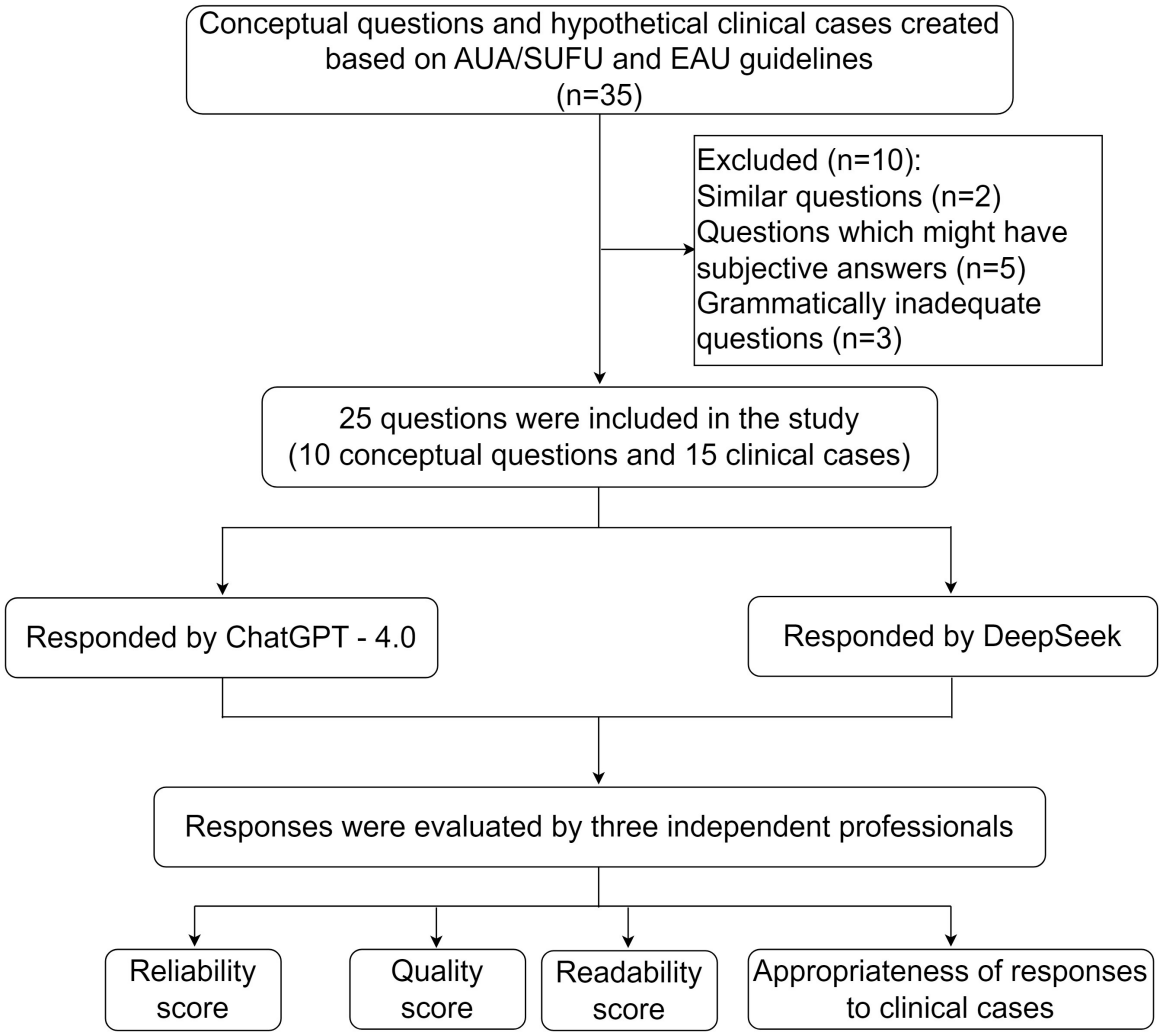
Flow chart of question inclusion and response evaluation. AUA/SUFU, American Urological Association/Society of Urodynamics, Female Pelvic Medicine & Urogenital Reconstruction; EAU, European Association of Urology.

Each question was explicitly mapped to specific guideline recommendations to ensure content validity and minimize potential evaluation bias (Figure 1, Supplementary Table 1).

### 2.3 AI chatbot platforms

This study evaluated two cutting-edge LLMs:

(1) ChatGPT-4.0 (OpenAI^®^ release date: March 14, 2023): this commercially available LLM represents significant advancements in natural language processing capabilities compared to its predecessor (ChatGPT-3.5), particularly in contextual understanding and response generation.
(2) DeepSeek AI system (version accessible January 5, 2024): developed by DeepSeek AI (Hangzhou, China), this open-access platform features two specialized models:

- DeepSeek-V3 excels in multilingual processing and long-text generation, making it suitable for large-scale natural language processing tasks such as conversational AI, multilingual translation, and content generation.
- DeepSeek-R1 performs well in complex mathematical problems and logical reasoning tasks, making it suitable for tasks requiring in-depth reasoning.

### 2.4 Evaluation methodology

ChatGPT-4.0 provided responses to all 25 questions, while DeepSeek-V3 answered questions 1–10 and DeepSeek-R1 answered questions 11–25. The reliability, quality, readability, and appropriateness of the chatbot responses were independently evaluated by three board-certified urologists with at least 10 years of experience, based on the AUA/SUFU and EAU guidelines and clinical practice. The final score for each evaluation item was determined by averaging the scores from the three experts (Figure 1). To assess the inter-rater reliability of subjective scoring items, including those related to reliability and quality metrics, we utilized Fleiss' kappa statistic. The evaluators were independent of the questionnaire designers and question-askers.

The modified DISCERN (mDISCERN) tool was used to assess the reliability of the responses. The DISCERN tool has been used in previous studies to evaluate the reliability of online health information and consists of three parts (18). The first part is primarily used to assess the reliability of the provided information, while the second and third parts are designed to evaluate the overall quality of information related to disease treatment options. In this study, our questions included both conceptual questions and clinical case management questions. Therefore, the mDISCERN tool included only the first part of the original tool (Supplementary Table 2). For each content in the mDISCERN tool, the scoring method was as follows: 1 point for no response, 2–4 points for a partial response, and 5 points for a complete response. The total mDISCERN score was categorized as follows: poor (8–15 points), fair (16–31 points), and good (32–40 points).

The Global Quality Scale (GQS) tool was utilized to evaluate the quality of the responses. Previous studies have used the GQS tool to evaluate the quality of ChatGPT responses (19). The scoring range was 1–5, with higher scores indicating higher quality (Supplementary Table 3). Additionally, the scale was used for quality grading: 1–2 points represented low quality, 3 points represented moderate quality, and 4–5 points represented high quality.

For readability assessment, the widely used Flesch Reading Ease (FRE) (20) and Simple Measure of Gobbledygook (SMOG) tools (21) were employed to evaluate the readability of ChatGPT 4.0 and DeepSeek (Supplementary Table 4). The FRE score ranges from 0 to 100; higher scores indicate easier readability. The SMOG tool was used to calculate the years of education required to understand a given text. As the SMOG-based readability score increases, the FRE score decreases. For texts intended for general readers, the acceptable FRE score should be >60, while the SMOG score should be below 12.

For clinical scenarios, we further evaluated the adherence of ChatGPT-4.0 and DeepSeek responses to the guidelines. If a particular response explicitly contradicted any part of the guidelines, it was considered a violation of the guidelines; if a response did not contradict the guidelines but included content not covered by them, it was deemed partially compliant with the guidelines; if the entire response adhered to the content of the guidelines, it was considered fully compliant. Additional evaluations for clinical scenarios included whether the response recommended consulting a healthcare professional, whether it expressed encouragement or comfort, and whether it included a disclaimer stating that it could not provide medical advice.

### 2.5 Ethical approval

The AI chatbot platforms used in this study are publicly available tools, and this study did not involve human or animal participants. Therefore, the ethical committee approval was not required.

### 2.6 Statistical analysis

Statistical analysis was performed using SPSS 26.0 software (IBM SPSS Statistics for Windows). The Kolmogorov-Smirnov test was conducted to assess the normality and equal variance of continuous variables. Normally distributed continuous variables were expressed as mean ± standard deviation, while non-normally distributed continuous variables were expressed as median (interquartile range). Categorical variables were expressed as *n* (%). To further compare the performance of ChatGPT-4.0 and DeepSeek, paired *t*-tests or Wilcoxon signed-rank tests were used for continuous variables, and Pearson's chi-square tests or Fisher's exact tests were used for categorical variables. All statistical tests were two-tailed, with *P* < 0.05 considered statistically significant. Cohen's *d* and Cohen's *w* were statistical measures used to evaluate the effect size for continuous and categorical variables, respectively.

## 3 Results

### 3.1 Response characteristics and qualitative evaluations

Both DeepSeek and ChatGPT-4.0 provided responses to all 25 UI-related questions (Supplementary Table 1). For questions 1–10, both ChatGPT-4.0 and DeepSeek-V3 provided direct formal responses. However, for questions 11–25, compared to ChatGPT-4.0, DeepSeek-R1 first presented its analytical reasoning process before providing formal responses to the questions. DeepSeek has significantly more words for formal responses than ChatGPT 4.0 [530.00 (448.00, 582.00) vs. 374.00 (275.50, 411.00), *P* < 0.001] (Table 1). Additionally, the average word count of DeepSeek-R1's analytical reasoning content was 444.07 ± 93.52 in responses for the 15 clinical scenarios.

**Table 1:** Comparisons of the reliability, quality, and readability of ChatGPT-4.0 and DeepSeek responses.

**Items**	**ChatGPT-4.0 (*N* = 25)**	**DeepSeek (*N* = 25)**	***P*-value**	**Cohen's *d* (95%*CI*)**
**Reliability**				
mDISCERN score (Mean ± SD)	28.24 ± 0.88	28.76 ± 1.56	0.188	0.41 (−0.15, 0.97)
**Quality**				
GQS score (Mean ± SD)	4.32 ± 0.69	4.76 ± 0.52	0.001	0.72 (0.15, 1.29)
**Readability**				
Word count [median (IQR)]	374.00 (275.50, 411.00)	530.00 (448.00, 582.00)	<0.001	
FRE (Mean ± SD)	70.95 ± 11.16	76.43 ± 10.90	0.039	0.58 (0.07, 1.09)
SMOG (Mean ± SD)	14.21 ± 1.88	12.26 ± 1.39	<0.001	−1.18 (−1.78, −0.58)

mDISCERN, modified DISCERN; GQS, global quality score; FRE, Flesch reading ease score; SMOG, simple measure of Gobbledygook; SD, standard deviation; IQR, interquartile range; 95%CI, 95% confidence interval.

The qualitative evaluations of ChatGPT-4.0 and DeepSeek's responses are presented in Table 2. The evaluators conducted a comprehensive comparison of the overall performance of the two AI chatbots, focusing on aspects such as presentation of the responses, lucidity, accuracy, reading difficulty, and relevance to the questions.

**Table 2:** Qualitative comparisons of the two AI chatbots' responses by three evaluators.

**Features**	**ChatGPT-4.0**	**DeepSeek**
Presentation of the responses	Responses were well-structured and highlighted the key points.	Responses demonstrated a clear structure, comprehensive and specific content, and a high degree of feasibility.
Lucidity	Expression was lucid.	Lucid.
Accuracy	Accuracy was observed but some answers lacked essential details; all responses lacked references.	Accurate; most responses lacked references.
Reading difficulty	Most responses were easy to read, but there were a few complex technical terms.	Easy to read.
Relevance to the questions	Relevant	Relevant

### 3.2 Reliability and quality

After reaching consensus, the mDISCERN scores for ChatGPT-4.0 and DeepSeek were 28.24 ± 0.88 and 28.76 ± 1.56, respectively, showing no practically meaningful difference [*P* = 0.188, Cohen's *d* = 0.41 (95% *CI*: −0.15, 0.97)] (Table 1). In the reliability assessment of ChatGPT-4.0 and DeepSeek, the three evaluators achieved a high degree of inter-rater consistency [κ = 0.773 (95% *CI*: 0.614, 0.883) and 0.863 (95% *CI*: 0.755, 0.932), respectively] (Supplementary Table 5).

The distribution of mDISCERN scores for responses from both platforms is shown in Table 3. All responses from ChatGPT-4.0 demonstrated fair reliability [25 (100%)]. Most responses from DeepSeek also showed fair reliability [22 (88.0%)], with a minority demonstrating good reliability [3 (12.0%)]. The distribution of mDISCERN scores between the two AI chatbots showed no significant difference (*P* = 0.235). The limited number of responses achieving good reliability scores for both AI chatbots was primarily due to their infrequent provision of information sources (Supplementary Table 1). ChatGPT-4.0 did not provide any references or guidelines to support its responses, while DeepSeek cited relevant guidelines for questions 11, 12, and 18.

**Table 3:** Comparison of the distribution of scores between the two AI chatbots according to the mDISCERN, GQS, FRE, and SMOG scale.

**Criteria**	ChatGPT-4.0 (*N* = 25)^*^	DeepSeek (*N* = 25)^*^	***P*-value**
**mDISCERN criteria**			
Poor (score <16)	0 (0%)	0 (0%)	0.235
Fair (16 ≤ score ≤ 31)	25 (100%)	22 (88.0%)	
Good (score > 31)	0 (0%)	3 (12.0%)	
**GQS criteria**			
Low quality (1–2)	0 (0%)	0 (0%)	0.609
Moderate quality (3)	3 (12.0%)	1 (4.0)	
High quality (4–5)	22 (88.0%)	24 (96.0%)	
**FRE criteria**			
<11 years old (90–100)	1 (4.0%)	2 (8.0%)	0.356
11–12 years old (80–89)	4 (16.0%)	8 (32.0%)	
13–15 years old (70–79)	7 (28.0%)	9 (36.0%)	
High school level (60–69)	10 (40.0%)	4 (16.0%)	
College level (50–59)	2 (8.0%)	2 (8.0%)	
College graduate (30–49)	1 (4.0%)	0 (0%)	
Expert level (0–29)	0 (0%)	0 (0%)	
**SMOG criteria**			
Primary school (1–6)	0 (0%)	0 (0%)	0.002
Junior high school (7–8)	0 (0%)	0 (0%)	
High school (9–12)	3 (12.0%)	14 (56.0%)	
Undergraduate college (13–16)	19 (76.0%)	11 (44.0%)	
Graduate or higher (≥17)	3 (12.0%)	0 (0%)	

^*^n (%).

mDISCERN, modified DISCERN; GQS, global quality score; FRE, Flesch reading ease score; SMOG, simple measure of Gobbledygook.

Regarding quality assessment, DeepSeek's average GQS score was significantly higher than that of ChatGPT-4.0 (4.76 ± 0.52 vs. 4.32 ± 0.69, *P* = 0.001) (Table 1). In the quality assessment of ChatGPT-4.0 and DeepSeek, the inter-rater reliability among the three evaluators also showed substantial agreement [κ = 0.726 (95% *CI*: 0.545, 0.856) and 0.759 (95% *CI*: 0.594, 0.876), respectively] (Supplementary Table 6).

DeepSeek's responses were more specific and practical, thus potentially more helpful to patients (Supplementary Table 1). For example, in response to question 12, ChatGPT-4.0 only provided an overview of bladder diary's purpose, content, and recording methods, while DeepSeek-R1 not only covered these aspects but also included a bladder diary template for user reference. However, the distribution of GQS scores between the two AI chatbots showed no significant difference (*P* = 0.609) (Table 3). Most of the ChatGPT-4.0's responses were of high quality [22 (88.0%)], followed by moderate quality [3 (12.0%)]. For DeepSeek, all but one response was of high quality [24 (96.0%)].

### 3.3 Readability

DeepSeek's responses had significantly higher average FRE scores than ChatGPT-4.0 (76.43 ± 10.90 vs. 70.95 ± 11.16, *P* = 0.039) (Table 1). Correspondingly, DeepSeek's responses had significantly lower average SMOG scores than ChatGPT-4.0 (12.26 ± 1.39 vs. 14.21 ± 1.88, *P* < 0.001) (Table 1). Therefore, although DeepSeek's responses were longer, they were easier to read.

When assessed by the FRE scale, the reading difficulty of ChatGPT-4.0's responses was primarily at the “High school” level [10 (40.0%)] and “13–15 years old” level [7 (28.0%)], while DeepSeek's responses were mainly at the “13–15 years old” level [9 (36.0%)] and “11–12 years old” level [8 (32.0%)]. The distribution of FRE scores showed no significant difference between the two AI chatbots (*P* = 0.356) (Table 3). When assessed by the SMOG scale, the reading difficulty of ChatGPT-4.0's responses was primarily at the “Undergraduate college” level [19 (76.0%)], while DeepSeek's responses were mainly at the “High school” level [14 (56.0%)] (Table 3). Based on SMOG score distribution, ChatGPT-4.0's responses were generally more difficult to read than DeepSeek's.

### 3.4 Appropriateness of responses for clinical scenarios

**Figure 2:**
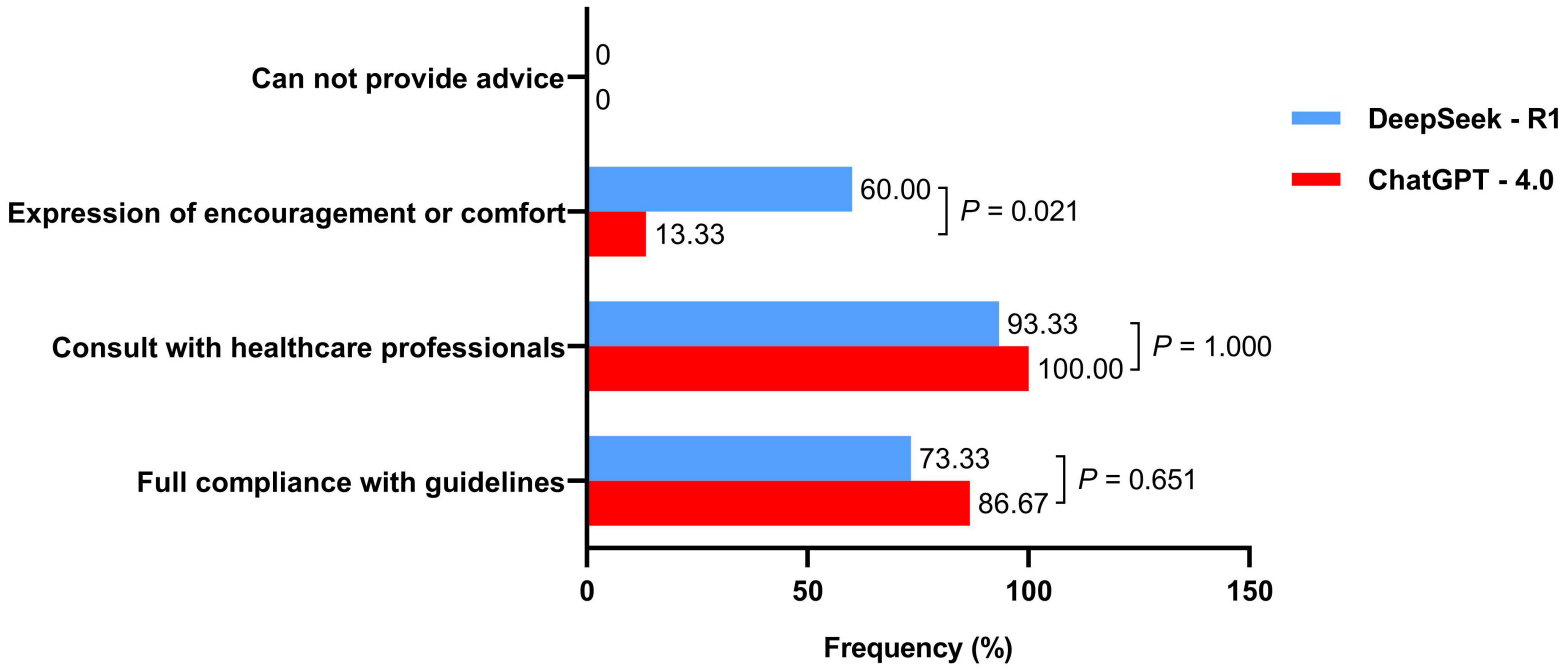
Evaluation of the appropriateness of responses generated by two artificial intelligence chatbots in clinical scenarios.

The appropriateness results of the responses to the 15 clinical case questions are shown in Figure 2. DeepSeek-R1 demonstrated 73.33% (11/15) full guideline compliance, while ChatGPT-4.0 showed 86.67% (13/15) full compliance, with no significant difference in adherence rates [*P* = 0.651, Cohen's *w* = 0.083 (95% *CI*: 0.021, 0.232)] and no guideline violations observed in either group.

Both DeepSeek-R1 and ChatGPT-4.0 frequently emphasized the importance of consulting healthcare professionals [14 (93.33%) vs. 15 (100%), *P* = 1.000]. Regarding medical humanistic care, DeepSeek-R1 more frequently expressed encouragement or comfort compared to ChatGPT-4.0 [9 (60.00%) vs. 2 (13.33%), *P* = 0.021]. For example, when asked to provide guidance on pelvic floor muscle training, DeepSeek-R1 affirmed the effectiveness of this treatment approach and added “You've got this!” after giving professional instructions (Question 16 in Supplementary Table 1). Neither AI chatbot included disclaimers about being unable to provide medical advice.

## 4 Discussion

In this study, two popular AI chatbots, DeepSeek and ChatGPT, were evaluated and compared for their responses to UI-related questions. Our findings demonstrate comparable reliability between both platforms, while revealing significant advantages of DeepSeek in delivering higher-quality and more comprehensible responses. Notably, both AI chatbots exhibited strong adherence to clinical guidelines when responding to case-based questions, with DeepSeek demonstrating superior capabilities in providing encouraging communication.

The rapid advancement of AI in healthcare has led to increasing patient reliance on AI platforms for medical information, paralleled by growing institutional adoption of AI-assisted clinical decision support systems. In pathology diagnostics, AI has demonstrated remarkable progress in cancer screening, lesion localization, and diagnostic efficiency enhancement (22, 23). If an AI chatbot is capable of providing precise and comprehensible responses to healthcare-related inquiries, it could enhance patients' medical knowledge, facilitate early diagnosis and treatment of diseases, and improve the efficiency of doctor-patient communication. Consequently, the exploration of methods to enhance the feasibility of AI chatbots in healthcare has become a topic of significant interest for scholars (24). As far as we know, studies comparing the performance of DeepSeek and ChatGPT in answering healthcare-related questions are scarce. Furthermore, there is no research comparing the performance of these two AI chatbots in answering questions related to urological cases.

DeepSeek and ChatGPT-4.0 might have comparable reliability in answering UI-related questions. However, the limited number of questions (*N* = 25) constrained the ability to draw a definitive conclusion. Both AI chatbots predominantly achieved fair reliability ratings, with only a minority of DeepSeek-R1 responses reaching good reliability. This limitation primarily stems from inadequate source documentation, which is consistent with previous research. McMahon et al. (25) reported that, in their comparison of ChatGPT and Bing AI regarding the responses to questions about kidney stones, ChatGPT did not provide references, whereas Bing AI consistently included references in its responses. Zhou et al. (26) further indicated that when generating educational materials for individuals undergoing spinal surgery, both ChatGPT and DeepSeek lacked explicit citations or supporting evidence in their outputs. These findings suggest the need for dedicated “medical professional modes” that enforce rigorous source citation to enhance response reliability.

Furthermore, DeepSeek-R1 presented the analytical reasoning process prior to offering a formal response. The content of analytical reasoning encompassed not only the response to a specific diagnostic or treatment question but also the validation of the user's issue and a comprehensive overview of UI. This segment enabled users to ascertain whether DeepSeek-R1 had accurately comprehended their questions. In addition, the analytical reasoning process might enhance users' understanding of the basis for the responses. However, additional relevant studies are required to further validate this hypothesis.

Regarding response quality, both AI chatbots generally provided accurate and comprehensive medical advice. Additionally, the responses of both AI chatbots demonstrated a clear and well-organized layout structure. However, there exist differences in their responses. The responses of ChatGPT-4.0 are frequently concise and straightforward, but lacking in details, which is analogous to other research findings (27). Conversely, the responses of DeepSeek are highly detailed, expanding on each subsection of the content. When giving diagnostic or treatment recommendations, DeepSeek provided the most specific plans possible, which enhanced the feasibility of its responses.

Readability constitutes another concern regarding the medical information generated by search engines or AI chatbots. In this study, the reading difficulty of the responses from DeepSeek was lower than that of ChatGPT-4.0. In another study, the reading difficulty of the content generated by DeepSeek-R1 and ChatGPT-4.0 was both at the “high school” level; however, the average reading difficulty of the content generated by DeepSeek-R1 was lower than that of ChatGPT-4.0 (26). Furthermore, Fahy et al. (28) concluded that, according to the SMOG index, the average reading difficulty of the information related to anterior cruciate ligament injury generated by ChatGPT-4.0 fell between the “University junior” and “University senior” levels. We hypothesize that this readability superiority of DeepSeek might be ascribed to the fact that DeepSeek regards users as patients lacking medical knowledge. The analytical reasoning section of DeepSeek-R1's responses can serve as evidence. Nevertheless, the precise cause demands further investigation. To further improve the readability of responses from AI chatbots, two question-asking interfaces for patients and medical personnel can be established for selection. In this manner, the AI chatbot can select the corresponding level of word difficulty and sentence complexity based on the user's circumstances.

Neither of the two chatbots' responses violated the guidelines, indicating that both AI chatbots have great potential to provide accurate information related to UI for patients or doctors. However, we found that DeepSeek and ChatGPT-4.0 occasionally offered opinions or suggestions not covered by the current guidelines. As we know, both LLMs are trained based on a large amount of data. However, the data mainly comes from the internet and has not undergone strict screening (29), which might result in the emergence of suggestions not covered by the guidelines.

Sandmann et al. (14) conducted an evaluation of DeepSeek, GPT-4o, and Gemini-2.0 Flash Thinking Experiment. They also identified the phenomenon of “artificial hallucination” in the responses generated by the LLMs while analyzing 125 clinical cases. To mitigate the risk of AI-generated fabrications, we propose that uncertainty scoring could be assigned to each AI-generated response. If the model's confidence falls below a predefined threshold (e.g., 90%), it should proactively decline to provide an answer. Furthermore, during the construction of reference databases, only rigorously vetted and validated data sources should be utilized. This disturbing situation is likely to be gradually ameliorated along with the advancement of AI technology. But for now, neither DeepSeek nor ChatGPT can fully substitute for professional medical personnel; they can merely serve as tools to assist patients in making decisions. In the present study, the two AI chatbots also frequently underlined the significance of consulting healthcare professionals.

Typically, while providing professional medical technical services for patients, clinicians offer humanistic care, which constitutes an important aspect of medical practice. In this study, DeepSeek expressed encouragement or consolation to users more frequently, while the responses of ChatGPT seldom included such content. In other studies, it has been proved that ChatGPT has difficulty taking into account the psychological needs of users (30, 31). Our results suggest that DeepSeek is superior to ChatGPT in terms of humanistic care. Nevertheless, more research is still required to confirm this.

This study also presents several limitations. Firstly, this study merely compared DeepSeek with ChatGPT-4.0. There have been numerous accessible AI chatbots, especially some specifically developed for medical applications, such as Google's Med-PaLM 2 (32). It is of great significance to compare DeepSeek with other AI chatbots. Moreover, since the LLMs evolve rapidly (33), the results may become outdated in the future. Secondly, this study did not evaluate the repeatability of the responses from the AI chatbots. Thirdly, the scoring tool adopted for evaluating the reliability and quality still partially relied on subjective judgment, thereby resulting in inevitable measurement bias. It is highly necessary to design a more objective assessment tool for the quality of AI responses. Fourthly, the responses of the AI chatbots were evaluated exclusively by urologists, thereby lacking input from health professionals in other relevant domains. Finally, this study focused on the responses of DeepSeek and ChatGPT-4.0 to issues related to UI, which restricted the universality of the study findings. More studies are required in the future to explore the performance of DeepSeek in other medical domains.

## 5 Conclusions

In accordance with the AUA/SUFU and EAU guidelines, DeepSeek and ChatGPT-4.0 might have comparable reliability in answering UI-related questions, though both lacked sufficient references. Nevertheless, the responses of DeepSeek outperform those of ChatGPT-4.0 in terms of feasibility and readability. When responding to clinical case questions, the responses of both AI chatbots adhered well to the guidelines, but there might be content not encompassed by the guidelines. The responses of DeepSeek contained more elements reflecting humanistic care compared to those of ChatGPT-4.0. These research findings underline the necessity for continuous refinement of AI chatbots when applied in the medical domain to enhance the readability of responses, the standardization of citing references, and the extent of humanistic care.

## Data Availability

The original contributions presented in the study are included in the article/Supplementary material, further inquiries can be directed to the corresponding authors.
