# Supplementary material for: Battle of the artificial intelligence: a comprehensive comparative analysis of DeepSeek and ChatGPT for urinary incontinence-related questions
Source: Front Public Health. 2025 Jul 23;13:1605908. doi: 10.3389/fpubh.2025.1605908 (PMC12325333; doi:10.3389/fpubh.2025.1605908)
Supplement: Supplementary file 1 [file Data_Sheet_1.zip › Supplementary materials/Supplementary Table 1.docx]

| **Supplementary Table 1.** ChatGPT-4.0 and DeepSeek’s responses to urinary incontinence-related questions |
| --- |
| 1. **What is urinary incontinence in women?** |
| **ChatGPT-4.0:**  Urinary incontinence in women refers to the involuntary leakage of urine, which can vary in severity from occasional dribbling to frequent and uncontrolled urination. It is a common condition that can affect women of all ages, but it is more prevalent in older women. Several factors can contribute to urinary incontinence in women, including:  1. **Pregnancy and Childbirth**: The physical changes and stress on the pelvic floor muscles during pregnancy and childbirth can weaken these muscles, leading to stress incontinence.  2. **Menopause**: Decreased levels of estrogen during menopause can affect the tissues of the bladder and urethra, potentially leading to incontinence.  3. **Pelvic Floor Disorders**: Conditions such as pelvic organ prolapse, where the bladder, uterus, or rectum drops from its normal position, can contribute to incontinence.  4. **Urinary Tract Infections (UTIs)**: UTIs can irritate the bladder, leading to temporary episodes of incontinence.  5. **Obesity**: Excess weight can increase abdominal pressure, which can contribute to stress incontinence.  6. **Neurological Disorders**: Conditions that affect nerve function, such as multiple sclerosis or Parkinson’s disease, can impact bladder control.  7. **Certain Medications**: Diuretics and other medications can increase urine production or affect bladder function.  The types of urinary incontinence that women may experience include stress incontinence, urge incontinence, mixed incontinence, overflow incontinence, and functional incontinence. Treatment options vary depending on the type and severity of incontinence and may include lifestyle changes, pelvic floor exercises, medications, medical devices, or surgery.  Women experiencing symptoms of urinary incontinence should consult a healthcare professional for an accurate diagnosis and appropriate treatment plan. Early intervention can help manage symptoms and improve quality of life. |
| **DeepSeek-V3:**  Urinary incontinence in women is the involuntary leakage of urine, which can occur due to various factors affecting the bladder, urethra, or pelvic floor muscles. It is a common condition that can significantly impact quality of life, but it is often treatable. Here’s an overview of urinary incontinence in women:  ###**Types of Urinary Incontinence in Women**  1. **Stress Incontinence**  - Leakage occurs during activities that increase abdominal pressure, such as coughing, sneezing, laughing, or exercising.  - Common causes: Weakening of pelvic floor muscles or the urethral sphincter, often due to childbirth, menopause, or aging.  2. **Urge Incontinence (Overactive Bladder)**  - Sudden, intense urge to urinate followed by involuntary leakage.  - Common causes: Overactivity of the bladder muscles, often due to neurological conditions, infections, or bladder irritation.  3. **Mixed Incontinence**  - Combination of stress and urge incontinence, where symptoms of both types are present.  - Common causes: Multiple factors contributing to both types of incontinence.  4. **Overflow Incontinence**  - Leakage occurs because the bladder doesn’t empty completely, leading to constant dribbling.  - Less common in women but can occur due to bladder muscle weakness or blockages.  5. **Functional Incontinence**  - Inability to reach the toilet in time due to physical or cognitive impairments (e.g., arthritis, dementia).  - Common causes: Mobility issues, environmental barriers, or cognitive decline.  ### **Causes of Urinary Incontinence in Women**  - **Childbirth**: Vaginal delivery can weaken pelvic floor muscles and damage nerves controlling the bladder.  - **Menopause**: Decreased estrogen levels can lead to thinning of the urethral lining and weakened pelvic muscles.  - **Aging**: Loss of muscle tone and bladder capacity increases the risk of incontinence.  - **Pelvic Organ Prolapse**: Weakening of pelvic organs (e.g., bladder, uterus) can lead to pressure on the bladder and incontinence.  - **Obesity**: Excess weight increases pressure on the bladder and pelvic floor.  - **Chronic Conditions**: Diabetes, neurological disorders (e.g., multiple sclerosis), or urinary tract infections can contribute.  - **Surgery**: Pelvic or bladder surgeries may affect bladder control.  ### **Symptoms**  - Involuntary leakage of urine during activities or at rest.  - Frequent urination or sudden urges to urinate.  - Difficulty emptying the bladder completely.  - Nocturia (frequent urination at night).  ### **Diagnosis**  A healthcare provider may perform:  - A detailed medical history and physical exam.  - Bladder diary to track urination patterns.  - Urinalysis to rule out infections.  - Urodynamic testing to assess bladder function.  - Imaging (e.g., ultrasound) to evaluate the bladder and pelvic organs.  ### **Treatment Options**  1. **Lifestyle Changes**:  - Weight loss, fluid management, and avoiding bladder irritants (e.g., caffeine, alcohol).  2. **Pelvic Floor Exercises (Kegels)**:  - Strengthen pelvic floor muscles to improve bladder control.  3. **Behavioral Therapies**:  - Bladder training to increase the time between urination.  4. **Medications**:  - Anticholinergics or beta-3 agonists to relax the bladder and reduce urgency.  5. **Medical Devices**:  - Pessaries to support the bladder or urethra.  6. **Minimally Invasive Procedures**:  - Botox injections, nerve stimulation, or bulking agents.  7. **Surgery**:  - Procedures like sling surgery or bladder suspension to support the urethra.  ### **Prevention**  - Maintain a healthy weight.  - Perform regular pelvic floor exercises.  - Avoid smoking, which can cause chronic coughing and weaken pelvic muscles.  - Treat chronic conditions like diabetes or UTIs promptly.  Urinary incontinence is not an inevitable part of aging, and many women find relief with proper diagnosis and treatment. Consulting a healthcare provider is essential for personalized care. |
| 1. **What are the types of urinary incontinence?** |
| **ChatGPT-4.0:**  Urinary incontinence is classified into several types, each characterized by different symptoms and causes. The primary types are:  1. **Stress Incontinence**: This occurs when urine leaks due to pressure exerted on the bladder by physical activities such as coughing, sneezing, laughing, exercising, or lifting heavy objects. It is often related to weakened pelvic floor muscles or a weakened urethral sphincter.  2. **Urge Incontinence**: Also known as overactive bladder, this type is characterized by a sudden and intense urge to urinate, followed by involuntary leakage of urine. People with urge incontinence may feel the need to urinate frequently, including multiple times during the night.  3. **Mixed Incontinence**: This type involves symptoms of both stress and urge incontinence. Individuals experience both the leakage associated with physical activity and the sudden urge to urinate.  4. **Overflow Incontinence**: This occurs when the bladder does not empty completely, leading to frequent or constant dribbling of urine. It can be caused by a weak bladder muscle or an obstruction that prevents normal bladder emptying.  5. **Functional Incontinence**: This type is related to physical or cognitive impairments that prevent a person from reaching the bathroom in time. Conditions such as severe arthritis, dementia, or mobility issues can contribute to functional incontinence.  6. **Transient Incontinence**: This is a temporary form of incontinence caused by specific conditions or factors, such as urinary tract infections, certain medications, constipation, or acute illnesses. It typically resolves when the underlying issue is treated.  Understanding the type of urinary incontinence is crucial for determining the appropriate treatment, so individuals experiencing symptoms should consult with a healthcare professional for a proper diagnosis and management plan. |
| **DeepSeek-V3:**  Urinary incontinence, the involuntary leakage of urine, can be categorized into several types based on the underlying cause and symptoms. The main types include:  1. **Stress Incontinence**  - Leakage occurs during activities that increase abdominal pressure, such as coughing, sneezing, laughing, or exercising.  - Common causes: Weakness of the pelvic floor muscles or urethral sphincter, often due to childbirth, surgery, or aging.  2. **Urge Incontinence (Overactive Bladder) **  - Sudden, intense urge to urinate followed by involuntary leakage.  - Common causes: Overactivity of the bladder muscles, often due to neurological conditions, infections, or bladder irritation.  3. **Overflow Incontinence**  - Leakage occurs because the bladder doesn’t empty completely, leading to constant dribbling.  - Common causes: Blockage (e.g., enlarged prostate), nerve damage, or weak bladder muscles.  4. **Functional Incontinence**  - Inability to reach the toilet in time due to physical or cognitive impairments (e.g., arthritis, dementia).  - Common causes: Mobility issues, environmental barriers, or cognitive decline.  5. **Mixed Incontinence**  - Combination of stress and urge incontinence, where symptoms of both types are present.  - Common causes: Multiple factors contributing to both types of incontinence.  6. **Reflex Incontinence**  - Leakage occurs without warning or sensation due to impaired nerve signals between the bladder and brain.  - Common causes: Spinal cord injury, multiple sclerosis, or other neurological disorders.  7. **Transient Incontinence**  - Temporary incontinence caused by reversible factors such as urinary tract infections, medications, or acute illnesses.  - Common causes: Infections, certain drugs (e.g., diuretics), or temporary conditions like constipation.  Identifying the type of incontinence is crucial for effective treatment, which may include lifestyle changes, pelvic floor exercises, medications, or surgery. A healthcare provider can help diagnose and manage the condition. |
| 1. **What are the causes of incontinence in women?** |
| **ChatGPT-4.0:**  Urinary incontinence in women can result from a variety of causes, often related to physical changes, medical conditions, or lifestyle factors. Here are some common causes:  1. **Pregnancy**: The weight of the growing fetus can put pressure on the bladder, leading to stress incontinence. Hormonal changes during pregnancy can also contribute to this condition.  2. **Childbirth**: Vaginal delivery can weaken pelvic floor muscles and damage nerves and supportive tissue, leading to incontinence. The risk may be higher with multiple births, prolonged labor, or the use of forceps.  3. **Menopause**: The decline in estrogen levels during menopause can lead to changes in the tissues of the bladder and urethra, which may contribute to incontinence.  4. **Pelvic Floor Disorders**: Conditions such as pelvic organ prolapse can occur when pelvic organs drop from their normal position, often leading to incontinence.  5. **Urinary Tract Infections (UTIs)**: UTIs can irritate the bladder, causing a strong urge to urinate and sometimes leading to incontinence.  6. **Obesity**: Excess body weight increases abdominal pressure, which can stress the bladder and lead to stress incontinence.  7. **Neurological Disorders**: Diseases such as multiple sclerosis, Parkinson’s disease, or spinal cord injuries can interfere with nerve signals involved in bladder control.  8. **Chronic Coughing**: Conditions that cause chronic coughing, such as smoking or lung disease, can put pressure on the bladder, leading to stress incontinence.  9. **Constipation**: Chronic constipation can cause the rectum to be constantly full, which puts pressure on the bladder and can affect its function.  10. **Certain Medications**: Diuretics, sedatives, and some blood pressure medications can affect bladder control.  11. **Age**: As women age, the muscles in the bladder and urethra can weaken, making it more difficult to control urination.  12. **Surgery or Trauma**: Surgeries involving the reproductive organs or pelvic area can sometimes damage the muscles or nerves involved in bladder control.  Understanding the underlying cause of urinary incontinence is important for determining the most effective treatment. Women experiencing symptoms should seek advice from a healthcare professional for a thorough evaluation and personalized management plan. |
| **DeepSeek-V3:**  Urinary incontinence in women can result from a variety of physical, hormonal, and lifestyle factors. Here are the primary causes:  ### **1. Childbirth**  - **Vaginal Delivery**: Can weaken pelvic floor muscles and damage nerves controlling the bladder.  - **Multiple Pregnancies**: Increases the risk of pelvic floor dysfunction.  ### **2. Menopause**  - **Decreased Estrogen Levels**: Leads to thinning of the urethral lining and weakening of pelvic muscles, reducing bladder control.  ### **3. Aging**  - **Loss of Muscle Tone**: Pelvic floor muscles and bladder muscles weaken over time.  - **Reduced Bladder Capacity**: The bladder holds less urine as it ages.  ### **4. Pelvic Organ Prolapse**  - **Weakening of Pelvic Organs**: The bladder, uterus, or rectum may drop from their normal position, putting pressure on the bladder and causing leakage.  ### **5. Obesity**  - **Increased Abdominal Pressure**: Excess weight puts additional pressure on the bladder and pelvic floor muscles.  ### **6. Chronic Conditions**  - **Diabetes**: Can cause nerve damage (neuropathy) affecting bladder control.  - **Neurological Disorders**: Conditions like multiple sclerosis, Parkinson’s disease, or stroke can impair nerve signals to the bladder.  - **Chronic Coughing**: From smoking or conditions like asthma can increase abdominal pressure and weaken pelvic muscles.  ### **7. Urinary Tract Infections (UTIs)**  - **Bladder Irritation**: Can cause temporary incontinence, especially urge incontinence.  ### **8. Surgery**  - **Pelvic or Bladder Surgery**: May damage nerves or muscles involved in bladder control.  - **Hysterectomy**: Can affect pelvic floor support.  ### **9. Medications**  - **Diuretics**: Increase urine production, leading to urgency and leakage.  - **Sedatives or Muscle Relaxants**: Can relax the bladder muscles too much.  - **Certain Antidepressants or Antihistamines**: May affect bladder control.  ### **10. Lifestyle Factors**  - **High Fluid Intake**: Can overwhelm bladder capacity.  - **Bladder Irritants**: Caffeine, alcohol, spicy foods, and acidic foods can irritate the bladder.  - **Physical Inactivity**: Weakens pelvic floor muscles over time.  ### **11. Constipation**  - **Straining**: Chronic constipation can weaken pelvic floor muscles and increase pressure on the bladder.  ### **12. Hormonal Changes**  - **Pregnancy**: Hormonal shifts and pressure from the growing uterus can lead to temporary incontinence.  - **Menstrual Cycle**: Some women experience bladder sensitivity during certain phases.  ### **13. Psychological Factors**  - **Stress or Anxiety**: Can exacerbate symptoms of incontinence, particularly urge incontinence.  ### **14. Genetic Factors**  - **Family History**: A predisposition to weak pelvic floor muscles or bladder issues may increase the risk.  ### **15. Physical Trauma or Injury**  - **Pelvic Injury**: Can damage nerves or muscles involved in bladder control.  ### **16. Overactive Bladder (OAB)**  - **Idiopathic Causes**: Sometimes the exact cause of bladder overactivity is unknown.  ### **Prevention and Management**  - **Pelvic Floor Exercises (Kegels)**: Strengthen pelvic muscles.  - **Healthy Weight Management**: Reduces pressure on the bladder.  - **Avoiding Bladder Irritants**: Minimize caffeine, alcohol, and spicy foods.  - **Treating Chronic Conditions**: Manage diabetes, UTIs, or constipation promptly.  Consulting a healthcare provider is essential for diagnosing the specific cause and developing an effective treatment plan tailored to individual needs. |
| 1. **Why do I sometimes leak urine when I cough or sneeze?** |
| **ChatGPT-4.0:**  Leaking urine when you cough or sneeze is a common symptom of a type of urinary incontinence known as stress incontinence. This condition occurs when physical activities or movements put pressure on your bladder, leading to involuntary leakage of urine. Here are some reasons why this might happen:  1. **Weak Pelvic Floor Muscles**: The pelvic floor muscles support the bladder and help maintain continence. If these muscles are weakened, they may not be able to effectively support the bladder during activities that increase abdominal pressure, such as coughing or sneezing.  2. **Childbirth**: Vaginal delivery can stretch and weaken the pelvic floor muscles, and potentially damage nerves and supportive tissues, increasing the risk of stress incontinence.  3. **Aging**: As you age, muscles, including those in the pelvic floor, can lose strength and elasticity, making it harder to control urination.  4. **Menopause**: The decrease in estrogen levels during menopause can lead to changes in the tissues of the bladder and urethra, contributing to stress incontinence.  5. **Obesity**: Excess body weight can increase pressure on the bladder and surrounding muscles, exacerbating stress incontinence.  6. **Previous Pelvic Surgery**: Surgeries involving the pelvic region can sometimes affect the muscles and nerves that control bladder function.  If you're experiencing stress incontinence, it's a good idea to consult with a healthcare professional. They can help determine the underlying cause and recommend appropriate treatments, which may include pelvic floor exercises (like Kegel exercises), lifestyle changes, medications, or, in some cases, surgical interventions. Early intervention can help manage symptoms and improve your quality of life. |
| **DeepSeek-V3:**  Leaking urine when you cough or sneeze is a classic symptom of **stress urinary incontinence (SUI)**, which is one of the most common types of incontinence in women. This occurs when physical activities that increase abdominal pressure—like coughing, sneezing, laughing, or exercising—cause urine to leak involuntarily. Here’s why this happens:  ---  ### **Causes of Leaking Urine When Coughing or Sneezing**  1. **Weakened Pelvic Floor Muscles**  - The pelvic floor muscles support the bladder, urethra, and other pelvic organs. When these muscles are weakened, they can’t properly control the release of urine during increased abdominal pressure.  - Common causes of weakened pelvic floor muscles:  - **Childbirth** (especially vaginal delivery).  - **Aging** (loss of muscle tone over time).  - **Menopause** (decreased estrogen levels weaken pelvic tissues).  - **Lack of Exercise** (inactivity can lead to muscle weakness).  2. **Damaged Urethral Sphincter**  - The urethral sphincter is a muscle that helps keep the urethra closed to prevent urine leakage. If this muscle is damaged or weakened, it may not function properly during activities like coughing or sneezing.  - Damage can occur due to:  - **Childbirth**.  - **Pelvic Surgery** (e.g., hysterectomy).  - **Chronic Straining** (e.g., from constipation or heavy lifting).  3. **Increased Abdominal Pressure**  - Activities like coughing, sneezing, laughing, or lifting heavy objects increase pressure on the bladder. If the pelvic floor muscles or urethral sphincter are weak, this pressure can force urine out.  4. **Pelvic Organ Prolapse**  - Weakening of the pelvic organs (e.g., bladder, uterus, or rectum) can cause them to drop from their normal position, putting pressure on the bladder and urethra and leading to leakage.  5. **Obesity**  - Excess weight increases pressure on the bladder and pelvic floor, making leakage more likely during activities that strain the abdomen.  6. **Chronic Coughing**  - Conditions like asthma, smoking, or chronic bronchitis can lead to frequent coughing, which repeatedly stresses the pelvic floor muscles and increases the risk of leakage.  ---  ### **What You Can Do**  1. **Pelvic Floor Exercises (Kegels)**  - Strengthening the pelvic floor muscles can help improve bladder control.  - To perform Kegels:  - Tighten the muscles you use to stop the flow of urine.  - Hold for 5–10 seconds, then relax.  - Repeat 10–15 times, 3 times daily.  2. **Lifestyle Changes**  - Maintain a healthy weight to reduce pressure on the bladder.  - Avoid bladder irritants like caffeine, alcohol, and spicy foods.  - Treat chronic coughing (e.g., quit smoking, manage asthma).  3. **Bladder Training**  - Gradually increase the time between bathroom visits to improve bladder control.  4. **Medical Treatments**  - **Hormone Therapy**: For postmenopausal women, estrogen creams or tablets can help strengthen pelvic tissues.  - **Medications**: Certain drugs can help tighten the urethral sphincter.  - **Pessary**: A device inserted into the vagina to support the bladder and urethra.  5. **Surgical Options**  - **Sling Surgery**: A mesh sling is placed under the urethra to provide support.  - **Bladder Suspension Surgery**: Repositions the bladder and urethra to reduce leakage.  ---  ### **When to See a Doctor**  If leaking urine during coughing or sneezing:  - Interferes with your daily life.  - Occurs frequently or worsens over time.  - Is accompanied by pain, frequent urination, or blood in the urine.  A healthcare provider can assess the underlying cause and recommend appropriate treatments. Stress urinary incontinence is highly treatable, and many women find significant relief with the right approach. |
| 1. **Why do I have a strong desire to urinate, but have not yet reached the toilet to leak urine?** |
| **ChatGPT-4.0:**  The situation you're describing sounds like urge incontinence, which is characterized by a sudden, intense urge to urinate followed by an involuntary loss of urine. This condition is often associated with overactive bladder syndrome. Here are some potential reasons for experiencing urge incontinence:  1. **Overactive Bladder**: This condition involves involuntary bladder contractions, which can create a sudden and strong urge to urinate. These contractions can occur even when the bladder is not full.  2. **Urinary Tract Infections (UTIs)**: Infections can irritate the bladder lining, leading to a strong and sudden urge to urinate.  3. **Neurological Disorders**: Conditions such as Parkinson's disease, multiple sclerosis, or stroke can interfere with nerve signals involved in bladder control, leading to urge incontinence.  4. **Bladder Irritants**: Certain foods and drinks, such as caffeine, alcohol, spicy foods, and citrus fruits, can irritate the bladder and exacerbate symptoms of urge incontinence.  5. **Medications**: Some medications, including diuretics and certain sedatives, can increase urine production or affect bladder function.  6. **Bladder Stones or Tumors**: These can irritate the bladder and lead to symptoms of urgency and frequent urination.  If you are experiencing urge incontinence, it is important to consult a healthcare professional for an accurate diagnosis and appropriate treatment plan. Management strategies may include lifestyle modifications, bladder training exercises, medications, or other interventions tailored to your specific situation. |
| **DeepSeek-V3:**  A strong, sudden urge to urinate followed by leakage before reaching the toilet is a hallmark symptom of **urge incontinence**, also known as **overactive bladder (OAB)**. This occurs when the bladder muscles contract involuntarily, creating an intense need to urinate even when the bladder isn’t full. Here’s why this happens and what you can do about it:  ---  ### **Causes of Urge Incontinence**  1. **Overactive Bladder Muscles**  - The bladder muscles (detrusor muscles) may contract too frequently or too strongly, even when the bladder isn’t full. This can be due to:  - **Nerve Damage**: Conditions like diabetes, multiple sclerosis, Parkinson’s disease, or stroke can disrupt nerve signals controlling the bladder.  - **Idiopathic Causes**: In many cases, the exact cause is unknown.  2. **Bladder Irritation**  - Certain factors can irritate the bladder lining, triggering involuntary contractions:  - **Urinary Tract Infections (UTIs)**: A common cause of temporary urge incontinence.  - **Bladder Stones or Tumors**: These can irritate the bladder and cause overactivity.  - **Bladder Irritants**: Caffeine, alcohol, spicy foods, acidic foods, and artificial sweeteners can irritate the bladder.  3. **Menopause**  - Decreased estrogen levels can lead to thinning of the bladder and urethral lining, increasing sensitivity and overactivity.  4. **Aging**  - Bladder muscles may become less elastic and more prone to involuntary contractions as you age.  5. **Medications**  - Diuretics (water pills) increase urine production, while certain medications (e.g., sedatives or muscle relaxants) can affect bladder control.  6. **Chronic Conditions**  - **Constipation**: Pressure from a full bowel can irritate the bladder.  - **Obesity**: Excess weight increases pressure on the bladder.  7. **Psychological Factors**  - Stress, anxiety, or a heightened awareness of bladder sensations can exacerbate urge incontinence.  ---  ### **What You Can Do**  1. **Bladder Training**  - Gradually increase the time between bathroom visits to retrain your bladder to hold more urine.  - Start by delaying urination by 5–10 minutes and gradually extend the time.  2. **Pelvic Floor Exercises (Kegels)**  - Strengthening the pelvic floor muscles can help improve bladder control and reduce leakage.  3. **Lifestyle Changes**  - Avoid bladder irritants like caffeine, alcohol, and spicy foods.  - Maintain a healthy weight to reduce pressure on the bladder.  - Stay hydrated, but avoid excessive fluid intake.  4. **Double Voiding**  - After urinating, wait a few moments and try again to ensure the bladder is fully empty.  5. **Medical Treatments**  - **Medications**: Anticholinergic or beta-3 agonist drugs can relax the bladder muscles and reduce overactivity.  - **Botox Injections**: Injected into the bladder muscle to reduce involuntary contractions.  - **Nerve Stimulation**: Techniques like sacral neuromodulation or percutaneous tibial nerve stimulation (PTNS) can help regulate bladder function.  6. **Hormone Therapy**  - For postmenopausal women, estrogen creams or tablets can help strengthen pelvic tissues and reduce bladder sensitivity.  ---  ### **When to See a Doctor**  If you experience:  - Frequent, sudden urges to urinate.  - Leakage before reaching the toilet.  - Pain, blood in the urine, or signs of a UTI (e.g., burning, fever).  A healthcare provider can perform tests (e.g., urinalysis, urodynamic studies) to determine the underlying cause and recommend appropriate treatments. Urge incontinence is manageable, and many people find significant relief with the right approach. |
| 1. **Which medical specialist should I seek help from to treat urinary incontinence?** |
| **ChatGPT-4.0:**  To treat urinary incontinence, you should consider seeing one of the following medical specialists:  1. **Urologist**: These specialists focus on the urinary tract and male reproductive system. They are experienced in diagnosing and treating urinary incontinence for both men and women.  2. **Gynecologist**: Women experiencing urinary incontinence, especially if related to childbirth, menopause, or pelvic floor issues, may benefit from seeing a gynecologist.  3. **Urogynecologist**: This specialist combines expertise in both urology and gynecology, focusing on female pelvic medicine and reconstructive surgery. They are particularly skilled in treating urinary incontinence and pelvic floor disorders in women.  4. **Geriatrician**: If you are older, a geriatrician can be helpful, especially if the incontinence is part of broader age-related health concerns.  5. **Physical Therapist**: Some physical therapists specialize in pelvic floor rehabilitation and can provide exercises to strengthen pelvic muscles and improve bladder control.  Starting with your primary care physician is a good approach. They can assess your symptoms, rule out underlying conditions, and refer you to the appropriate specialist for further evaluation and treatment. |
| **DeepSeek-V3:**  If you’re experiencing **urinary incontinence**, the right medical specialist depends on the type and cause of your condition, as well as your gender and overall health. Here’s a breakdown of the specialists who can help and when to see them:  ### **1. Primary Care Physician (PCP)**  - **Role**: Your PCP is often the first point of contact. They can evaluate your symptoms, perform initial tests, and refer you to a specialist if needed.  - **When to See Them**: If you’re unsure where to start or if your symptoms are mild.  ### **2. Urologist**  - **Role**: A urologist specializes in the urinary tract (bladder, kidneys, urethra) and male reproductive system. They diagnose and treat conditions like overactive bladder, stress incontinence, and bladder infections.  - **When to See Them**:  - If you have complex or persistent urinary incontinence.  - If you suspect bladder or prostate issues (in men).  - If you’re considering surgical options.  ### **3. Urogynecologist**  - **Role**: A urogynecologist is a subspecialist who focuses on pelvic floor disorders in women, including urinary incontinence, pelvic organ prolapse, and bladder issues.  - **When to See Them**:  - If you’re a woman with stress incontinence, urge incontinence, or pelvic organ prolapse.  - If you’ve had multiple pregnancies or childbirth-related pelvic floor issues.  ### **4. Gynecologist**  - **Role**: A gynecologist specializes in women’s reproductive health and can address incontinence related to hormonal changes, childbirth, or pelvic floor dysfunction.  - **When to See Them**:  - If your incontinence is related to menopause, pregnancy, or childbirth.  - If you’re experiencing other gynecological symptoms.  ### **5. Physical Therapist (Pelvic Floor Specialist)**  - **Role**: A physical therapist trained in pelvic floor rehabilitation can help strengthen pelvic floor muscles through exercises (e.g., Kegels), biofeedback, and other techniques.  - **When to See Them**:  - If you have mild to moderate incontinence and want to avoid surgery or medication.  - If you’re recovering from childbirth or pelvic surgery.  ### **6. Geriatrician**  - **Role**: A geriatrician specializes in the health of older adults and can address incontinence related to aging, medications, or chronic conditions.  - **When to See Them**:  - If you’re an older adult with incontinence and multiple health concerns.  ### **7. Neurologist**  - **Role**: A neurologist treats conditions affecting the nervous system, which can impact bladder control (e.g., multiple sclerosis, Parkinson’s disease, or spinal cord injuries).  - **When to See Them**:  - If your incontinence is related to nerve damage or neurological conditions.  ### **How to Choose the Right Specialist**  - **Start with Your PCP**: They can guide you to the most appropriate specialist based on your symptoms and medical history.  - **Consider Your Symptoms**:  - **Stress incontinence** (leaking during coughing, sneezing, or exercise): Urogynecologist or urologist.  - **Urge incontinence** (sudden, intense urge to urinate): Urologist or urogynecologist.  - **Pelvic floor issues**: Pelvic floor physical therapist or urogynecologist.  - **Neurological causes**: Neurologist.  ### **What to Expect During Your Visit**  - **Medical History**: Be prepared to discuss your symptoms, lifestyle, and medical history.  - **Physical Exam**: The specialist may perform a pelvic exam (for women) or a prostate exam (for men).  - **Tests**: These may include:  - Urinalysis (to check for infection).  - Bladder diary (to track fluid intake and urination patterns).  - Urodynamic testing (to assess bladder function).  - Imaging (e.g., ultrasound or MRI).  ### **Next Steps**  - **Treatment Options**: Depending on the cause, treatment may include lifestyle changes, pelvic floor exercises, medications, or surgery.  - **Follow-Up**: Regular follow-ups with your specialist can help monitor progress and adjust treatment as needed.  ### **Quick Summary**  - **Women**: Start with a **gynecologist** or **urogynecologist**.  - **Men**: Start with a **urologist**.  - **Pelvic Floor Issues**: See a **pelvic floor physical therapist**.  - **Neurological Causes**: See a **neurologist**.  - **Older Adults**: Consider a **geriatrician**.  Urinary incontinence is a common and treatable condition, so don’t hesitate to seek help from the appropriate specialist. |
| 1. **What is the most recommended treatment for stress urinary incontinence?** |
| **ChatGPT-4.0:**  The treatment for stress urinary incontinence (SUI) often depends on the severity of the condition, the underlying causes, and the patient's overall health and preferences. Here are some commonly recommended treatments:  1. **Pelvic Floor Exercises (Kegel Exercises)**: Strengthening the pelvic floor muscles can help improve bladder control. These exercises are often recommended as a first-line treatment for stress urinary incontinence.  2. **Lifestyle Modifications**: Changes such as losing weight, reducing caffeine and alcohol intake, quitting smoking, and avoiding heavy lifting can help reduce symptoms.  3. **Bladder Training**: This involves scheduling bathroom visits and gradually increasing the time between them to improve bladder control.  4. **Physical Therapy**: Specialized physical therapists can provide guidance on pelvic floor muscle training and other techniques to improve bladder control.  5. **Pessaries**: These are devices inserted into the vagina to support the bladder and urethra, which can help reduce leakage in women.  6. **Medications**: While there are no specific medications approved for stress urinary incontinence, some off-label options might be considered in certain cases.  7. **Surgical Options**: If conservative treatments are not effective, surgical procedures may be considered. Common surgeries include sling procedures, which provide support to the urethra, and colposuspension, which lifts the bladder neck and urethra.  8. **Bulking Agents**: These are injections that can be used to bulk up the area around the urethra, helping it to close more effectively.  It's essential to consult a healthcare professional to determine the most appropriate treatment based on individual circumstances. They can provide a comprehensive evaluation and tailor a treatment plan to your specific needs. |
| **DeepSeek-V3:**  The most recommended treatment for **stress urinary incontinence (SUI)** depends on the severity of your symptoms, your overall health, and your preferences. SUI occurs when physical activities like coughing, sneezing, laughing, or exercising put pressure on the bladder, causing leakage. Here’s an overview of the most effective treatments:  ### **1. Lifestyle Modifications**  - **Weight Loss**: If you’re overweight, losing weight can reduce pressure on the bladder and pelvic floor.  - **Smoking Cessation**: Smoking can cause chronic coughing, which worsens SUI.  - **Fluid Management**: Avoid excessive caffeine and alcohol, which can irritate the bladder.  ### **2. Pelvic Floor Muscle Training (Kegel Exercises)**  - **What It Is**: Strengthening the pelvic floor muscles to better support the bladder and urethra.  - **How It Helps**: Improves muscle tone and control, reducing leakage.  - **How to Do It**:  - Tighten the muscles you use to stop urination (hold for 5–10 seconds, then relax).  - Repeat 10–15 times, 3 times a day.  - **Professional Guidance**: A **pelvic floor physical therapist** can teach you proper techniques and may use biofeedback or electrical stimulation to enhance results.  ### **3. Bladder Training**  - **What It Is**: Gradually increasing the time between bathroom visits to improve bladder control.  - **How It Helps**: Helps retrain the bladder to hold more urine and reduces urgency.  ### **4. Medications**  While medications are more commonly used for **urge incontinence**, some may help with SUI:  - **Duloxetine**: An antidepressant that can increase urethral sphincter tone.  - **Hormone Therapy**: Estrogen creams or patches may help postmenopausal women with SUI by improving urethral and vaginal tissue health.  ### **5. Medical Devices**  - **Pessary**: A removable device inserted into the vagina to support the bladder and urethra.  - **Urethral Inserts**: Small, disposable devices placed in the urethra to prevent leakage during activities.  ### **6. Minimally Invasive Procedures**  - **Bulking Agents**: Injectable materials (e.g., collagen, silicone) are injected around the urethra to add bulk and improve closure.  - **Laser Therapy**: Vaginal laser treatments can improve tissue strength and reduce SUI symptoms in some women.  ### **7. Surgical Treatments**  Surgery is typically recommended for moderate to severe SUI that hasn’t responded to conservative treatments. Common options include:  - **Midurethral Sling**: A mesh tape is placed under the urethra to provide support. This is the most common and effective surgical treatment for SUI.  - **Burch Colposuspension**: The bladder neck is lifted and secured to nearby ligaments to reduce leakage.  - **Artificial Urinary Sphincter**: A device is implanted to control urine flow, typically used in men with SUI.  ### **How to Choose the Right Treatment**  - **Mild SUI**: Start with lifestyle changes, pelvic floor exercises, and bladder training.  - **Moderate SUI**: Consider pelvic floor therapy, pessaries, or bulking agents.  - **Severe SUI**: Surgery (e.g., midurethral sling) may be the best option.  ### **What to Expect**  - **Consultation**: Your doctor or specialist will evaluate your symptoms, perform a physical exam, and may order tests (e.g., urodynamic studies) to confirm SUI.  - **Follow-Up**: Regular follow-ups are important to monitor progress and adjust treatment as needed.  ### **Quick Summary**  - **First-Line Treatment**: Pelvic floor exercises and lifestyle changes.  - **Second-Line Treatment**: Pessaries, bulking agents, or medications.  - **Surgical Option**: Midurethral sling is the most common and effective surgery for SUI.  Stress urinary incontinence is highly treatable, so don’t hesitate to discuss these options with your **urologist**, **urogynecologist**, or **pelvic floor physical therapist**. |
| 1. **What is the most recommended treatment for urgency urinary incontinence?** |
| **ChatGPT-4.0:**  Urgency urinary incontinence, also known as overactive bladder (OAB), is characterized by a sudden, intense urge to urinate, often resulting in involuntary leakage. The treatment approach typically depends on the severity of symptoms, underlying causes, and patient preferences. Here are some commonly recommended treatments:  1. **Lifestyle Modifications**: These include reducing fluid intake, especially of bladder irritants like caffeine and alcohol, and managing fluid consumption to avoid large volumes at once.  2. **Bladder Training**: This involves scheduling regular bathroom visits and gradually increasing the time between them. It helps retrain the bladder to hold urine longer and reduce urgency.  3. **Pelvic Floor Exercises (Kegel Exercises)**: Strengthening the pelvic floor muscles can help improve bladder control and reduce symptoms of urgency.  4. **Medications**: Several medications can help manage symptoms of overactive bladder by relaxing the bladder muscle or reducing bladder contractions. Common classes include anticholinergics (such as oxybutynin and tolterodine) and beta-3 adrenergic agonists (such as mirabegron).  5. **Behavioral Therapies**: Techniques such as biofeedback can help patients become more aware of their pelvic floor muscles and improve control.  6. **Botox Injections**: OnabotulinumtoxinA (Botox) can be injected into the bladder muscle to reduce involuntary contractions, providing relief for some patients.  7. **Nerve Stimulation**: Techniques like sacral neuromodulation or percutaneous tibial nerve stimulation can help regulate nerve signals to the bladder, reducing urgency and frequency.  8. **Surgery**: In rare cases where other treatments are ineffective, surgical options may be considered to increase bladder capacity or reduce bladder pressure.  It's important to consult with a healthcare provider to determine the most appropriate treatment plan for your specific situation. They can conduct a thorough evaluation and tailor a treatment strategy that best addresses your symptoms and lifestyle. |
| **DeepSeek-V3:**  The most recommended treatment for **urgency urinary incontinence (UUI)**—also known as **overactive bladder (OAB)**—depends on the severity of your symptoms, underlying causes, and your preferences. UUI is characterized by a sudden, intense urge to urinate, often leading to involuntary leakage. Here’s an overview of the most effective treatments:  ### **1. Lifestyle Modifications**  - **Fluid Management**: Avoid excessive fluid intake, especially before bedtime, and limit bladder irritants like caffeine, alcohol, and spicy foods.  - **Weight Loss**: If overweight, losing weight can reduce pressure on the bladder.  - **Smoking Cessation**: Smoking can irritate the bladder and worsen symptoms.  ### **2. Bladder Training**  - **What It Is**: Gradually increasing the time between bathroom visits to improve bladder control.  - **How It Helps**: Helps retrain the bladder to hold more urine and reduces urgency.  - **How to Do It**:  - Start by delaying urination by 5–10 minutes when you feel the urge.  - Gradually increase the delay over weeks or months.  ### **3. Pelvic Floor Muscle Training (Kegel Exercises)**  - **What It Is**: Strengthening the pelvic floor muscles to improve bladder control.  - **How It Helps**: Enhances muscle tone and reduces urgency and leakage.  - **How to Do It**:  - Tighten the muscles you use to stop urination (hold for 5–10 seconds, then relax).  - Repeat 10–15 times, 3 times a day.  - **Professional Guidance**: A **pelvic floor physical therapist** can teach proper techniques and may use biofeedback or electrical stimulation to enhance results.  ### **4. Medications**  Medications are often prescribed to relax the bladder and reduce urgency. Common options include:  - **Anticholinergics**:  - Oxybutynin (Ditropan, Oxytrol)  - Tolterodine (Detrol)  - Solifenacin (Vesicare)  - Fesoterodine (Toviaz)  - **Beta-3 Agonists**:  - Mirabegron (Myrbetriq) – relaxes the bladder muscle and increases bladder capacity.  - **Side Effects**: Dry mouth, constipation, blurred vision, or dizziness.  ### **5. Botox Injections**  - **What It Is**: Botulinum toxin (Botox) is injected into the bladder muscle to relax it and reduce urgency.  - **How It Helps**: Can significantly reduce symptoms for several months.  - **When to Consider**: If medications and behavioral therapies are ineffective.  - **Side Effects**: Temporary difficulty urinating or urinary tract infections (UTIs).  ### **6. Neuromodulation**  - **What It Is**: Electrical stimulation of nerves that control bladder function.  - **Types**:  - **Percutaneous Tibial Nerve Stimulation (PTNS)**: A needle electrode stimulates the tibial nerve in the ankle.  - **Sacral Neuromodulation (InterStim)**: A device is implanted to stimulate the sacral nerves.  - **How It Helps**: Can improve bladder control and reduce urgency.  ### **7. Surgery**  Surgery is considered a last resort for severe UUI that hasn’t responded to other treatments. Options include:  - **Bladder Augmentation**: Increasing bladder capacity by adding a piece of intestine to the bladder.  - **Urinary Diversion**: Redirecting urine flow to an external pouch.  ### **8. Complementary Therapies**  - **Acupuncture**: May help reduce urgency and improve bladder control in some individuals.  - **Herbal Remedies**: Some people find relief with herbs like pumpkin seed extract or Gosha-jinki-gan, but evidence is limited.  ### **How to Choose the Right Treatment**  - **Mild UUI**: Start with lifestyle changes, bladder training, and pelvic floor exercises.  - **Moderate UUI**: Add medications or Botox injections.  - **Severe UUI**: Consider neuromodulation or surgery.  ### **What to Expect**  - **Consultation**: Your doctor or specialist will evaluate your symptoms, perform a physical exam, and may order tests (e.g., urodynamic studies, bladder diary) to confirm UUI.  - **Follow-Up**: Regular follow-ups are important to monitor progress and adjust treatment as needed.  ### **Quick Summary**  - **First-Line Treatment**: Bladder training, pelvic floor exercises, and lifestyle changes.  - **Second-Line Treatment**: Medications (anticholinergics or beta-3 agonists).  - **Third-Line Treatment**: Botox injections or neuromodulation.  - **Surgical Option**: Bladder augmentation or urinary diversion (rarely needed).  Urgency urinary incontinence is highly manageable, so discuss these options with your **urologist**, **urogynecologist**, or **pelvic floor physical therapist** to create a personalized treatment plan. |
| 1. **What are the surgical treatment methods for urinary incontinence and how to choose it?** |
| **ChatGPT-4.0:**  Surgical treatment for urinary incontinence is typically considered when conservative methods, such as lifestyle changes, pelvic floor exercises, and medications, have not been effective. The choice of surgical method depends on the type of incontinence, the severity of symptoms, the patient's overall health, and personal preferences. Here are some common surgical options:  1. **Mid-Urethral Sling Surgery**: This is a common procedure for stress urinary incontinence, especially in women. A sling, usually made of synthetic mesh, is placed under the mid-urethra to provide support and prevent leakage during activities that increase abdominal pressure, such as coughing or exercising.  2. **Burch Colposuspension**: This procedure involves lifting and securing the bladder neck and urethra to the pelvic bone using sutures. It's typically used for stress urinary incontinence and can be performed via open surgery or laparoscopically.  3. **Autologous Fascial Sling**: Similar to the mid-urethral sling, this procedure uses a strip of the patient's own tissue (usually from the abdomen or thigh) to create a sling for supporting the urethra.  4. **Artificial Urinary Sphincter (AUS)**: This is often used in men, particularly after prostate surgery. It involves implanting a device that mimics the function of the urinary sphincter, with a cuff placed around the urethra to control urine flow.  5. **Bulking Agents**: Injections of materials like collagen or synthetic substances can be used to bulk up the tissue around the urethra, improving its closure and reducing leakage. This is less invasive and often used for stress incontinence.  6. **Bladder Augmentation (Augmentation Cystoplasty)**: This is more common for urgency urinary incontinence when the bladder is overactive. It involves enlarging the bladder using a piece of the intestine to increase capacity and reduce pressure.  7. **Sacral Nerve Stimulation (Sacral Neuromodulation)**: A device is implanted to stimulate the sacral nerves, which can help regulate bladder function and is used for both urgency and stress incontinence.  **Choosing the Right Surgical Option:**  - **Type of Incontinence**: The type of incontinence (stress, urgency, or mixed) is a primary factor in determining the appropriate surgical intervention.  - **Severity and Impact**: The severity of symptoms and their impact on quality of life will influence the decision.  - **Health Status**: The patient's overall health, medical history, and any previous surgeries will be considered.  - **Risks and Benefits**: Each surgical option has its own risks and benefits, which should be discussed with a healthcare provider.  - **Recovery Time**: Consideration of the recovery period and how it fits into the patient's lifestyle and obligations.  - **Patient Preference**: The patient's personal preferences and expectations play a crucial role in the decision-making process.  It is essential to have a detailed discussion with a urologist or a specialist in female pelvic medicine and reconstructive surgery to explore all options, understand the potential outcomes, and make an informed decision. |
| **DeepSeek-V3:**  Surgical treatment for **urinary incontinence** is typically considered when conservative treatments (e.g., pelvic floor exercises, medications, behavioral therapies) have failed or when the condition is severe. The choice of surgical method depends on the **type of incontinence**, the underlying cause, the patient’s overall health, and their preferences. Below is an overview of the most common surgical options and how to choose the right one:  ### **1. Stress Urinary Incontinence (SUI) Surgical Treatments**  SUI occurs when physical activities like coughing, sneezing, or exercising cause urine leakage due to weakened pelvic floor muscles or urethral sphincter.  #### **a. Midurethral Sling (MUS)**  - **What It Is**: A synthetic mesh tape is placed under the urethra to provide support.  - **How It Helps**: The tape acts like a hammock, compressing the urethra during activities that increase abdominal pressure.  - **Success Rate**: 80–90% success rate.  - **Pros**: Minimally invasive, short recovery time.  - **Cons**: Rare risks include mesh erosion, infection, or urinary retention.  - **Best For**: Most women with SUI.  #### **b. Burch Colposuspension**  - **What It Is**: The bladder neck is lifted and sutured to nearby ligaments to support the urethra.  - **How It Helps**: Reduces urethral mobility and prevents leakage.  - **Success Rate**: 70–80% success rate.  - **Pros**: No mesh is used.  - **Cons**: Longer recovery time compared to sling procedures.  - **Best For**: Women who prefer non-mesh options or have complex cases.  #### **c. Urethral Bulking Agents**  - **What It Is**: Injectable materials (e.g., collagen, silicone) are injected around the urethra to add bulk and improve closure.  - **How It Helps**: Increases resistance to urine flow.  - **Success Rate**: 50–60% success rate.  - **Pros**: Minimally invasive, no major incisions.  - **Cons**: Effects may diminish over time, requiring repeat injections.  - **Best For**: Older women or those who are not candidates for more invasive surgery.  #### **d. Artificial Urinary Sphincter**  - **What It Is**: A device is implanted around the urethra to control urine flow.  - **How It Helps**: The patient can manually inflate or deflate the device to control urination.  - **Success Rate**: High success rate in men.  - **Pros**: Effective for severe SUI.  - **Cons**: Requires manual operation, risk of mechanical failure or infection.  - **Best For**: Men with SUI (e.g., after prostate surgery).  ### **2. Urgency Urinary Incontinence (UUI) Surgical Treatments**  UUI is caused by an overactive bladder, leading to sudden, uncontrollable urges to urinate.  #### **a. Botox Injections**  - **What It Is**: Botulinum toxin (Botox) is injected into the bladder muscle to relax it and reduce urgency.  - **How It Helps**: Reduces bladder contractions and increases bladder capacity.  - **Success Rate**: Effective for 6–9 months, may require repeat injections.  - **Pros**: Minimally invasive.  - **Cons**: Temporary effect, risk of urinary retention or UTIs.  - **Best For**: Patients who don’t respond to medications.  #### **b. Sacral Neuromodulation (InterStim)**  - **What It Is**: A device is implanted to stimulate the sacral nerves, which control bladder function.  - **How It Helps**: Modulates nerve signals to reduce bladder overactivity.  - **Success Rate**: 70–80% success rate.  - **Pros**: Reversible, adjustable.  - **Cons**: Requires surgery, risk of infection or device malfunction.  - **Best For**: Patients with refractory UUI.  #### **c. Bladder Augmentation (Augmentation Cystoplasty)**  - **What It Is**: A piece of intestine is added to the bladder to increase its capacity.  - **How It Helps**: Reduces urgency by allowing the bladder to hold more urine.  - **Success Rate**: High success rate for severe cases.  - **Pros**: Effective for severe UUI.  - **Cons**: Major surgery, risk of complications (e.g., UTIs, bowel issues).  - **Best For**: Patients with very small bladder capacity or severe refractory UUI.  #### **d. Urinary Diversion**  - **What It Is**: Urine is diverted to an external pouch (e.g., ileal conduit) or internally to a reservoir.  - **How It Helps**: Bypasses the bladder to prevent leakage.  - **Success Rate**: Effective for severe cases.  - **Pros**: Eliminates incontinence.  - **Cons**: Major surgery, requires lifelong management.  - **Best For**: Patients with severe, untreatable UUI or other bladder dysfunction.  ### **How to Choose the Right Surgical Treatment**  1. **Determine the Type of Incontinence**:  - **SUI**: Focus on urethral support (e.g., sling, Burch colposuspension).  - **UUI**: Focus on bladder control (e.g., Botox, neuromodulation).  2. **Evaluate Severity**:  - Mild to moderate cases may respond to less invasive options (e.g., bulking agents, Botox).  - Severe cases may require more invasive procedures (e.g., sling, bladder augmentation).  3. **Consider Patient Factors**:  - Age, overall health, and surgical risk.  - Preference for mesh vs. non-mesh options.  4. **Discuss Risks and Benefits**:  - Each procedure has unique risks (e.g., infection, mechanical failure) and benefits (e.g., success rate, recovery time).  5. **Consult a Specialist**:  - A **urologist** or **urogynecologist** can perform tests (e.g., urodynamic studies) and recommend the best option based on your specific case.  ### **Quick Summary**  - **SUI**: Midurethral sling (most common), Burch colposuspension, bulking agents, or artificial urinary sphincter.  - **UUI**: Botox, sacral neuromodulation, bladder augmentation, or urinary diversion.  Surgical treatment for urinary incontinence can significantly improve quality of life. Work with your healthcare provider to choose the best option for your needs. |
| 1. **When should surgery be performed in a woman with urinary incontinence?** |
| **ChatGPT-4.0:**  Surgery for urinary incontinence in women is typically considered when conservative treatments have not provided adequate relief. These treatments include lifestyle modifications, pelvic floor exercises, bladder training, and medications. Here are some specific situations in which surgery might be considered:  1. **Persistent Symptoms**: When non-surgical treatments have been tried consistently and symptoms of urinary incontinence remain bothersome and impact the woman’s quality of life.  2. **Type of Incontinence**: Surgery is more commonly considered for stress urinary incontinence (SUI) than for urgency urinary incontinence. Stress incontinence involves leakage during activities that increase abdominal pressure, such as coughing, sneezing, or exercising.  3. **Severity of Incontinence**: Severe cases where incontinence significantly affects daily activities, social interactions, or emotional well-being might warrant surgical intervention.  4. **Patient's Health and Preferences**: The overall health of the patient, her surgical risk profile, and personal preferences regarding surgery versus ongoing conservative management are important considerations.  5. **Impact on Quality of Life**: If urinary incontinence is causing significant distress, embarrassment, or limitations in personal, social, or professional life, surgery might be a viable option.  6. **Desire for Long-Term Solution**: Some women may prefer surgery for a potentially longer-term solution compared to ongoing management with non-surgical methods.  Before proceeding with surgery, a thorough evaluation by a healthcare provider, typically a urologist or gynecologist specializing in pelvic floor disorders, is essential. This evaluation often includes:  - A detailed medical history and physical examination.  - Urodynamic testing to assess bladder function and confirm the type of incontinence.  - Discussion of the risks, benefits, and potential outcomes of surgical options.  - Consideration of any previous pelvic surgeries or conditions that might influence surgical decisions.  Ultimately, the decision to proceed with surgery is a collaborative one, made between the patient and her healthcare provider, taking into account all medical, psychological, and lifestyle factors. |
| **DeepSeek-V3:**  Surgery for **urinary incontinence (UI)** in women is typically considered when **conservative treatments** (e.g., pelvic floor exercises, behavioral therapies, medications) have failed to provide adequate relief or when the condition significantly impacts the patient’s quality of life. The decision to proceed with surgery depends on the **type of incontinence**, the severity of symptoms, and the patient’s overall health and preferences. Below are the key indications and considerations for surgery:  ### **1. Indications for Surgery**  #### **a. Stress Urinary Incontinence (SUI)**  SUI occurs when physical activities like coughing, sneezing, or exercising cause urine leakage due to weakened pelvic floor muscles or urethral sphincter. Surgery is indicated when:  - Symptoms are **moderate to severe** and significantly affect daily life.  - Conservative treatments (e.g., pelvic floor exercises, pessaries) have failed.  - The patient desires a **long-term solution** rather than ongoing management.  #### **b. Urgency Urinary Incontinence (UUI)**  UUI is caused by an overactive bladder, leading to sudden, uncontrollable urges to urinate. Surgery is indicated when:  - Symptoms are **refractory to medications** (e.g., anticholinergics, beta-3 agonists) and behavioral therapies.  - The patient experiences **severe symptoms** (e.g., frequent leakage, nocturia) that disrupt their quality of life.  - **Neuromodulation** or **Botox injections** are considered as less invasive surgical options before more invasive procedures.  #### **c. Mixed Urinary Incontinence (MUI)**  MUI involves both SUI and UUI. Surgery is considered when:  - One type of incontinence (usually SUI) is the **dominant issue** and is amenable to surgical correction.  - Conservative treatments have failed for both components.  ### **2. Factors to Consider Before Surgery**  - **Type of Incontinence**: Surgery is most effective for **SUI**, while **UUI** often requires less invasive options (e.g., Botox, neuromodulation) before considering major surgery.  - **Severity of Symptoms**: Surgery is typically reserved for **moderate to severe** cases that significantly impact quality of life.  - **Patient’s Age and Health**:  - Younger, healthier patients may opt for surgery to achieve long-term relief.  - Older patients or those with comorbidities may prefer less invasive options.  - **Childbearing Plans**: Pregnancy and childbirth can affect surgical outcomes, so surgery may be delayed in women planning future pregnancies.  - **Previous Treatments**: Surgery is considered after **conservative treatments** have been tried and failed.  - **Patient’s Preferences**: The patient’s willingness to undergo surgery and tolerate potential risks should be discussed.  ### **3. Common Surgical Options**  #### **For SUI**:  - **Midurethral Sling (MUS)**: The most common and minimally invasive option.  - **Burch Colposuspension**: A non-mesh alternative for women who prefer it.  - **Urethral Bulking Agents**: A less invasive option for older or high-risk patients.  #### **For UUI**:  - **Botox Injections**: Temporary but effective for reducing bladder overactivity.  - **Sacral Neuromodulation (InterStim)**: A reversible option for refractory UUI.  - **Bladder Augmentation**: Reserved for severe cases with very small bladder capacity.  - **Urinary Diversion**: A last resort for severe, untreatable UUI.  ### **4. When to Avoid Surgery**  Surgery may not be appropriate if:  - The patient has **mild symptoms** that can be managed conservatively.  - The patient is **pregnant** or planning future pregnancies.  - The patient has **uncontrolled medical conditions** (e.g., severe heart disease, uncontrolled diabetes) that increase surgical risks.  - The patient prefers **non-surgical management**.  ### **5. Pre-Surgical Evaluation**  Before proceeding with surgery, the following steps are essential:  - **Comprehensive History and Physical Exam**: To confirm the type and severity of incontinence.  - **Bladder Diary**: To assess urinary patterns and leakage frequency.  - **Urodynamic Studies**: To evaluate bladder function and confirm the diagnosis.  - **Imaging or Cystoscopy**: If anatomical abnormalities (e.g., fistula, bladder stones) are suspected.  ### **6. Post-Surgical Expectations**  - **Recovery Time**: Varies by procedure (e.g., 1–2 weeks for sling, several weeks for more invasive surgeries).  - **Success Rates**:  - SUI surgeries (e.g., sling, Burch) have **80–90% success rates**.  - UUI surgeries (e.g., Botox, neuromodulation) have **70–80% success rates**.  - **Risks**: Include infection, urinary retention, mesh erosion (for slings), or device malfunction (for neuromodulation).  ### **Summary**  Surgery for urinary incontinence in women is recommended when:  1. Symptoms are **moderate to severe** and significantly impact quality of life.  2. **Conservative treatments** have failed.  3. The patient is a **good surgical candidate** and prefers a long-term solution.  The choice of surgery depends on the **type of incontinence**, the patient’s health, and their preferences. A **urologist** or **urogynecologist** can guide the decision-making process and recommend the most appropriate surgical option. |
| 1. **I am a 60-year-old woman who was diagnosed with mixed urinary incontinence (stress and urgency) a week ago with marked symptoms and planned surgery. Do I need urodynamic studies before surgery? If needed, what is the primary purpose of the examination?** |
| **ChatGPT-4.0:**  Urodynamic studies are often considered before surgery for urinary incontinence, especially in cases of mixed urinary incontinence (a combination of stress and urgency incontinence). However, the necessity of these studies can depend on several factors, including the severity of symptoms, previous treatments, and the specific surgical procedure being considered.  ### Purpose of Urodynamic Studies:  1. **Diagnosis Confirmation**: Urodynamic studies help confirm the diagnosis and differentiate between types of incontinence. This is crucial in mixed urinary incontinence to determine the predominant type and tailor the surgical approach accordingly.  2. **Bladder Function Assessment**: These studies provide detailed information on how well the bladder and urethra store and release urine. This includes measuring bladder pressure, capacity, and the strength of the bladder muscle (detrusor).  3. **Evaluation of Urethral Function**: They assess the function of the urethra and sphincter muscles, which is important in planning surgery for stress incontinence.  4. **Identifying Underlying Issues**: Urodynamic tests can reveal other underlying issues, such as bladder outlet obstruction or detrusor overactivity, which might influence surgical decisions.  5. **Predicting Surgical Outcomes**: The results can help predict the likelihood of success with surgery and guide the choice of surgical procedure. For instance, if urgency symptoms are predominant, additional treatments might be needed alongside surgery.  ### Considerations:  - **Clinical Guidelines**: Some clinical guidelines recommend urodynamic testing before surgery, especially in complex cases or when the diagnosis is uncertain.  - **Individualized Approach**: The decision to perform urodynamic studies should be based on an individualized approach, considering the patient's specific symptoms, medical history, and the expertise of the healthcare provider.  - **Discussion with Healthcare Provider**: It is important to have a thorough discussion with your urologist or gynecologist about the potential benefits and limitations of urodynamic studies in your specific case.  In summary, while urodynamic studies can provide valuable information and help optimize surgical outcomes, the decision to perform them should be tailored to your individual needs and circumstances. |
| **^&^DeepSeek-R1:**  chat_to_reasoning>Okay, the user is a 60-year-old woman diagnosed with mixed urinary incontinence (stress and urgency) and is planning surgery. She's asking if she needs urodynamic studies (UDS) before surgery and the primary purpose of the exam.  >  >First, I need to recall what urodynamic studies are. They're tests that assess how the bladder, sphincters, and urethra are storing and releasing urine. They measure pressure, flow, and other factors.  >  >Now, mixed incontinence involves both stress (leakage with activity) and urge (sudden need to go) components. Surgery is usually considered for stress incontinence, like slings or bladder suspension. But since she has mixed symptoms, the surgeon needs to confirm the exact cause and rule out other issues like obstruction or neurogenic bladder.  >  >Guidelines from organizations like AUA (American Urological Association) or IUGA (International Urogynecological Association) might recommend UDS before surgery for complex cases. Since her case is marked and she's planning surgery, UDS could be necessary to ensure the right surgical approach.  >  >The primary purposes would be confirming the diagnosis, assessing the severity of each component, checking for bladder dysfunction (like overactivity or poor contractility), and ruling out other issues. For example, if she has detrusor overactivity, surgery might not address the urge component, so additional treatments would be needed. Also, if there's poor bladder emptying, surgery could worsen retention.  >  >So, the answer is yes, she likely needs UDS before surgery. The main purpose is to provide detailed bladder function data to tailor the surgical plan and avoid complications. But I should also mention that in straightforward stress incontinence cases, UDS might not be needed. However, mixed symptoms and planned surgery make it more necessary. Maybe mention that her surgeon will decide based on her specific case, but generally, it's recommended for complex or unclear diagnoses.  >chat_end_reasoning  **Yes, urodynamic studies (UDS) are typically recommended before surgery for mixed urinary incontinence (UI)**. Here’s why they are important and what they aim to achieve:  ### **Why Urodynamic Studies Are Likely Needed:**  1. **Confirm the Diagnosis**  - Mixed UI involves both **stress incontinence** (leakage with physical activity) and **urge incontinence** (overactive bladder). UDS helps clarify the dominant type and rule out other conditions (e.g., bladder outlet obstruction, neurogenic bladder, or overflow incontinence) that might mimic symptoms.  2. **Evaluate Bladder Function**  - UDS measures:  - **Bladder capacity**: How much urine your bladder can hold.  - **Detrusor muscle activity**: Whether involuntary bladder contractions (overactivity) are causing urgency.  - **Urethral sphincter function**: Weakness in the urethra that contributes to stress leakage.  - **Bladder emptying efficiency**: Ensures you’re not retaining urine, which could worsen after surgery.  3. **Guide Surgical Decision-Making**  - Surgery (e.g., a **midurethral sling** for stress incontinence) may not address urge symptoms. UDS helps:  - Determine if surgery alone is sufficient or if additional treatments (e.g., medications, Botox) are needed for urge symptoms.  - Avoid complications (e.g., postoperative urinary retention or worsened urgency).  4. **Identify Contraindications**  - UDS can detect conditions like **detrusor underactivity** (weak bladder contractions) or **high-pressure bladders**, which may require alternative surgical plans or nonsurgical management.  ### **Primary Purpose of Urodynamic Testing:**  The exam aims to **objectively assess how well your bladder and urethra store and empty urine** by:  - Measuring pressures in the bladder and abdomen during filling and voiding.  - Identifying involuntary bladder contractions (indicative of urge UI).  - Testing urethral closure strength (to confirm stress UI).  - Ruling out abnormalities that could impact surgical success or safety.  ### **When UDS Might *Not* Be Required:**  - **Clear stress-dominant UI** with minimal urge symptoms.  - **Uncomplicated cases** where symptoms and physical exam strongly align with stress UI.  - However, for **mixed UI** (especially with marked symptoms), most guidelines (e.g., AUA, IUGA) recommend UDS to optimize outcomes.  ### **What to Expect During UDS:**  - A catheter is placed in the bladder to fill it with fluid while pressure sensors record activity.  - You’ll be asked to cough, strain, or delay urination to simulate daily triggers.  - The test is mildly uncomfortable but brief (30–60 minutes).  ### **Key Takeaway:**  UDS is a **critical step** to ensure surgery is appropriate and tailored to your specific bladder function. Discuss the results with your surgeon to confirm the procedure (e.g., sling, bladder repair) and whether urge symptoms need parallel management (e.g., anticholinergic drugs, pelvic floor therapy).  If your surgeon hasn’t already ordered UDS, ask for clarification. Proper preoperative testing reduces risks and improves long-term success! |
| 1. **I am a 45-year-old woman who began having frequent urination, urgency, and multiple awakenings during the night to urinate 2 months ago. What is the role of the bladder diary in assessing my condition? What key information should be recorded?** |
| **ChatGPT-4.0:**  A bladder diary is a valuable tool in assessing urinary symptoms such as frequent urination, urgency, and nocturia (waking up at night to urinate). It helps both you and your healthcare provider understand your bladder habits and identify patterns or triggers related to your symptoms. Here’s how a bladder diary can be beneficial and what key information should be recorded:  ### Role of a Bladder Diary:  1. **Symptom Assessment**: It provides a detailed record of your urinary symptoms, which helps in diagnosing the type of urinary issue you may have, such as overactive bladder or nocturia.  2. **Pattern Identification**: By tracking your urinary habits over several days, you and your healthcare provider can identify patterns, such as times of day when symptoms are worse or potential triggers for urgency.  3. **Treatment Planning**: The information gathered can guide treatment decisions, such as lifestyle modifications, behavioral therapies, or medications.  4. **Monitoring Progress**: A bladder diary can be used to monitor the effectiveness of any treatments or interventions you undergo.  ### Key Information to Record:  1. **Time and Amount of Urination**: Record the time of each urination and, if possible, measure and note the volume of urine passed. This helps assess bladder capacity and frequency.  2. **Fluid Intake**: Note the type and amount of fluids you consume, including the time of consumption. This helps correlate fluid intake with urinary patterns.  3. **Episodes of Urgency**: Document any episodes of sudden, strong urges to urinate, and note if you made it to the bathroom in time.  4. **Incontinence Episodes**: If any leakage occurs, record the time and context (e.g., during physical activity, after a strong urge).  5. **Nocturia**: Note the number of times you wake up at night to urinate and any factors that might have contributed (e.g., drinking fluids before bed).  6. **Other Symptoms**: Record any other symptoms such as pain, discomfort, or burning during urination.  7. **Activities and Triggers**: Note any activities or events that seem to trigger symptoms, such as caffeine intake, stress, or physical exertion.  ### How to Use a Bladder Diary:  - **Duration**: Keep the diary for at least 3 days, including both weekdays and weekends, to capture a comprehensive picture of your urinary habits.  - **Consistency**: Be consistent in recording the information as soon as possible after each event to ensure accuracy.  By maintaining a bladder diary, you provide your healthcare provider with essential data to better understand your condition and tailor an appropriate treatment plan. |
| **^&^DeepSeek-R1:**  chat_to_reasoning>Okay, the user is a 45-year-old woman experiencing urinary symptoms for two months. She wants to know the role of a bladder diary and what to record. Let me break this down.  >  >First, I need to recall what a bladder diary is. From previous knowledge, it's a tool used to track urination habits over a period. The main purpose is to help diagnose the type of urinary issue by providing detailed data.  >  >So, the role of the bladder diary here would be to identify patterns in her symptoms. Since she has frequency, urgency, and nocturia, the diary can show if there's a specific time of day these occur, how much she's drinking, and possible triggers. It can also help differentiate between conditions like overactive bladder, stress incontinence, or others.  >  >Next, the key information to record. Time of each void, volume (maybe she needs to measure), episodes of urgency and incontinence, fluid intake types and amounts, nighttime awakenings, and any associated symptoms like pain. Also, noting activities that might trigger symptoms, like drinking caffeine or exercising.  >  >Wait, she mentioned nocturia, so tracking how many times she wakes up at night is crucial. Also, the volume voided at night versus daytime could indicate if it's due to excess urine production at night.  >  >I should also mention that the diary is usually kept for 3-7 days. It helps the healthcare provider see patterns that aren't apparent in a single visit. For example, if her fluid intake is high in the evening, that could contribute to nocturia. Or if urgency occurs after certain drinks.  >  >Potential pitfalls: She might forget to record every instance, so advising consistency is important. Also, explaining how the diary guides further testing or treatment. Like, if the diary shows high urine output, they might check for diabetes insipidus or other causes.  >  >Is there anything else? Maybe the difference between urgency and frequency. She should note when she feels a strong urge versus just going frequently without urgency. That could help distinguish between overactive bladder and other issues.  >  >Also, including leakage episodes, even if minor, to check for stress vs urge incontinence. But she didn't mention incontinence, just urgency and frequency. Still, it's worth recording in case she has occasional leaks she didn't mention.  >  >In summary, the bladder diary's role is to collect detailed data on her urinary habits and symptoms to aid diagnosis. Key info includes timing, volume, urgency episodes, fluid intake, nocturia, and possible triggers. This helps the doctor make informed decisions about further tests or treatments.  >chat_end_reasoning  **Role of the Bladder Diary in Assessing Your Condition**  A bladder diary (also called a **voiding diary** or **frequency-volume chart**) is a critical tool for evaluating urinary symptoms like frequent urination, urgency, and nocturia (nighttime awakenings to urinate). Here’s how it helps:  1. **Identifies Patterns**  - Reveals *when* symptoms occur (e.g., daytime vs. nighttime, after specific triggers).  - Distinguishes between **overactive bladder (OAB)**, **excessive fluid intake**, or other causes.  2. **Quantifies Symptoms**  - Measures **urine volume** per void to detect abnormally small or large voids.  - Tracks the **number of urgency episodes** and **nocturnal awakenings**.  3. **Guides Diagnosis**  - Helps differentiate between **urge incontinence**, **nocturnal polyuria** (excessive nighttime urine production), or **behavioral habits** (e.g., drinking too much before bed).  - Rules out conditions like **diabetes**, **UTIs**, or **bladder dysfunction**.  4. **Informs Treatment**  - Provides data to tailor lifestyle changes (e.g., fluid restriction) or medications.  ### **Key Information to Record**  Aim to document **3–7 consecutive days**, including weekdays and weekends. Track:  1. **Time of Each Urination**  - Note every trip to the bathroom, even small leaks.  2. **Volume Voided** (if possible)  - Use a measuring cup to record urine output in milliliters (mL).  3. **Urgency Episodes**  - Rate urgency intensity (e.g., scale of 1–3: mild, moderate, severe).  4. **Incontinence Episodes**  - Note if leakage occurs with urgency, coughing, or activity.  5. **Fluid Intake**  - Type (water, coffee, alcohol), volume, and timing of all beverages.  6. **Nighttime Awakenings**  - Record how many times you wake up to urinate and the volume each time.  7. **Associated Triggers/Symptoms**  - Pain, discomfort, or activities (e.g., exercise, caffeine consumption).  ### **Example Entry**  \| Time \| Volume Voided (mL) \| Urgency (1–3) \| Leakage? \| Fluid Intake (Type/Amount) \| Notes \|  \|----------\|--------------------\|--------------\|----------\|----------------------------\|----------------------\|  \| 7:00 AM \| 300 \| 3 \| No \| Coffee (200 mL) \| Woke up with urgency \|  \| 9:30 AM \| 150 \| 2 \| Yes \| Water (100 mL) \| Leaked after sneezing\|  \| 12:00 PM \| 400 \| 1 \| No \| None \| \|  ### **Why This Matters**  - **Nocturnal Polyuria**: If nighttime urine output exceeds **20–33% of daily total**, it suggests hormonal issues (e.g., low vasopressin) or fluid retention.  - **Small Frequent Voids**: May indicate **overactive bladder** or **habitual voiding**.  - **High Caffeine/Alcohol Intake**: These irritate the bladder and worsen urgency.  Your healthcare provider will analyze the diary to determine if further tests (e.g., urinalysis, urodynamics) are needed or if lifestyle modifications (e.g., timed voiding, reducing evening fluids) can alleviate symptoms. Bring the completed diary to your appointment for a targeted evaluation! |
| 1. **I am a 70-year-old woman with severe symptoms of stress urinary incontinence and pelvic organ prolapse. Is preoperative imaging (e.g., ultrasound or MRI) necessary? If so, for what purpose?** |
| **ChatGPT-4.0:**  In cases of severe stress urinary incontinence combined with pelvic organ prolapse, preoperative imaging may be considered to provide detailed anatomical information that can aid in surgical planning. However, the necessity of imaging depends on various factors, including the severity of the symptoms, the type of prolapse, and the specific surgical procedure being considered. Here’s an overview of when preoperative imaging might be necessary and its purpose:  ### When Preoperative Imaging Might Be Necessary:  1. **Complex Cases**: Imaging is more likely to be recommended in complex cases where the anatomy is unclear or when multiple pelvic organs are involved in the prolapse.  2. **Surgical Planning**: If the surgeon requires more detailed information about the pelvic anatomy to plan the surgical approach effectively, imaging can be useful.  3. **Uncertain Diagnosis**: If there is uncertainty about the extent or type of prolapse, imaging can help clarify the diagnosis.  4. **Previous Surgeries**: In cases where the patient has had previous pelvic surgeries, imaging might be used to assess any changes or complications in the pelvic anatomy.  ### Purpose of Preoperative Imaging:  1. **Anatomical Assessment**: Imaging provides a detailed view of the pelvic organs, including the bladder, uterus, rectum, and supporting structures, helping to assess the extent and type of prolapse.  2. **Identifying Other Conditions**: It can help identify any other pelvic conditions that might affect surgical planning, such as fibroids or masses.  3. **Surgical Planning**: Imaging helps the surgeon plan the most appropriate surgical approach, especially in cases where multiple organs are prolapsed or when reconstructive surgery is needed.  4. **Assessing Pelvic Floor Muscles**: MRI, in particular, can provide detailed images of the pelvic floor muscles and connective tissues, which can be important in planning surgery for both prolapse and incontinence.  ### Types of Imaging:  - **Ultrasound**: Can be used to assess the bladder and other pelvic structures. It is less commonly used for detailed assessment of prolapse but can provide useful information in certain cases.  - **MRI**: Offers a comprehensive view of the pelvic anatomy and is particularly useful for complex cases or when detailed information about the pelvic floor muscles and connective tissues is needed.  Ultimately, the decision to use preoperative imaging should be made in consultation with your healthcare provider, taking into account your specific symptoms, medical history, and the planned surgical procedure. The goal is to ensure that the surgery is as effective and safe as possible. |
| **^&^DeepSeek-R1:**  chat_to_reasoning>Okay, the user is a 70-year-old woman with severe stress urinary incontinence (SUI) and pelvic organ prolapse (POP) planning for surgery. She's asking if preoperative imaging like ultrasound or MRI is necessary and why. Let me break this down.  >  >First, I need to recall the standard preoperative assessments for SUI and POP. Typically, physical exams and urodynamic studies (UDS) are common. But imaging? Maybe in certain cases. Let's think about guidelines. The AUA and other societies might have recommendations here.  >  >Stress urinary incontinence often requires a physical exam, maybe a cough stress test, and UDS to confirm the diagnosis and rule out other issues like detrusor overactivity. Pelvic organ prolapse assessment usually involves the POP-Q system during physical exam. But when does imaging come into play?  >  >Possible reasons for imaging: If the prolapse is complex or there's uncertainty about the extent, especially if it's a recurrent case. Also, if there's suspicion of other pelvic floor abnormalities, like levator ani defects or enterocele. MRI can show soft tissue details better, while ultrasound is dynamic and can be done in real-time during straining.  >  >Another angle: Preoperative imaging might help in surgical planning. For example, identifying which compartments are involved (anterior, posterior, apical) and the severity. If the prolapse is stage III or IV, imaging could help visualize the support structures. Also, in cases where neurogenic bladder is suspected, MRI might look for neurological causes.  >  >Wait, the user is 70, so maybe atrophic tissues or previous surgeries? Recurrent prolapse might need imaging to assess the integrity of pelvic muscles. Also, if there's any suspicion of an enterocele or rectocele that's not clearly identifiable on exam.  >  >But is imaging routinely recommended? From what I remember, routine imaging isn't standard unless there's a specific indication. But in severe or complex cases, yes. The purpose would be to delineate anatomy, assess muscle and connective tissue, and possibly identify coexisting issues that might affect surgical approach or choice of procedure.  >  >Also, if the patient has symptoms that don't match the physical exam findings, imaging could resolve discrepancies. For example, if she complains of bulge symptoms but exam shows minimal prolapse, maybe an MRI would show something else.  >  >So, the answer would be that preoperative imaging isn't always necessary but is indicated in certain cases. The purposes include assessing pelvic floor anatomy in detail, identifying muscle or connective tissue defects, evaluating for coexisting pelvic pathology, and aiding in surgical planning, especially for complex or recurrent cases. Ultrasound (dynamic) can assess real-time organ movement, while MRI provides detailed soft tissue images. The choice depends on the clinical scenario and available resources.  >  >I should also mention that physical exam remains primary, and imaging complements it when there's diagnostic uncertainty or complex anatomy. The main purposes are to enhance understanding of the pelvic floor structure, identify contributing factors, and plan the most appropriate surgical intervention to optimize outcomes and reduce recurrence risk.  >chat_end_reasoning  **Preoperative imaging (e.g., ultrasound or MRI)** may be recommended in your case, depending on the complexity of your pelvic organ prolapse (POP) and stress urinary incontinence (SUI). Here’s how imaging can play a role and its key purposes:  ### **When Is Preoperative Imaging Necessary?**  1. **Complex or Recurrent Pelvic Organ Prolapse**  - If your prolapse is severe (Stage III/IV), involves multiple compartments (anterior, posterior, apical), or has recurred after prior surgery, imaging helps:  - Map the exact anatomy of the prolapse.  - Identify defects in pelvic floor muscles (e.g., levator ani avulsion) or connective tissue.  - Detect **enterocele** (small bowel herniation) or **rectocele** (rectal bulging) that may not be fully apparent on physical exam.  2. **Suspected Coexisting Conditions**  - If there are concerns about:  - **Urethral diverticulum** or **bladder abnormalities** (e.g., diverticula, stones).  - **Neurogenic bladder** (e.g., from spinal cord issues), which may require MRI to assess nerve or spinal involvement.  3. **Discrepancy Between Symptoms and Exam Findings**  - If your symptoms (e.g., severe bulge, urinary retention) don’t align with the physical exam, imaging clarifies the cause.  4. **Surgical Planning for Combined Procedures**  - Imaging helps plan repairs (e.g., sacrocolpopexy, sling placement) by:  - Evaluating pelvic organ mobility.  - Assessing urethral sphincter function (via **transperineal ultrasound**).  - Guiding decisions on mesh use or alternative approaches.  ### **Primary Purposes of Imaging**  1. **Pelvic Floor Anatomy Assessment**  - **MRI**: Provides detailed 3D views of soft tissues (muscles, ligaments, organs) and identifies defects in the levator ani or endopelvic fascia.  - **Dynamic Ultrasound**: Shows real-time organ movement during straining, coughing, or voiding to assess prolapse severity and urethral hypermobility.  2. **Rule Out Hidden Pathology**  - Detect occult urinary retention, diverticula, or tumors that could impact surgical outcomes.  3. **Quantify Urethral Function**  - **Urethral sphincter imaging** (ultrasound/MRI) evaluates sphincter integrity in severe SUI, especially if intrinsic sphincter deficiency (ISD) is suspected.  4. **Predict Surgical Risks**  - Imaging may reveal factors that increase recurrence risk (e.g., weak levator muscles) or complications (e.g., bladder neck obstruction).  ### **When Imaging May *Not* Be Routinely Required**  - **Uncomplicated POP/SUI**: Physical exam (e.g., POP-Q staging, cough stress test) and urodynamic studies (UDS) often suffice.  - **Clear Surgical Plan**: If exam findings align with symptoms and standard repairs (e.g., midurethral sling, vaginal pessary trial) are planned.  ### **What to Expect**  - **MRI Pelvimetry**: Non-invasive, no radiation. You may be asked to strain during the scan to mimic prolapse.  - **Ultrasound**: Transperineal or transvaginal probes assess bladder neck mobility and prolapse dynamics.  ### **Key Takeaway**  While imaging isn’t mandatory for all POP/SUI cases, it is valuable for **severe, complex, or recurrent presentations** to optimize surgical planning and reduce risks. Discuss with your urogynecologist whether imaging is warranted based on your specific anatomy and symptoms. Combined with a physical exam and UDS, it ensures a tailored approach to restore pelvic floor function and improve quality of life. |
| 1. **I am a 55-year-old woman with a diagnosis of new-onset urinary incontinence with weight loss and hematuria. What potentially serious conditions need to be ruled out? What tests should be performed?** |
| **ChatGPT-4.0:**  In a 55-year-old woman presenting with new-onset urinary incontinence, weight loss, and hematuria (blood in the urine), it is important to consider and rule out potentially serious underlying conditions. These symptoms can be indicative of several serious conditions, including:  ### Potentially Serious Conditions:  1. **Urinary Tract Malignancies**:  - **Bladder Cancer**: Hematuria and urinary changes can be signs of bladder cancer, especially in the presence of weight loss.  - **Kidney Cancer**: Hematuria can also be a symptom of kidney cancer.  2. **Other Malignancies**:  - **Gynecological Cancers**: Such as cervical or uterine cancer, which can affect urinary function.  - **Metastatic Disease**: Cancers from other parts of the body that have spread to the urinary tract or pelvic region.  3. **Severe Infections**:  - **Urinary Tract Infection (UTI)**: While common, a severe or complicated UTI could present with these symptoms, especially if untreated or recurrent.  - **Kidney Infection (Pyelonephritis)**: This could also cause hematuria and systemic symptoms like weight loss.  4. **Other Conditions**:  - **Renal Calculi (Kidney Stones)**: Can cause hematuria and occasionally lead to secondary infections or weight loss due to pain and dietary changes.  - **Interstitial Cystitis**: Though less common, it can cause urinary symptoms and hematuria.  ### Recommended Tests:  1. **Urinalysis**: To detect blood, bacteria, or other abnormalities in the urine. It can help identify infections or other urinary abnormalities.  2. **Urine Culture**: To rule out or confirm a urinary tract infection.  3. **Cytology**: Examination of urine for abnormal cells that could indicate cancer.  4. **Imaging Studies**:  - **Ultrasound**: Initial imaging to assess the kidneys, bladder, and other pelvic structures.  - **CT Urogram**: A more detailed imaging study to evaluate the urinary tract for tumors, stones, or other abnormalities.  - **MRI**: May be used for further evaluation if needed, particularly to assess surrounding tissues.  5. **Cystoscopy**: A procedure where a camera is inserted into the bladder via the urethra to directly visualize the bladder and urethra. It's essential for evaluating potential bladder cancer.  6. **Blood Tests**:  - **Complete Blood Count (CBC)**: To check for signs of infection, anemia, or other blood-related issues.  - **Metabolic Panel**: To assess kidney function and other systemic health indicators.  7. **Pelvic Examination**: To assess for any gynecological issues that might contribute to the symptoms.  Given the combination of symptoms, it is crucial to consult with a healthcare provider promptly for a thorough evaluation to determine the underlying cause and initiate appropriate treatment. |
| **^&^DeepSeek-R1:**  chat_to_reasoning>Okay, let's tackle this question. The user is a 55-year-old woman with new-onset urinary incontinence, weight loss, and hematuria. I need to figure out what serious conditions to rule out and the necessary tests.  >  >First, the red flags here are weight loss and hematuria. These symptoms suggest something more serious than typical stress or urge incontinence. Weight loss is a systemic symptom that can be associated with malignancies, chronic infections, or other systemic diseases. Hematuria, especially in someone without a history of UTIs or stones, raises concerns about bladder or kidney cancer.  >  >So, possible serious conditions:  >  >1. **Bladder Cancer**: Hematuria is a hallmark, and it's painless usually. But since she has incontinence, maybe the tumor is irritating the bladder.  >  >2. **Kidney Cancer**: Hematuria can occur, and weight loss is a systemic symptom. Renal cell carcinoma might present with these.  >  >3. **Urothelial Carcinoma**: Affecting the urinary tract lining, could explain hematuria.  >  >4. **Gynecologic Cancers**: Cervical or uterine cancer invading the bladder could cause hematuria and incontinence. Weight loss might be a sign of advanced disease.  >  >5. **Chronic Infection**: Like TB or chronic pyelonephritis, but TB would have other symptoms like night sweats. Maybe less likely with weight loss?  >  >6. **Autoimmune Diseases**: Like interstitial cystitis, but usually without weight loss. Maybe lupus nephritis? But hematuria in that case is more glomerular.  >  >7. **Neurological Disorders**: If incontinence is neurogenic, but with weight loss and hematuria, probably not the primary concern here.  >  >Tests needed:  >  >- **Urinalysis and Urine Cytology**: Check for RBCs, WBCs, bacteria, and malignant cells. Cytology can detect cancerous cells.  >  >- **Cystoscopy**: Direct visualization of the bladder to look for tumors or lesions. Essential given hematuria.  >  >- **Imaging**: CT urogram to evaluate kidneys, ureters, bladder. Can detect masses, stones.  >  >- **Blood Tests**: CBC (anemia?), metabolic panel, markers like ESR/CRP for inflammation. Maybe tumor markers, though not specific.  >  >- **Pelvic Exam/Pap Smear**: To check for gynecologic malignancies.  >  >- **Biopsy**: If a lesion is found, biopsy to confirm malignancy.  >  >- **Urodynamic Studies**: Maybe, but given the other symptoms, probably after ruling out serious conditions.  >  >Need to make sure not to miss cancer. Also, weight loss could be due to paraneoplastic syndrome or metastasis. Hematuria in this age group is cancer until proven otherwise.  >  >Other possibilities: Maybe a stone causing hematuria and irritation leading to incontinence. But weight loss is unexplained. Infection could be a UTI, but again, weight loss is a concern. Maybe diabetes? Unlikely with these symptoms.  >  >So, the key is to prioritize ruling out malignancies and serious systemic conditions. Tests should be thorough to cover all bases.  >chat_end_reasoning  In a 55-year-old woman presenting with **new-onset urinary incontinence, weight loss, and hematuria**, several serious conditions must be prioritized for evaluation. Here’s a structured approach:  ### **Potentially Serious Conditions to Rule Out**  1. **Malignancies**  - **Bladder Cancer**: Painless hematuria is a hallmark; tumors near the bladder neck/urethra may cause incontinence.  - **Kidney Cancer** (e.g., renal cell carcinoma): Hematuria + systemic symptoms (weight loss, fatigue).  - **Urothelial Carcinoma**: Affects the urinary tract lining (kidneys, ureters, bladder).  - **Gynecologic Cancers** (e.g., cervical, uterine, ovarian): Advanced tumors may invade the bladder or ureters.  2. **Chronic/Invasive Infections**  - **Tuberculosis (TB)**: Can cause sterile pyuria, hematuria, and constitutional symptoms.  - **Chronic Pyelonephritis**: May lead to renal scarring and hematuria.  3. **Autoimmune/Inflammatory Disorders**  - **Interstitial Cystitis/Bladder Pain Syndrome**: Rarely causes hematuria or weight loss.  - **Vasculitis** (e.g., lupus nephritis, granulomatosis with polyangiitis): Glomerular hematuria + systemic inflammation.  4. **Other Systemic Conditions**  - **Diabetes**: Unlikely to explain hematuria but can cause incontinence and weight loss (if poorly controlled).  - **Neurologic Disorders** (e.g., spinal cord compression, multiple sclerosis): Rarely present with isolated hematuria.  ### **Essential Diagnostic Tests**  1. **Urinalysis with Microscopy**  - Confirm hematuria (RBCs), rule out infection (WBCs, nitrites), and assess for dysmorphic RBCs (suggesting glomerular disease).  2. **Urine Cytology**  - Detect malignant cells (sensitivity increases with high-grade bladder cancer).  3. **Cystoscopy**  - **Gold standard** to visualize bladder mucosa, identify tumors, or rule out urethral/bladder lesions.  4. **Imaging**  - **CT Urogram**: Evaluates kidneys, ureters, and bladder for masses, stones, or structural abnormalities.  - **Pelvic MRI/Ultrasound**: Assess for gynecologic malignancies or pelvic organ involvement.  5. **Blood Tests**  - **CBC**: Check for anemia (chronic blood loss or malignancy).  - **Basic Metabolic Panel**: Assess renal function (e.g., elevated creatinine).  - **Inflammatory Markers** (ESR/CRP): Elevated in chronic infections or autoimmune disease.  - **HbA1c**: Rule out diabetes.  6. **Gynecologic Evaluation**  - **Pelvic Exam + Pap Smear**: Exclude cervical/uterine masses.  - **Transvaginal Ultrasound**: Evaluate ovaries/uterus.  7. **Biopsy**  - If a bladder/renal mass or suspicious lesion is identified.  8. **Optional Tests**  - **Urodynamic Studies**: If incontinence persists after ruling out malignancy (assess bladder function).  - **Quantitative Urine Culture**: Rule out atypical infections (e.g., TB).  ### **Key Considerations**  - **Hematuria in this age group is cancer until proven otherwise**—prompt evaluation is critical.  - **Weight loss** raises concern for advanced/metastatic disease or systemic inflammation.  - **Urinary incontinence** may result from bladder outlet obstruction (e.g., tumor) or irritation (e.g., infection/malignancy).  ### **Next Steps**  1. Refer urgently to a **urologist** for cystoscopy and imaging.  2. Collaborate with a **gynecologist** if pelvic pathology is suspected.  3. Address reversible causes (e.g., infection) while awaiting results.  Early diagnosis of malignancies or autoimmune conditions significantly impacts prognosis. Ensure close follow-up to monitor for progression or recurrence. |
| 1. **I am a 40-year-old woman with a diagnosis of mild stress urinary incontinence and would prefer nonsurgical management. What are the recommended first-line nonsurgical treatments? How to develop an individualized treatment plan?** |
| **ChatGPT-4.0:**  For a 40-year-old woman with mild stress urinary incontinence (SUI) who prefers nonsurgical management, there are several first-line treatment options that can be effective. Developing an individualized treatment plan involves considering your specific symptoms, lifestyle, and preferences. Here are the recommended first-line nonsurgical treatments and steps to create a personalized plan:  ### First-Line Nonsurgical Treatments:  1. **Pelvic Floor Muscle Training (PFMT):**  - **Kegel Exercises**: These exercises strengthen the pelvic floor muscles, which support the bladder and urethra. Consistent practice can improve symptoms of SUI.  - **Guidance from a Physical Therapist**: A specialized pelvic floor physical therapist can provide instruction and ensure exercises are done correctly.  2. **Lifestyle Modifications:**  - **Weight Management**: If overweight, losing weight can reduce pressure on the bladder and improve symptoms.  - **Bladder Training**: Scheduled voiding and gradually increasing the time between bathroom visits can help improve bladder control.  - **Fluid Management**: Monitoring fluid intake and reducing consumption of bladder irritants (e.g., caffeine, alcohol) can help.  3. **Behavioral Therapies:**  - **Biofeedback**: This technique uses sensors to provide feedback on pelvic floor muscle activity, helping to improve exercise effectiveness.  - **Electrical Stimulation**: Low-grade electrical impulses can help strengthen pelvic floor muscles.  4. **Pessary Use:**  - **Vaginal Pessary**: A device inserted into the vagina to support the bladder and reduce leakage. It can be a good option for some women, especially those with mild prolapse.  5. **Continence Products:**  - **Absorbent Pads or Liners**: These can provide protection and confidence while other treatments take effect.  ### Developing an Individualized Treatment Plan:  1. **Assessment by a Healthcare Provider:**  - Start with a comprehensive evaluation by a healthcare provider to confirm the diagnosis and rule out other conditions.  2. **Setting Goals:**  - Identify specific goals for treatment, such as reducing the frequency of leakage or improving quality of life.  3. **Choosing Appropriate Interventions:**  - Based on the assessment, your healthcare provider can recommend the most suitable interventions. This might include starting with pelvic floor exercises and lifestyle changes, with the option to add other treatments as needed.  4. **Regular Monitoring and Adjustments:**  - Regular follow-up appointments to assess progress and make necessary adjustments to the treatment plan.  5. **Patient Education and Support:**  - Understanding the condition and the rationale behind each treatment option can empower you to manage symptoms effectively.  6. **Consideration of Personal Preferences:**  - Your comfort and preferences should guide the choice of treatments, ensuring that the plan is sustainable and acceptable for you.  By working closely with a healthcare provider, you can develop a tailored plan that addresses your specific needs and improves your quality of life while managing stress urinary incontinence. |
| **^&^DeepSeek-R1:**  chat_to_reasoning>Okay, let's tackle this query. The user is a 40-year-old woman with mild stress urinary incontinence (SUI) who prefers nonsurgical management. I need to outline the first-line nonsurgical treatments and how to create an individualized plan.  >  >First, I should recall the standard nonsurgical options for SUI. Pelvic floor muscle training (PFMT) is definitely a key recommendation. Then there's lifestyle modifications like weight loss and fluid management. Maybe bladder training as well. Oh, and vaginal pessaries could be an option. Also, newer things like extracorporeal magnetic innervation (ExMI) or electrical stimulation might come into play.  >  >Wait, she mentioned mild SUI, so the treatments should be appropriate for mild cases. First-line would typically start with PFMT and lifestyle changes. I should explain each of these in detail. For PFMT, it's important to mention proper technique and supervision by a physiotherapist. Lifestyle changes like weight loss, smoking cessation, reducing caffeine and alcohol. Bladder training might help if there's a mixed component, but since it's SUI, maybe less so. Pessaries can be tried if she's open to using a device.  >  >Now, developing an individualized plan. Factors like her BMI, activity level, occupation, and personal preferences matter. For example, if she's active or does high-impact exercises, modifying activities could help. Also, her motivation to adhere to PFMT is crucial. Maybe assess her commitment level and provide structured programs. If she prefers non-invasive options, start with PFMT and lifestyle. If she's interested in technology, ExMI or apps for biofeedback could be suggested. Follow-up is important to adjust the plan based on progress.  >  >I should also mention combining treatments for better outcomes. Like PFMT plus lifestyle changes. Also, set realistic expectations. Maybe include a stepwise approach, escalating treatments if first-line isn't sufficient. But since she prefers nonsurgical, focus on those options. Avoid mentioning surgical unless necessary, but note when referral might be needed if symptoms worsen.  >  >Need to check if there are any contraindications or considerations for these treatments. For instance, pessaries require fitting and comfort. Electrical stimulation is safe but might not be for everyone. Also, emphasize patient education and support groups. Maybe provide a sample plan structure.  >  >Make sure the answer is organized, clear, and addresses both parts of the question: recommended treatments and individualizing the plan. Use headings for clarity, but avoid markdown. Keep it conversational but professional.  >chat_end_reasoning  For a 40-year-old woman with **mild stress urinary incontinence (SUI)** seeking nonsurgical management, the following **first-line treatments** and strategies for an **individualized plan** are recommended:  ### **First-Line Nonsurgical Treatments**  1. **Pelvic Floor Muscle Training (PFMT/Kegels)**  - **What**: Strengthening the pelvic floor muscles to improve urethral support.  - **How**:  - Perform 3 sets of 8–12 slow, sustained contractions daily (hold 6–8 seconds).  - Incorporate quick contractions during activities that trigger leakage (e.g., coughing).  - Use **biofeedback** or **mobile apps** (e.g., Squeezy, Elvie) to ensure proper technique.  - **Evidence**: Reduces SUI episodes by 50–80% in motivated patients.  2. **Lifestyle Modifications**  - **Weight Management**: Even a 5–10% reduction in BMI (if overweight) can improve symptoms.  - **Smoking Cessation**: Chronic coughing worsens SUI.  - **Fluid/Bladder Irritant Management**:  - Limit caffeine, alcohol, and carbonated drinks.  - Avoid excessive fluid intake (>2–3 L/day).  3. **Bladder Training**  - **Purpose**: Improve bladder capacity and control.  - **Method**: Gradually increase voiding intervals by 15–30 minutes weekly, aiming for 3–4 hour intervals.  4. **Vaginal Pessary**  - **What**: A removable silicone device inserted into the vagina to support the urethra.  - **Best For**: Women who want immediate symptom relief or cannot commit to PFMT.  - **Types**: Ring pessary (most common) or incontinence-specific designs (e.g., Uresta).  5. **Topical Estrogen (if postmenopausal or atrophic vaginitis)**  - **Use**: Low-dose vaginal estrogen cream/tablets to improve urethral mucosal health (if vaginal atrophy is present).  6. **Newer Options (if accessible)**  - **Extracorporeal Magnetic Innervation (ExMI)**: Noninvasive magnetic stimulation to strengthen pelvic muscles.  - **Electrical Stimulation**: Mild electrical pulses via vaginal probe to enhance muscle recruitment.  ### **Developing an Individualized Treatment Plan**  1. **Assess Patient-Specific Factors**  - **Goals**: Does she prioritize convenience (e.g., pessary) vs. active participation (PFMT)?  - **Lifestyle**: Occupation, exercise habits (e.g., high-impact sports), and ability to perform daily PFMT.  - **Anatomic Factors**: Pelvic floor tone (assessed via exam), vaginal anatomy (for pessary fitting).  2. **Combine Therapies for Synergy**  - Example: PFMT + weight loss + caffeine reduction.  - Pessary + PFMT for immediate relief while building muscle strength.  3. **Structured Follow-Up**  - **At 3 Months**: Reassess symptoms (e.g., 24-hour pad test, bladder diary).  - Adjust plan if progress stalls (e.g., add biofeedback, escalate pessary size).  4. **Address Barriers to Adherence**  - **Time Constraints**: Shorten PFMT sessions to 5 minutes, 3x/day.  - **Motivation**: Use app-based reminders or group therapy for accountability.  5. **Educate on Realistic Expectations**  - Mild SUI often improves significantly with consistent PFMT/lifestyle changes.  - Pessaries require regular cleaning/refitting (every 3–6 months).  ### **When to Refer for Advanced Options**  - If symptoms worsen despite 6 months of adherence.  - If mixed incontinence (urgency) develops, consider adding anticholinergics (e.g., mirabegron).  - For persistent leaks during high-impact activities, consider a **urethral bulking agent** (minimally invasive).  ### **Sample Individualized Plan**  - **Week 1–4**:  - PFMT instruction with a pelvic physiotherapist.  - Bladder diary + caffeine reduction.  - **Week 4–12**:  - Add biofeedback-guided PFMT.  - Introduce pessary trial if leakage persists during exercise.  - **3-Month Follow-Up**:  - Re-evaluate symptoms; consider ExMI or electrical stimulation if progress stalls.  Nonsurgical management is highly effective for mild SUI when tailored to the patient’s lifestyle and preferences. Consistency and patient engagement are key to success! |
| 1. **I am a 35-year-old woman with mild postpartum stress urinary incontinence and am planning to try pelvic floor muscle training. Please guide me to effective pelvic floor muscle training? How are training cycles and follow-up frequencies determined?** |
| **ChatGPT-4.0:**  Pelvic Floor Muscle Training (PFMT), commonly known as Kegel exercises, is an effective method for managing mild postpartum stress urinary incontinence. Here's a guide on how to perform these exercises effectively, along with information on training cycles and follow-up frequencies:  ### Performing Pelvic Floor Muscle Training:  1. **Identify the Right Muscles:**  - To locate your pelvic floor muscles, try to stop urination midstream. The muscles you use are your pelvic floor muscles. However, do not make a habit of stopping urination as it can lead to incomplete emptying of the bladder.  2. **Correct Technique:**  - **Contract**: Tighten your pelvic floor muscles and hold the contraction for about 3 to 5 seconds.  - **Relax**: Release the contraction and relax for 3 to 5 seconds.  - Ensure you are not squeezing your abdomen, thighs, or buttocks. Breathing should remain normal.  3. **Repetitions and Sets:**  - Aim for at least 10 repetitions per set.  - Perform 3 sets per day.  4. **Gradual Progression:**  - As your muscles get stronger, gradually increase the hold time to 10 seconds and the relaxation time to 10 seconds.  5. **Consistency is Key:**  - Consistency is essential for effectiveness. Try to integrate these exercises into your daily routine, such as while brushing your teeth or watching TV.  ### Training Cycles and Follow-Up:  1. **Initial Training Period:**  - Start with a 6 to 12-week training period to allow time for muscle strengthening and improvement in symptoms.  2. **Regular Monitoring:**  - Track your progress by noting any changes in symptoms, such as reduced leakage or improved control.  3. **Follow-Up Frequency:**  - **Initial Follow-Up**: After the first 4 to 6 weeks, schedule a follow-up with a healthcare provider or a pelvic floor physical therapist to assess technique and progress.  - **Subsequent Follow-Ups**: Depending on progress, follow-ups may be scheduled every 3 months or as needed.  4. **Long-Term Maintenance:**  - Once improvement is noted, continue with a maintenance routine of exercises. This might mean doing them less frequently, such as once a day or a few times a week, as advised by your healthcare provider.  5. **Professional Guidance:**  - Consider consulting a pelvic floor physical therapist for personalized guidance and to ensure proper technique. They can also provide biofeedback or other adjunctive therapies if needed.  By consistently practicing pelvic floor muscle training and following up with healthcare professionals, you can effectively manage postpartum stress urinary incontinence and improve your pelvic health. |
| **^&^DeepSeek-R1:**  chat_to_reasoning>Okay, the user is a 35-year-old woman with mild postpartum stress urinary incontinence (SUI) planning to try pelvic floor muscle training (PFMT). I need to guide her effectively. Let me start by recalling the key components of PFMT for postpartum SUI.  >  >First, postpartum SUI is common due to weakened pelvic floor muscles from pregnancy and childbirth. PFMT is the first-line treatment. I should explain how to perform Kegel exercises correctly, as improper technique is a common issue. Emphasize finding the right muscles, maybe by stopping urine flow midstream, but caution against doing this regularly to avoid urinary retention.  >  >Next, structure the training program. It should include both slow and fast contractions. Slow contractions build endurance, while fast ones help with quick responses during activities that cause leakage, like coughing. Suggest a routine: maybe 3 sets of 8-12 slow contractions daily, holding each for 6-8 seconds, plus quick contractions. Mention progression over weeks to increase intensity.  >  >Training cycles and follow-up frequency are important. Typically, studies suggest at least 3 months of consistent training. Follow-up every 4-6 weeks to assess progress and adjust the plan. If using apps or biofeedback, that can help with adherence and proper technique.  >  >Individualizing the plan is crucial. Consider factors like delivery method (vaginal vs. C-section), any perineal trauma, current physical activity level. Encourage integrating PFMT into daily routines, maybe linking it to habitual activities like brushing teeth.  >  >Also, address common barriers: forgetting to do exercises, difficulty identifying muscles. Suggest reminders, apps, or working with a pelvic floor physiotherapist. Mention lifestyle modifications like managing fluid intake, avoiding constipation, and weight management if needed.  >  >Need to set realistic expectations. Improvement can take weeks to months. If no progress after 12 weeks, refer to a specialist. Also, mention when to seek help, like if symptoms worsen or if there's pain.  >  >Avoid medical jargon, keep it encouraging and practical. Make sure the advice is evidence-based and aligns with clinical guidelines. Check if there's any recent literature on PFMT protocols for postpartum SUI to ensure recommendations are up-to-date.  >  >Wait, did the previous answer cover similar points? Let me check. The last response was about a 40-year-old with SUI, so some overlap, but this is postpartum specific. Need to tailor advice to postpartum recovery, possibly addressing diastasis recti or other postpartum factors.  >  >Include tips on posture and core engagement, as core stability supports pelvic floor function. Maybe advise against high-impact exercises until strength improves.  >  >Also, consider the emotional aspect. Postpartum women might feel overwhelmed, so emphasize the importance of self-care and gradual progress. Reassure that mild SUI is common and treatable.  >  >Alright, structure the answer with clear sections: Correct Technique, Training Protocol, Progression, Follow-Up, Individualization, and Additional Tips. Make sure each part is concise and actionable.  >chat_end_reasoning  For a 35-year-old woman with **mild postpartum stress urinary incontinence (SUI)**, pelvic floor muscle training (PFMT) is an excellent first-line approach. Here’s a structured, evidence-based guide to effective PFMT, including training cycles and follow-up strategies:  ### **Step 1: Learn Proper Technique**  **Goal**: Correctly isolate and engage the pelvic floor muscles (PFMs).  **How to**:  1. **Identify the muscles**:  - Pretend to stop urine flow midstream (only do this once to avoid urinary retention).  - Tighten the muscles around the vagina and anus (avoid contracting thighs, glutes, or abdomen).  2. **Use visual cues**: Imagine lifting the pelvic floor upward and inward.  3. **Avoid bearing down**: Ensure you’re *contracting*, not straining.  **Tip**: A pelvic floor physiotherapist can provide real-time feedback via internal/external palpation or biofeedback devices.  ### **Step 2: Training Protocol**  **Daily Routine**:  - **Slow Contractions** (for endurance):  - 8–12 slow squeezes, holding each for **6–8 seconds**, followed by **6–8 seconds of rest**.  - Repeat 3 times daily (e.g., morning, afternoon, evening).  - **Quick Contractions** (for rapid response during coughing/sneezing):  - 5–10 rapid squeezes (1–2 seconds each), with 2–4 seconds rest.  - Perform 1–2 sets daily.  **Weekly Progression**:  - Increase hold time by 1–2 seconds weekly (max 10 seconds).  - Add 1–2 contractions per set every 2 weeks (max 15–20 reps/set).  **Sample Schedule**:  - **Weeks 1–2**: 3 sets of 8 slow contractions (6-sec hold) + 1 set of 5 quick contractions.  - **Weeks 3–4**: 3 sets of 10 slow contractions (8-sec hold) + 2 sets of 8 quick contractions.  ### **Step 3: Integrate Functional Training**  - **“The Knack”**: Contract PFMs *before* activities that trigger leaks (e.g., coughing, lifting your baby).  - **Posture**: Practice PFMT in different positions (lying, sitting, standing) to mimic real-life scenarios.  ### **Step 4: Determine Training Cycles & Follow-Up**  1. **Initial Phase (Weeks 1–12)**:  - **Frequency**: 3–5 days/week (daily is ideal).  - **Follow-Up**:  - **At 4–6 weeks**: Assess technique and progress via a physiotherapist or self-report (e.g., bladder diary, fewer leaks).  - **At 12 weeks**: Re-evaluate symptoms. Many women see improvement by 3 months.  2. **Maintenance Phase (After 12 weeks)**:  - Reduce to 2–3 sessions/week if symptoms resolve.  - Continue “The Knack” during high-risk activities.  ### **Step 5: Individualize Your Plan**  - **If you had a traumatic delivery** (e.g., forceps, severe tearing):  - Start with shorter holds (3–4 seconds) and progress slower.  - Consider biofeedback to ensure proper muscle recruitment.  - **If you’re breastfeeding**:  - Low estrogen levels may reduce pelvic floor tone; combine PFMT with topical vaginal estrogen (if approved by your doctor).  - **If you’re returning to exercise**:  - Avoid high-impact workouts (e.g., running) until PFM strength improves.  - Use a postpartum support belt during exercise to reduce intra-abdominal pressure.  ### **Step 6: Track Progress**  - **Bladder Diary**: Record leaks, triggers, and voiding patterns.  - **Apps**: Try **Squeezy**, **Perifit**, or **Elvie** for guided routines and reminders.  - **Self-Test**:  - Can you hold a contraction for 10 seconds without straining?  - Do leaks decrease during coughing/laughing?  ### **When to Seek Help**  - If symptoms persist after **12 weeks** of consistent PFMT.  - If you develop pain, urgency, or worsening leaks (may indicate mixed incontinence or pelvic organ prolapse).  ### **Additional Tips**  - **Avoid Constipation**: Straining weakens PFMs. Increase fiber/water intake.  - **Modify Lifting Techniques**: Lift your baby using legs (not back) while engaging your PFMs.  PFMT is highly effective for postpartum SUI when done consistently and correctly. Many women see significant improvement within 3–6 months. If progress stalls, consult a pelvic floor physiotherapist for advanced strategies like weighted vaginal cones or electrical stimulation. You’ve got this! |
| 1. **I am a 65-year-old woman with urgency urinary incontinence, with frequent and urgent urination. What are the recommended first-line medical therapies? What are the side effects of these drugs?** |
| **ChatGPT-4.0:**  For a 65-year-old woman experiencing urgency urinary incontinence, first-line medical therapies typically include antimuscarinic agents and beta-3 adrenergic agonists. These medications help manage symptoms by reducing bladder overactivity and improving bladder control. Here’s an overview of these medications and their potential side effects:  ### First-Line Medical Therapies:  1. **Antimuscarinic Agents:**  - These medications work by blocking the action of acetylcholine, a neurotransmitter that stimulates bladder contractions.  - **Common Antimuscarinic Drugs:**  - Oxybutynin (Ditropan)  - Tolterodine (Detrol)  - Solifenacin (Vesicare)  - Darifenacin (Enablex)  - Fesoterodine (Toviaz)  - Trospium (Sanctura)  - **Potential Side Effects:**  - Dry mouth  - Constipation  - Blurred vision  - Drowsiness  - Dizziness  - Cognitive effects, particularly in older adults (e.g., confusion, memory issues)  2. **Beta-3 Adrenergic Agonists:**  - These medications activate beta-3 adrenergic receptors in the bladder, leading to relaxation of the bladder muscle and increased storage capacity.  - **Common Beta-3 Adrenergic Agonist:**  - Mirabegron (Myrbetriq)  - **Potential Side Effects:**  - Increased blood pressure  - Nasal congestion  - Headache  - Urinary tract infections  - Dizziness  ### Considerations for Treatment:  - **Individualized Approach:**  - The choice of medication should be based on your overall health, existing medical conditions, and any other medications you are taking. Discuss with your healthcare provider to determine the most appropriate option for you.  - **Monitoring and Adjustments:**  - Regular follow-up appointments are essential to monitor the effectiveness of the medication and to make any necessary adjustments. This can include changing the dosage or trying a different medication if side effects are bothersome or if the initial medication is not effective.  - **Non-Pharmacological Interventions:**  - In addition to medication, consider incorporating lifestyle modifications, bladder training, and pelvic floor exercises as part of a comprehensive management plan.  - **Cognitive and Health Considerations:**  - Given the potential cognitive side effects of antimuscarinic agents, particularly in older adults, it is crucial to monitor any changes in cognitive function and discuss them with your healthcare provider.  By working closely with your healthcare provider, you can find a treatment plan that effectively manages your symptoms while minimizing side effects. |
| **^&^DeepSeek-R1:**  chat_to_reasoning>Okay, the user is a 65-year-old woman with urgency urinary incontinence. She wants to know the first-line medical therapies and their side effects. Let me start by recalling the guidelines for urgency incontinence.  >  >First-line treatments usually include behavioral interventions, but since she's asking specifically about medical therapies, I should focus on medications. The main classes are anticholinergics and beta-3 adrenergic agonists.  >  >Anticholinergics like oxybutynin, tolterodine, darifenacin, solifenacin, and fesoterodine are common. Each has different side effect profiles, but generally, they can cause dry mouth, constipation, blurred vision, and cognitive effects, especially in older adults. It's important to mention the risk of confusion or worsening cognition in the elderly.  >  >Then there's mirabegron, a beta-3 agonist. It works differently, so the side effects are less related to anticholinergic effects. It might cause increased blood pressure, headache, or urinary tract infections. Since she's 65, checking blood pressure is crucial.  >  >I should also note that anticholinergics might not be ideal for elderly patients with existing cognitive issues. Maybe mirabegron is a better first-line option here. Also, topical estrogen could be considered if vaginal atrophy is contributing, but that's more of an adjunct.  >  >She might be on other medications, so drug interactions are a concern. For example, anticholinergics interacting with other anticholinergic drugs increasing side effects. Need to mention starting with the lowest dose and monitoring.  >  >Non-pharmacological options like bladder training or pelvic floor exercises should be part of a comprehensive plan, but she asked for medical therapies. Still, it's worth a brief mention as combination therapy is often more effective.  >  >What about contraindications? For anticholinergics, narrow-angle glaucoma, gastric retention. For mirabegron, uncontrolled hypertension. Need to highlight these.  >  >Also, the importance of follow-up to assess efficacy and side effects. Maybe suggest a trial period of 4-6 weeks.  >  >Let me structure this: first list the medications, their mechanisms, common side effects, special considerations for elderly, then maybe a comparison. Also, mention starting with lifestyle modifications even though the focus is on drugs.  >  >Wait, the user specified "first-line medical therapies," so I should clarify whether behavioral interventions are considered first-line alongside meds. Guidelines often recommend starting with both. But since the question is about medical therapies, perhaps list the drugs but note that they're used in conjunction with behavioral strategies.  >  >Need to make sure the information is clear and not too technical. Avoid jargon. Use generic drug names, mention some brand names for clarity.  >  >Also, consider the patient's age: 65, so possibly other comorbidities. Polypharmacy is a concern. Emphasize the need to review all meds with a doctor.  >  >Check if there's any recent updates in guidelines. For example, some newer anticholinergics with fewer side effects or mirabegron being preferred in certain cases.  >  >Putting it all together: start with the two main drug classes, explain each, their side effects, special considerations for her age group, mention the importance of combining with behavioral approaches, and the need for monitoring and follow-up.  >chat_end_reasoning  For a 65-year-old woman with **urgency urinary incontinence (UUI)**, first-line medical therapies focus on reducing bladder overactivity and calming urinary urgency. Below is a summary of recommended medications, their mechanisms, common side effects, and key considerations for older adults:  ### **First-Line Medical Therapies**  #### **1. Anticholinergic Medications**  **Mechanism**: Block acetylcholine receptors in the bladder, reducing involuntary contractions.  **Examples**:  - **Oxybutynin** (Ditropan®): Immediate-release (IR) or extended-release (ER).  - **Tolterodine** (Detrol®).  - **Solifenacin** (Vesicare®).  - **Darifenacin** (Enablex®).  - **Fesoterodine** (Toviaz®).  **Common Side Effects**:  - Dry mouth (most frequent).  - Constipation.  - Blurred vision.  - Cognitive effects (e.g., confusion, memory issues) – especially with older agents like oxybutynin.  - Increased risk of urinary retention in men with prostate issues.  **Special Considerations for Older Adults**:  - Avoid **oxybutynin IR** due to higher risk of cognitive impairment.  - Prefer **solifenacin** or **darifenacin** (more bladder-selective, fewer central nervous system effects).  - Use the lowest effective dose to minimize side effects.  #### **2. Beta-3 Adrenergic Agonist**  **Mechanism**: Activates beta-3 receptors to relax the bladder muscle and increase storage capacity.  **Example**:  - **Mirabegron** (Myrbetriq®).  **Common Side Effects**:  - Increased blood pressure (monitor regularly).  - Headache.  - Nasopharyngitis (cold-like symptoms).  - Urinary tract infections (UTIs).  **Special Considerations**:  - Safer for older adults with cognitive concerns (no anticholinergic side effects).  - Avoid in uncontrolled hypertension.  ### **Key Guidelines for Medication Use in Older Adults**  1. **Start Low, Go Slow**: Begin with the lowest dose and titrate gradually (e.g., mirabegron 25 mg → 50 mg).  2. **Avoid Polypharmacy Risks**:  - Review existing medications to avoid interactions (e.g., anticholinergics + other anticholinergic drugs like antidepressants).  - Use the **Anticholinergic Cognitive Burden (ACB) Scale** to assess risk.  3. **Monitor for Side Effects**:  - Check blood pressure with mirabegron.  - Assess for constipation (manage with fiber, hydration, or stool softeners).  ### **Non-Drug Adjuncts to Enhance Efficacy**  - **Bladder Training**: Schedule voiding every 2–3 hours to reduce urgency.  - **Lifestyle Modifications**:  - Limit caffeine, alcohol, and artificial sweeteners.  - Treat constipation (straining worsens UUI).  - **Topical Vaginal Estrogen** (if vaginal atrophy is present): Improves urethral/bladder mucosal health.  ### **When to Reassess**  - **4–6 Weeks**: Evaluate symptom improvement and side effects.  - **3 Months**: If no improvement, switch to another drug class or consider combination therapy (e.g., mirabegron + solifenacin).  ### **Alternatives if First-Line Therapies Fail**  - **Intravesical Botox (Botulinum Toxin)**: For refractory UUI.  - **Percutaneous Tibial Nerve Stimulation (PTNS)**: Noninvasive neuromodulation.  ### **Red Flags to Report**  - Worsening confusion or hallucinations (stop anticholinergics immediately).  - Severe constipation or urinary retention.  - Systolic BP > 150 mmHg on mirabegron.  Urgency urinary incontinence is manageable with careful medication selection and monitoring. Always work with your doctor to balance efficacy, safety, and your overall health profile! |
| 1. **I am a 58-year-old woman with severe stress urinary incontinence that did not respond to conservative treatment. What are the indications for surgical management? What is the recommended preferred surgical procedure?** |
| **ChatGPT-4.0:**  For a 58-year-old woman with severe stress urinary incontinence (SUI) that has not responded to conservative treatments such as pelvic floor muscle training or behavioral modifications, surgical management may be indicated. Here’s an overview of the indications for surgical intervention and the preferred surgical procedures:  ### Indications for Surgical Management:  1. **Failure of Conservative Treatments:**  - When non-surgical methods such as pelvic floor exercises, lifestyle modifications, and possibly medications have not provided sufficient relief.  2. **Severity of Symptoms:**  - Severe and persistent symptoms that significantly impact quality of life and daily activities.  3. **Desire for Definitive Treatment:**  - A preference for a more permanent solution to manage incontinence.  4. **No Contraindications to Surgery:**  - The patient must be in suitable health for surgery, with no contraindications that would make surgery too risky.  ### Preferred Surgical Procedure:  1. **Mid-Urethral Sling Procedures:**  - **Tension-Free Vaginal Tape (TVT) and Transobturator Tape (TOT):**  - These are minimally invasive procedures where a mesh sling is placed under the mid-urethra to provide support and prevent involuntary leakage.  - TVT involves a retropubic approach, while TOT uses a transobturator approach, each with slightly different techniques and placement.  2. **Indications for Sling Procedures:**  - These procedures are generally considered the gold standard for surgical treatment of SUI due to their effectiveness and relatively quick recovery time.  - Suitable for women with uncomplicated stress urinary incontinence.  3. **Alternative Surgical Options:**  - **Burch Colposuspension:**  - An abdominal surgery that involves suturing the vaginal wall to the pelvic ligaments to provide support to the urethra.  - Less commonly performed now due to the success and minimally invasive nature of sling procedures.  - **Autologous Fascial Sling:**  - Uses the patient’s own tissue to create a sling. This is typically considered if synthetic mesh is not an option.  ### Considerations:  - **Consultation with a Specialist:**  - A thorough evaluation by a urologist or urogynecologist is essential to determine the most appropriate surgical option based on individual health, anatomy, and preferences.  - **Understanding Risks and Benefits:**  - Discuss potential risks, benefits, and the recovery process with your surgeon. Each procedure has its own set of potential complications, and understanding these is critical for informed decision-making.  - **Post-Surgical Recovery:**  - Recovery times and post-operative care instructions will vary depending on the procedure. Follow-up care is crucial to ensure proper healing and to address any complications promptly.  By working closely with your healthcare provider, you can determine the most suitable surgical option to effectively manage severe stress urinary incontinence. |
| **^&^DeepSeek-R1:**  chat_to_reasoning>Okay, let me try to figure out how to answer this question. The user is a 58-year-old woman with severe stress urinary incontinence (SUI) that hasn't gotten better with conservative treatments. She wants to know the indications for surgical management and the preferred procedure.  >  >First, I need to recall what SUI is. Stress incontinence is when you leak urine during activities like coughing, sneezing, or exercising. Conservative treatments usually include pelvic floor exercises, lifestyle changes, maybe pessaries, or medications. If those didn't work, surgery might be the next step.  >  >Indications for surgery... Well, I think it's when conservative treatments fail, the symptoms are severe enough to affect quality of life, and the patient is motivated to have surgery. Also, the patient should be a good surgical candidate, meaning no major health issues that make surgery risky.  >  >Now, for the preferred surgical procedure. I remember there are a few options. The mid-urethral sling, like the TVT (tension-free vaginal tape), used to be the gold standard. But there's been some controversy with mesh complications. Then there are other sling procedures that use the patient's own tissue, like autologous fascial sling. Also, Burch colposuspension is another option. More recently, maybe the artificial urinary sphincter for severe cases, but that's less common in women.  >  >Wait, what's recommended now? I think the mid-urethral sling is still commonly used because it's minimally invasive and effective, but there's been a shift due to mesh issues. Some guidelines now might prefer autologous slings or other procedures without mesh. The American Urological Association (AUA) or other urology societies probably have guidelines on this.  >  >I should check the latest guidelines. Let me recall. For primary SUI without prior surgery, the mid-urethral sling is considered first-line. But if there are concerns about mesh, then autologous sling or Burch might be alternatives. The patient's age and activity level might influence the choice too. For a 58-year-old, maybe the sling is still preferred.  >  >Also, patient factors: does she have other pelvic organ prolapse? If so, maybe a combined procedure. But the question says severe SUI, so maybe isolated. The preferred procedure would depend on surgeon experience, patient preference, and risk factors. But I need to state the most recommended one based on evidence.  >  >I think the mid-urethral sling is still the preferred, but with informed consent about mesh risks. Alternatively, the autologous sling is another top option. The AUA might list both as options. Also, newer procedures like adjustable slings or urethral bulking agents, but those are less effective for severe cases.  >  >So, putting it all together: indications for surgery are failure of conservative treatment, significant symptom impact, and patient suitability. The preferred procedure is mid-urethral sling (like TVT or TOT) or autologous fascial sling, depending on patient factors and surgeon expertise. Need to mention the risks, like mesh complications for TVT, and that autologous sling has higher efficacy but more invasive.  >chat_end_reasoning  For a 58-year-old woman with **severe stress urinary incontinence (SUI)** unresponsive to conservative treatments (e.g., pelvic floor muscle training, pessaries, lifestyle modifications), surgical management is the next step. Below is a concise, evidence-based summary of indications and recommended procedures:  ### **Indications for Surgical Management**  1. **Failed Conservative Therapy**: Persistent symptoms after ≥ 3 months of supervised pelvic floor muscle training ± pessary use.  2. **Severe SUI**: Frequent urine leakage during activities (e.g., coughing, sneezing, exercise) that significantly impacts quality of life.  3. **Anatomic Confirmation**: Demonstrated urethral hypermobility or intrinsic sphincter deficiency (ISD) on exam/urodynamics.  4. **Patient Readiness**: Motivated, medically fit for surgery, and willing to accept procedural risks.  ### **Recommended Preferred Surgical Procedures**  #### **1. Mid-Urethral Sling (MUS)**  - **Mechanism**: Polypropylene mesh tape placed under the mid-urethra to provide support during increased abdominal pressure.  - **Types**:  - **Retropubic (e.g., TVT – Tension-Free Vaginal Tape)**: Higher efficacy for ISD.  - **Transobturator (e.g., TOT)**: Lower risk of bladder injury but slightly less effective for severe ISD.  - **Success Rates**: 80–90% cure/improvement at 1–5 years.  - **Advantages**: Minimally invasive, short recovery, gold standard for primary SUI.  - **Risks**:  - Mesh complications (5–10%: erosion, pain, dyspareunia).  - Urinary retention (3–5%).  **Guideline Preference**:  - **First-line** for most patients with uncomplicated SUI (AUA/SUFU 2019).  - Requires **informed consent** about mesh risks (FDA warnings for transvaginal mesh).  #### **2. Autologous Fascial Pubovaginal Sling**  - **Mechanism**: Sling crafted from the patient’s own rectus fascia or fascia lata, placed at the bladder neck.  - **Success Rates**: 85–90% cure/improvement (similar to MUS).  - **Advantages**: No synthetic mesh; preferred for patients with:  - Prior mesh complications.  - ISD or recurrent SUI.  - Concerns about mesh (e.g., younger, sexually active women).  - **Risks**: Longer surgery, donor-site morbidity (pain, hernia), higher retention rates (5–10%).  **Guideline Preference**:  - **Co-first-line** with MUS for ISD or patients avoiding mesh (IUGA/ICS 2022).  ---  ### **Alternative Procedures**  - **Burch Colposuspension**: Open/laparoscopic bladder neck suspension (70–80% success). Less common today due to invasiveness.  - **Urethral Bulking Agents** (e.g., collagen, Coaptite®): Temporary improvement; reserved for frail patients unfit for surgery.  - **Artificial Urinary Sphincter**: Rarely used in women; reserved for severe ISD with prior failed slings.  ### **Key Considerations**  1. **Preoperative Workup**:  - Urodynamic testing if diagnosis is unclear or mixed incontinence is suspected.  - Assess for pelvic organ prolapse (may require concurrent repair).  2. **Shared Decision-Making**:  - Discuss mesh risks vs. efficacy (MUS) versus autologous sling’s higher invasiveness.  - Patient priorities (e.g., desire for mesh-free surgery, activity level).  ### **Red Flags to Monitor Post-Op**  - **Mesh Erosion**: Vaginal bleeding, discharge, or pain.  - **Urinary Retention**: Requiring catheterization beyond 2 weeks.  - **Recurrent SUI**: May need revision or alternative therapy.  ### **Bottom Line**  For **severe SUI refractory to conservative treatment**, **mid-urethral sling (MUS)** remains the preferred minimally invasive option, while **autologous fascial sling** is ideal for mesh-averse patients. Both have high success rates, and choice depends on patient factors and surgeon expertise. Always weigh risks/benefits through shared decision-making! |
| 1. **I am a 62-year-old woman scheduled for anti-incontinence surgery, the tension-free vaginal tape (TVT) is considered. What are the contraindications and potential complications of TVT surgery?** |
| **ChatGPT-4.0:**  Tension-free vaginal tape (TVT) surgery is a common and effective procedure for treating stress urinary incontinence. However, like any surgical procedure, it has contraindications and potential complications. Here’s an overview:  ### Contraindications for TVT Surgery:  1. **Active Urinary Tract Infection:**  - Surgery should be postponed until the infection is resolved to reduce the risk of complications.  2. **Untreated Coagulopathy or Anticoagulation Therapy:**  - Patients with bleeding disorders or those on anticoagulant medications may have an increased risk of bleeding during or after the procedure.  3. **Severe Vaginal Atrophy:**  - Significant atrophy may require pre-treatment to improve tissue quality before surgery.  4. **Previous Pelvic Radiation:**  - Radiation can affect tissue healing and increase the risk of complications.  5. **Pregnancy:**  - TVT is not performed during pregnancy.  6. **Pelvic Organ Prolapse Requiring Repair:**  - If significant prolapse is present, it may need to be addressed either before or concurrently with incontinence surgery.  7. **Severe Urethral Mobility or Intrinsic Sphincter Deficiency:**  - These conditions may require alternative surgical approaches.  ### Potential Complications of TVT Surgery:  1. **Intraoperative Complications:**  - **Bladder or Urethral Injury:**  - Can occur during needle passage and may require immediate repair.  2. **Postoperative Complications:**  - **Urinary Retention:**  - Difficulty urinating may occur and sometimes requires temporary catheterization or tape adjustment.  - **Voiding Dysfunction:**  - Some patients may experience difficulty emptying the bladder fully.  - **De Novo Urgency or Urge Incontinence:**  - New onset of urgency or urge incontinence can develop post-surgery.  - **Mesh Erosion or Exposure:**  - The mesh may erode into the vaginal wall or urethra, requiring further surgical intervention.  - **Infection:**  - Infection at the surgical site or urinary tract infection can occur.  - **Pain or Discomfort:**  - Pelvic or groin pain may occur, sometimes persisting long-term.  - **Bleeding or Hematoma:**  - Bleeding during or after surgery can lead to hematoma formation.  3. **Long-term Complications:**  - **Chronic Pain:**  - Some patients may experience chronic pelvic pain.  - **Recurrent Incontinence:**  - Over time, some patients may experience a return of incontinence symptoms.  ### Considerations:  - **Preoperative Evaluation:**  - A thorough evaluation by your surgeon will help assess your suitability for TVT and address any potential contraindications.  - **Informed Consent:**  - Understanding the risks and benefits of the procedure is crucial. Discuss any concerns with your surgeon to ensure all your questions are answered.  - **Postoperative Care:**  - Follow your surgeon’s instructions for postoperative care closely to minimize complications and promote healing.  By discussing these factors with your healthcare provider, you can make an informed decision about proceeding with TVT surgery and understand what to expect during the recovery process. |
| **^&^DeepSeek-R1:**  chat_to_reasoning>Okay, so the user is a 62-year-old woman scheduled for TVT surgery and wants to know the contraindications and potential complications. Let me start by recalling what TVT is. TVT stands for tension-free vaginal tape, a common surgical procedure for stress urinary incontinence. It's a type of mid-urethral sling using synthetic mesh.  >  >First, I need to outline the contraindications. Contraindications are conditions that make a procedure inadvisable. For TVT, absolute contraindications would include things like active infection, because surgery could worsen it. Pregnancy is another one since the procedure is for non-pregnant women. Allergies to the materials used, like polypropylene mesh, would be a no-go. Also, if someone has a urethral fistula or untreated bladder issues like cancer, those are absolute contraindications.  >  >Then there are relative contraindications. These are situations where TVT might not be the best choice but could still be considered with caution. For example, if the patient has intrinsic sphincter deficiency (ISD) without urethral hypermobility, TVT might not work as well. Mixed urinary incontinence where urgency is a big component could be tricky, as the surgery targets stress incontinence. Previous pelvic radiation could complicate healing, and conditions that impair healing like diabetes or immunosuppression are relative concerns. Recurrent UTIs would need to be managed first. Also, severe prolapse might require a different approach.  >  >Now, moving on to complications. I need to categorize them into intraoperative, early postoperative, and long-term. Intraoperative complications include bladder perforation, which can happen when inserting the trocars. Bleeding is another risk, especially from the retropubic space. There's also a risk of injury to nearby structures like the bowel or blood vessels, though rare.  >  >Early postoperative complications might include urinary retention if the tape is too tight, requiring catheterization. Infections like UTI or wound infections can occur. Hematoma formation in the retropubic area is possible. Some patients might experience urgency symptoms after surgery, which could be temporary or require treatment.  >  >Long-term complications include mesh erosion or extrusion, where the tape wears through the vaginal wall. This can cause pain, discharge, or dyspareunia. Chronic pain in the pelvis or thighs is another concern. Recurrent SUI might happen if the surgery isn't successful. Rarely, there could be obstruction leading to voiding dysfunction or even fistula formation.  >  >Special considerations for older adults: At 62, the user might have comorbidities like diabetes or weaker tissues, which could affect healing. Vaginal atrophy could increase erosion risk, so maybe topical estrogen would help. Cognitive issues might make postoperative care harder, so ensuring support is important.  >  >I should also mention the importance of informed consent, especially regarding mesh complications. The FDA has warnings about transvaginal mesh, so patients need to be aware of the risks versus benefits.  >  >Wait, did I cover all the key points? Let me check the previous answers to maintain consistency. The previous response on surgical management for SUI mentioned MUS as first-line, with mesh complications and success rates. So here, TVT as a type of MUS, needs similar structure. Make sure contraindications are absolute vs. relative, and complications categorized by timing.  >  >Also, maybe mention alternative procedures if TVT is contraindicated, like autologous sling or Burch colposuspension. But the user specifically asked about TVT's contraindications and complications, so alternatives can be a brief part of the answer.  >  >Need to present this in a clear, organized way without markdown. Use headings and bullet points in plain text. Ensure the language is accessible but precise. Highlight the most common complications versus rare ones. Emphasize the need for preoperative evaluation and patient counseling.  >chat_end_reasoning  For a 62-year-old woman considering **tension-free vaginal tape (TVT)** surgery for stress urinary incontinence (SUI), it’s critical to understand the **contraindications** and **potential complications** associated with this procedure. Below is a detailed breakdown:  ### **Contraindications to TVT Surgery**  #### **Absolute Contraindications**  1. **Active Pelvic Infection**: Increased risk of mesh infection or abscess.  2. **Pregnancy**: TVT is not intended for women planning future pregnancies.  3. **Allergy to Polypropylene Mesh**: Rare but critical to screen preoperatively.  4. **Untreated Bladder/Urethral Pathology**:  - Bladder cancer.  - Urethral diverticulum or fistula.  5. **Severe Intrinsic Sphincter Deficiency (ISD) Without Urethral Hypermobility**: TVT relies on urethral support; poor efficacy if ISD is the primary issue.  #### **Relative Contraindications**  1. **Mixed Urinary Incontinence with Dominant Urgency**: TVT targets SUI, not urgency; anticholinergics or beta-3 agonists may be needed post-op.  2. **Prior Pelvic Radiation**: Increased risk of mesh erosion due to tissue fibrosis.  3. **Chronic Immunosuppression or Poor Healing** (e.g., uncontrolled diabetes): Higher risk of infection/mesh complications.  4. **Recurrent UTIs**: Requires resolution before surgery.  5. **Severe Pelvic Organ Prolapse**: May need concurrent prolapse repair.  ### **Potential Complications of TVT Surgery**  #### **Intraoperative Risks**  1. **Bladder or Urethral Perforation** (2–5%):  - Occurs during trocar placement; repaired intraoperatively but may require prolonged catheterization.  2. **Bleeding** (1–3%):  - Retropubic hematoma (more common with retropubic TVT vs. transobturator).  3. **Bowel/Vascular Injury** (<1%): Rare but life-threatening if trocars injure iliac vessels or bowel.  #### **Early Postoperative Complications**  1. **Urinary Retention** (5–10%):  - Due to over-tightening of the tape; may require catheterization or tape loosening.  2. **Urinary Tract Infection (UTI)** (10–15%):  - Managed with antibiotics.  3. **Acute Urgency/Overactive Bladder (OAB) Symptoms** (10–20%):  - Temporary in most cases; may need medications (e.g., mirabegron).  #### **Long-Term Complications**  1. **Mesh Erosion/Extrusion** (2–5%):  - Tape erodes into vagina, urethra, or bladder, causing pain, discharge, or dyspareunia. Requires partial/full mesh removal.  2. **Chronic Pelvic or Groin Pain** (3–8%):  - Linked to nerve entrapment or inflammation; may necessitate tape excision.  3. **Recurrent SUI** (10–15% at 5 years):  - Higher risk with obesity, chronic cough, or ISD.  4. **Voiding Dysfunction** (3–5%):  - Persistent difficulty emptying the bladder.  5. **Rare Complications**:  - Fistula formation, mesh contraction (pain/tightness), or hypersensitivity to mesh.  ### **Special Considerations for Older Adults**  - **Vaginal Atrophy**: Increases erosion risk; topical estrogen may be prescribed preoperatively.  - **Frailty/Comorbidities**: Assess surgical fitness (e.g., cardiac/pulmonary status).  - **Cognitive Impairment**: Ensure ability to comply with post-op care (e.g., catheter management).  ### **Preoperative Counseling Tips**  1. **Mesh Risks**: Discuss FDA warnings about transvaginal mesh (e.g., erosion, pain).  2. **Alternatives**:  - **Autologous Fascial Sling**: No mesh, ideal for mesh-averse patients.  - **Burch Colposuspension**: Open/laparoscopic bladder neck suspension.  - **Urethral Bulking Agents**: Less effective but minimally invasive.  3. **Shared Decision-Making**: Balance efficacy (~85–90% cure rate) vs. complication risks.  ### **When to Avoid TVT**  - If **mesh allergy**, **active infection**, or **severe ISD without hypermobility** is present, opt for **autologous sling** or **bulking agents**.  TVT remains a **gold-standard** minimally invasive option for SUI, but thorough patient selection and informed consent are crucial. Always discuss personalized risks/benefits with your surgeon! |
| 1. **I am a 70-year-old woman with both stress and urge incontinence. How can treatment strategies be developed? Should one type of incontinence be addressed preferentially?** |
| **ChatGPT-4.0:**  Managing a combination of stress urinary incontinence (SUI) and urge urinary incontinence (UUI), often referred to as mixed urinary incontinence, requires a comprehensive and individualized approach. Here’s how treatment strategies can be developed:  ### Initial Evaluation:  1. **Comprehensive Assessment:**  - A thorough evaluation by a healthcare provider, typically a urologist or urogynecologist, is essential. This may include a detailed medical history, physical examination, and potentially urodynamic testing to understand the underlying causes and severity of each type of incontinence.  2. **Identification of Predominant Symptoms:**  - Determine which type of incontinence (stress or urge) is more bothersome or has a greater impact on your quality of life. This can help guide the prioritization of treatment.  ### Treatment Strategies:  1. **Lifestyle and Behavioral Modifications:**  - **Pelvic Floor Muscle Exercises (Kegel Exercises):**  - Strengthening the pelvic floor muscles can help manage both SUI and UUI.  - **Bladder Training:**  - This involves scheduled voiding and gradually increasing the time between voids to help manage urgency and frequency.  - **Dietary Modifications:**  - Reducing caffeine, alcohol, and irritant foods can help with urge incontinence.  2. **Medications:**  - **Anticholinergics or Beta-3 Agonists:**  - These medications can help reduce symptoms of UUI by relaxing the bladder muscle and reducing urgency.  - **Topical Estrogen:**  - For postmenopausal women, topical estrogen may improve urethral and vaginal tissue health, potentially aiding in the management of SUI.  3. **Prioritizing Treatment:**  - If one type of incontinence is significantly more bothersome, it may be addressed first. For example, if urgency and frequency are more disruptive, medications and bladder training might be prioritized.  4. **Surgical Options:**  - If conservative measures are not effective and SUI is the predominant issue, surgical options like mid-urethral sling procedures (e.g., TVT) may be considered.  5. **Combination Approach:**  - Often, a combination of treatments is necessary. For example, pelvic floor exercises may be combined with medications to address both stress and urge components.  6. **Regular Follow-Up:**  - Regular follow-up with your healthcare provider is important to assess the effectiveness of treatments and make adjustments as needed.  ### Decision-Making:  - **Patient Preferences:**  - Your preferences and lifestyle considerations should play a significant role in the development of a treatment plan.  - **Quality of Life:**  - Treatments should aim to improve your overall quality of life, considering both the effectiveness and potential side effects of each option.  By working closely with your healthcare provider, you can develop a tailored treatment plan that addresses both types of incontinence effectively. This collaborative approach ensures that your individual needs and preferences are considered, leading to better management of symptoms and improved quality of life. |
| **^&^DeepSeek-R1:**  chat_to_reasoning>Okay, let's tackle this. The user is a 70-year-old woman dealing with both stress and urge incontinence. She wants to know how to develop treatment strategies and whether one type should be prioritized.  >  >First, I need to recall that mixed incontinence (both stress and urge) is common, especially in older women. The challenge here is addressing both components effectively.  >  >The previous conversations were about surgical options for stress incontinence, specifically TVT sling. But now, it's mixed, so surgery alone might not be the answer. I should consider non-surgical approaches first, especially since she's older and might have comorbidities.  >  >I remember that conservative treatments like lifestyle modifications, pelvic floor exercises, and bladder training are first-line for mixed incontinence. Since she's 70, checking for factors like mobility issues, cognitive function, and medication use (like diuretics) is important. Polypharmacy could worsen symptoms, so reviewing her meds is a key step.  >  >Then, addressing both types of incontinence: stress might need pelvic floor muscle training, while urge could benefit from bladder training and anticholinergics or beta-3 agonists. But in older adults, medications have side effects like dry mouth or cognitive effects, so alternatives like mirabegron might be better.  >  >If conservative methods fail, then considering more invasive options. But with mixed incontinence, surgery for stress (like a sling) might not resolve urge symptoms and could even worsen them. So it's crucial to evaluate which component is more bothersome to her. If stress is predominant, maybe surgery after controlling urge. If urge is worse, focus on that first.  >  >Also, pelvic organ prolapse might coexist, so a physical exam is necessary. Urodynamics could help clarify the diagnosis, especially if there's uncertainty.  >  >The user might be concerned about undergoing surgery, given her age, but also wants effective treatment. The answer should balance efficacy with safety, emphasizing a stepwise approach and shared decision-making. Highlighting the importance of treating both types but tailoring based on her specific symptoms and preferences is key. Need to make sure the information is clear and not too technical, considering her possible familiarity from previous discussions.  >chat_end_reasoning  For a 70-year-old woman with **mixed urinary incontinence (MUI)**—both **stress urinary incontinence (SUI)** and **urge urinary incontinence (UUI)**—the treatment strategy requires a **balanced approach** to address both components. Here’s a stepwise, evidence-based plan:  ### **Key Principles for Managing Mixed Incontinence**  1. **Treat Both Components Simultaneously**: SUI and UUI often coexist, so therapy should target both.  2. **Prioritize the Most Bothersome Symptom**: If one type (stress vs. urge) significantly impacts quality of life, address it first.  3. **Start Conservative, Progress to Advanced**: Begin with non-invasive therapies before considering surgery.  4. **Evaluate for Comorbidities**: Rule out reversible contributors (e.g., UTIs, medications, pelvic organ prolapse).  ### **Stepwise Treatment Strategy**  #### **1. Conservative Measures (First-Line for All Patients)**  - **Lifestyle Modifications**:  - Weight loss (if overweight).  - Reduce caffeine/alcohol intake.  - Fluid management (avoid excessive fluids before bedtime).  - **Bladder Training**:  - Scheduled voiding (every 2–3 hours) to reduce urgency.  - Gradually increase intervals between bathroom trips.  - **Pelvic Floor Muscle Training (PFMT)**:  - Supervised Kegel exercises to improve urethral support (for SUI) and suppress urgency (for UUI).  - Consider referral to a pelvic floor physical therapist.  - **Topical Vaginal Estrogen** (if vaginal atrophy present):  - Improves urethral/bladder mucosal health, reducing both SUI and UUI.  #### **2. Pharmacologic Therapy for Urge Component**  - **Anticholinergics** (e.g., oxybutynin, tolterodine):  - Reduce bladder overactivity but may cause dry mouth, constipation, or cognitive side effects (caution in older adults).  - **Beta-3 Agonists** (e.g., **mirabegron**):  - Preferred in older adults due to fewer side effects; enhances bladder relaxation.  - **Combination Therapy**:  - Mirabegron + PFMT improves outcomes better than either alone.  #### **3. Advanced Therapies if Conservative Measures Fail**  - **Pessary Trial**:  - A urethral-support pessary may reduce SUI while continuing UUI therapy.  - **Neuromodulation**:  - **Percutaneous Tibial Nerve Stimulation (PTNS)** or **Sacral Neuromodulation (InterStim®)**: Effective for refractory UUI with SUI.  - **Intravesical Botox (OnabotulinumtoxinA)**:  - For severe UUI unresponsive to medications; risk of urinary retention (requires self-catheterization skills).  #### **4. Surgical Options for Stress Component**  Surgery (e.g., mid-urethral sling) may be considered **only if**:  - SUI is the **dominant symptom** and significantly impacts quality of life.  - UUI is well-controlled with conservative/pharmacologic therapy.  - **Caution**: Surgery for SUI can sometimes worsen UUI if the urge component is not adequately managed.  ### **Should One Type Be Addressed First?**  - **If SUI is Predominant** (e.g., leaks only with coughing/sneezing):  - Start with PFMT ± pessary. If insufficient, consider surgery **after** optimizing UUI treatment.  - **If UUI is Predominant** (e.g., frequent urgency, nocturia):  - Prioritize bladder training, anticholinergics/mirabegron, or neuromodulation.  - **If Both Are Equally Severe**:  - Combine PFMT + pharmacologic therapy (e.g., mirabegron) + bladder training.  ### **Special Considerations in Older Adults**  1. **Frailty/Comorbidities**:  - Avoid anticholinergics in patients with cognitive impairment (risk of delirium).  - Simplify regimens to enhance adherence (e.g., once-daily mirabegron).  2. **Mobility Issues**:  - Ensure easy bathroom access; treat constipation (straining worsens SUI).  3. **Medication Review**:  - Diuretics, sedatives, or ACE inhibitors may exacerbate symptoms.  ### **Red Flags to Evaluate**  - **Recurrent UTIs**: Treat and rule out residual urine (post-void residual volume check).  - **Pelvic Organ Prolapse**: May require concurrent treatment (e.g., pessary or surgery).  - **Hematuria or Pain**: Rule out bladder cancer or stones.  ### **Bottom Line**  For mixed incontinence:  1. **Start with conservative therapies** (PFMT, bladder training, topical estrogen).  2. **Add medications for UUI** (mirabegron preferred in older adults).  3. **Consider surgery for SUI only if urge symptoms are controlled** and SUI remains severe.  4. **Neuromodulation or Botox** are options for refractory cases.  Always involve the patient in shared decision-making, balancing risks, benefits, and personal priorities! |
| 1. **I am a 30-year-old woman and I have stress urinary incontinence at 32 weeks of gestation. What should I do? Is postpartum intervention recommended?** |
| **ChatGPT-4.0:**  Experiencing stress urinary incontinence (SUI) during pregnancy is relatively common due to the physiological and anatomical changes that occur. Here’s how you can manage SUI during pregnancy and considerations for postpartum intervention:  ### Management During Pregnancy:  1. **Pelvic Floor Exercises (Kegel Exercises):**  - Strengthening the pelvic floor muscles can help improve incontinence symptoms. Aim to perform these exercises regularly, ideally several times a day.  2. **Bladder Management:**  - Practice regular voiding schedules to avoid a full bladder, which can exacerbate leakage.  - Avoid fluids that may irritate the bladder, such as caffeine and carbonated drinks, especially if they seem to worsen your symptoms.  3. **Lifestyle Modifications:**  - Maintain a healthy weight gain during pregnancy, as excess weight can increase pressure on the bladder.  - Stay hydrated, but try to distribute fluid intake throughout the day rather than consuming large amounts at once.  4. **Supportive Garments:**  - Consider wearing absorbent pads or liners designed for incontinence to manage any leakage discreetly and comfortably.  ### Postpartum Considerations:  1. **Initial Recovery:**  - After childbirth, give your body some time to recover naturally. Many women experience improvement in SUI as their body heals postpartum.  2. **Continued Pelvic Floor Exercises:**  - Continue with pelvic floor exercises after delivery. These exercises can help restore muscle tone and function.  3. **Postpartum Check-Up:**  - Discuss your symptoms with your healthcare provider during your postpartum check-up. They can assess your condition and suggest further interventions if necessary.  4. **Further Interventions:**  - If SUI persists beyond the initial postpartum period, your healthcare provider might recommend further evaluation and treatment, such as physical therapy or other interventions.  5. **Surgical Options:**  - Surgery is rarely considered immediately postpartum. It is typically reserved for cases where conservative measures have not been effective and is usually considered after you have completed childbearing.  ### When to Seek Help:  - If your incontinence significantly impacts your quality of life or does not improve postpartum, seek advice from your healthcare provider. They can refer you to a specialist, such as a urogynecologist or pelvic floor physical therapist, for further evaluation and management.  By taking proactive steps during pregnancy and postpartum, you can effectively manage SUI and improve your overall comfort and quality of life. |
| **^&^DeepSeek-R1:**  chat_to_reasoning>Okay, the user is a 30-year-old woman at 32 weeks pregnant experiencing stress urinary incontinence (SUI). She wants to know what to do now and if she needs intervention after delivery.  >  >First, I need to address her current situation. Pregnancy often causes SUI due to increased pressure on the bladder and pelvic floor. At 32 weeks, the priority is safe, conservative management. She should avoid anything that could harm the pregnancy. Pelvic floor exercises (Kegels) are the go-to. I should explain how to do them properly because incorrect technique is common. Maybe suggest consulting a pelvic floor physiotherapist for guidance.  >  >Lifestyle adjustments are important too. Weight management, avoiding heavy lifting, reducing caffeine, and fluid intake timing. She should also be aware of positions that reduce bladder pressure, like leaning forward when urinating. Maybe mention using pads for leakage but avoid long-term use of tampons or constrictive clothing.  >  >Now, postpartum considerations. SUI often improves after delivery, but not always. She should continue Kegels postpartum. Wait 6-12 weeks post-delivery to see if symptoms persist. If they do, then consider further interventions. It's important to note that immediate postpartum intervention isn't usually recommended unless symptoms are severe or there's another complication.  >  >Potential red flags: If she has sudden pain, bleeding, or signs of infection, she should seek immediate help. Also, postpartum, if symptoms worsen or there's urinary retention, she needs evaluation.  >  >I should list the steps clearly: immediate actions during pregnancy, postpartum monitoring, and when to seek help. Avoid suggesting medications or surgery during pregnancy. Emphasize that most cases resolve postpartum but if not, there are safe options later. Make sure the tone is reassuring since SUI in pregnancy is common and manageable.  >chat_end_reasoning  For a 30-year-old woman experiencing **stress urinary incontinence (SUI)** at **32 weeks of gestation**, the approach focuses on **conservative, pregnancy-safe management** followed by **postpartum reassessment**. Here’s a structured plan:  ### **Immediate Management During Pregnancy**  1. **Pelvic Floor Muscle Training (Kegel Exercises)**  - **First-line therapy**: Strengthening pelvic floor muscles improves urethral support and reduces leakage.  - **Technique**: Contract pelvic muscles (as if stopping urine flow) for 3–5 seconds, relax for 5–10 seconds; repeat 10–15 times, 3x/day.  - **Consider referral to a pelvic floor physiotherapist** for guided training (optimal technique is key).  2. **Lifestyle and Behavioral Modifications**  - **Weight management**: Avoid excessive weight gain (increases intra-abdominal pressure).  - **Bladder habits**:  - Timed voiding (every 2–3 hours) to prevent overdistension.  - Double voiding (lean forward after urinating to empty bladder fully).  - **Avoid triggers**: Reduce caffeine, acidic foods, and heavy lifting.  3. **Pregnancy-Specific Adjustments**  - **Supportive garments**: Maternity support belts can reduce pelvic pressure.  - **Postural strategies**: Avoid prolonged standing; use a small footstool when sitting to reduce pelvic floor strain.  4. **Absorbent Products**  - Use **pads or period underwear** for leakage (avoid tampons, which increase infection risk).  ### **What to Avoid During Pregnancy**  - **Medications**: Anticholinergics (e.g., oxybutynin) are **not recommended** unless urgency is severe and risks are justified (rarely used in pregnancy).  - **Invasive procedures**: Surgery (e.g., slings) or pessaries are **contraindicated** during pregnancy.  ### **Postpartum Intervention: When and What?**  #### **Postpartum Reassessment**  - **Wait 6–12 weeks after delivery**: Hormonal changes and pelvic floor recovery often improve SUI spontaneously.  - **Re-evaluate symptoms**: If SUI persists or worsens after 3 months postpartum, proceed with further management.  #### **Postpartum Treatment Options**  1. **Pelvic Floor Rehabilitation**  - Resume/advance Kegel exercises; consider **biofeedback** or **electrical stimulation** for weak muscles.  - Pelvic floor physiotherapy reduces SUI in 50–70% of cases.  2. **Vaginal Estrogen** (if breastfeeding or postmenopausal)  - Topical estrogen cream improves urethral mucosal health if atrophy is present.  3. **Pessary Trial**  - A **urethral support pessary** (e.g., ring pessary) can reduce leakage during high-impact activities.  4. **Surgical Options** (if conservative measures fail)  - **Mid-urethral sling (TVT/TOT)**: Gold-standard for persistent SUI, but **delay until family planning is complete** (future pregnancies can compromise sling efficacy).  - **Autologous fascial sling**: Alternative if mesh avoidance is preferred.  ### **Key Considerations**  - **Vaginal Delivery vs. Cesarean Section**:  - Vaginal delivery (especially operative) increases SUI risk, but C-section is **not recommended solely for SUI prevention**.  - Focus on pelvic floor recovery regardless of delivery mode.  - **Breastfeeding**:  - Low estrogen levels during breastfeeding may worsen SUI; topical vaginal estrogen is safe if needed.  ### **Red Flags to Address Immediately**  - **Urinary retention** (inability to void postpartum).  - **Signs of infection** (fever, dysuria, foul-smelling urine).  - **Sudden onset incontinence** (rule out obstetric fistula if trauma occurred during delivery).  ### **Prognosis**  - **Up to 50% of SUI cases resolve spontaneously by 6 months postpartum**.  - Persistent SUI is more likely with:  - Pre-pregnancy SUI.  - High birth weight (>4 kg).  - Prolonged second stage of labor.  ### **Bottom Line**  - **During pregnancy**: Focus on Kegels, lifestyle adjustments, and coping strategies.  - **Postpartum**: Reassess at 6–12 weeks; escalate treatment only if symptoms persist.  - **Surgery is delayed** until after childbearing is complete.  SUI in pregnancy is common and often temporary—reassurance and proactive pelvic floor care are key! |
| 1. **I am a 50-year-old woman with a BMI of 36 and moderate stress incontinence. The effect of pelvic floor muscle training is limited. What is the impact of obesity on treatment options?** |
| **ChatGPT-4.0:**  Obesity can have a significant impact on stress urinary incontinence (SUI) and the effectiveness of its treatment. Here are some considerations and strategies for managing SUI with a higher BMI:  ### Impact of Obesity on SUI:  1. **Increased Intra-Abdominal Pressure:**  - Excess weight can increase pressure on the bladder and pelvic floor, exacerbating symptoms of SUI.  2. **Reduced Effectiveness of Pelvic Floor Exercises:**  - While pelvic floor muscle training (Kegel exercises) is beneficial, its effectiveness may be limited in individuals with obesity due to additional strain on the pelvic muscles.  3. **Higher Risk of Comorbidities:**  - Obesity is often associated with other health conditions, such as diabetes and hypertension, which can complicate the management of incontinence.  ### Treatment Considerations and Options:  1. **Weight Management:**  - **Weight Loss:**  - Even modest weight loss can significantly reduce the severity of SUI symptoms. A structured weight loss program involving dietary changes and regular physical activity can be beneficial.  - **Nutritional Counseling:**  - Working with a dietitian can help create a sustainable and balanced diet plan tailored to your needs.  2. **Enhanced Pelvic Floor Training:**  - **Supervised Physical Therapy:**  - Consider seeing a pelvic floor physical therapist who can provide personalized guidance and advanced techniques to strengthen the pelvic muscles.  - **Biofeedback:**  - This technique can help you learn how to better control and strengthen your pelvic floor muscles.  3. **Behavioral and Lifestyle Modifications:**  - **Bladder Training:**  - Implementing a scheduled voiding regimen can help manage symptoms.  - **Avoid Bladder Irritants:**  - Reduce intake of caffeine, alcohol, and other bladder irritants.  4. **Medical and Surgical Options:**  - **Medications:**  - While medications are more commonly used for urge incontinence, your doctor might explore options if they believe it could help your symptoms.  - **Surgical Intervention:**  - If conservative measures are insufficient, surgical options such as a mid-urethral sling procedure may be considered. However, the success of surgery can be influenced by obesity, and weight loss is often recommended before pursuing surgical options.  5. **Regular Monitoring and Support:**  - Regular follow-up with your healthcare provider is crucial to monitor progress, adjust treatment plans, and address any emerging health concerns related to obesity.  ### Conclusion:  Addressing obesity can play a crucial role in the management of SUI. By focusing on weight management and working closely with healthcare providers, you can enhance the effectiveness of existing treatments and potentially reduce the severity of your symptoms. |
| **^&^DeepSeek-R1:**  chat_to_reasoning>Okay, let's tackle this question. The user is a 50-year-old woman with a BMI of 36 and moderate stress incontinence. Pelvic floor muscle training (PFMT) hasn't worked well for her. She wants to know how obesity affects her treatment options.  >  >First, I need to recall how obesity impacts stress urinary incontinence (SUI). Excess weight increases intra-abdominal pressure, which puts more strain on the pelvic floor muscles. That can weaken them over time, making SUI worse. So, higher BMI is a known risk factor for SUI and can make treatments less effective.  >  >Since PFMT hasn't been effective, maybe her weight is a contributing factor. Studies show that even a 5-10% weight loss can improve SUI symptoms. So, weight management should be a key part of her treatment plan. But how to approach that? Lifestyle changes, dietary modifications, exercise, maybe even bariatric surgery if appropriate. However, exercise might be challenging due to SUI, so low-impact activities like swimming or cycling could be suggested.  >  >Next, other non-surgical options. Pessaries might be an option. A urethral support pessary could help, but in obese patients, fitting might be tricky. Maybe a ring pessary with support? Also, vaginal estrogen could help if there's atrophy, improving mucosal health and possibly enhancing PFMT effectiveness.  >  >Pharmacological treatments for SUI are limited. Duloxetine is an SNRI used off-label, but side effects like nausea are common. Might not be the best first-line, but could be considered.  >  >If conservative measures fail, surgical options. Mid-urethral slings are the gold standard, but obesity increases surgical risks like infection, wound complications, and sling failure. Studies show lower cure rates in obese patients. However, some evidence suggests that with proper technique, slings can still be effective. Autologous fascial slings are another option, maybe more durable in obese patients but with longer recovery.  >  >Combining weight loss with other treatments might be the way to go. For example, starting with weight loss interventions alongside a pessary or PFMT. If she loses weight, surgical outcomes might improve. Also, addressing comorbidities like diabetes or hypertension, which are common with obesity, could be important.  >  >Need to mention that even modest weight loss helps. Maybe refer her to a dietitian or a weight management program. Also, consider multidisciplinary care involving a urogynecologist, physiotherapist, and nutritionist.  >  >Possible red flags: If she has severe prolapse or other pelvic floor issues, that might complicate things. Also, if there's urge incontinence mixed in, treatment approach might differ, but the question specifies stress incontinence.  >  >In summary, obesity complicates SUI treatment by reducing the effectiveness of PFMT, increasing surgical risks, and necessitating a focus on weight management. A stepwise approach combining weight loss, pessary use, possibly duloxetine, and then surgery if needed, with careful consideration of risks vs benefits.  >chat_end_reasoning  For a 50-year-old woman with a **BMI of 36** and **moderate stress urinary incontinence (SUI)** that has responded poorly to pelvic floor muscle training (PFMT), obesity significantly impacts treatment options. Here’s a tailored, evidence-based approach:  ### **Impact of Obesity on SUI and Treatment**  1. **Mechanisms**:  - Excess weight increases intra-abdominal pressure, straining the pelvic floor and urethral closure mechanism.  - Adipose tissue produces inflammatory cytokines, weakening connective tissue and exacerbating SUI.  - Obesity reduces PFMT efficacy due to chronic pelvic floor overload.  2. **Prognosis**:  - Higher BMI correlates with **lower cure rates** for both conservative and surgical SUI treatments.  - Even modest weight loss (5–10% of body weight) improves SUI severity by 40–70%.  ### **Stepwise Treatment Strategy**  #### **1. Prioritize Weight Loss**  - **Lifestyle Modifications**:  - **Dietary changes**: Reduce caloric intake with a focus on protein and fiber to preserve muscle mass.  - **Low-impact exercise**: Swimming, cycling, or chair yoga to avoid worsening SUI during high-impact activities.  - **Medical Weight Loss**:  - **GLP-1 agonists** (e.g., semaglutide) or **orlistat** (if appropriate) to support sustained weight loss.  - **Bariatric Surgery**:  - Consider if BMI ≥ 40 (or ≥ 35 with obesity-related comorbidities). SUI improvement is often seen post-surgery.  #### **2. Optimize PFMT**  - **Intensify Training**:  - Add **biofeedback** or **electrical stimulation** to improve pelvic floor muscle activation.  - Ensure proper technique with a **pelvic floor physiotherapist** (up to 50% of women perform Kegels incorrectly).  - **Combination Therapy**:  - Pair PFMT with **vaginal estrogen** (if atrophic changes are present) to enhance urethral mucosal health.  #### **3. Mechanical Support**  - **Pessary**:  - A **urethral-support pessary** (e.g., ring pessary with knob) can reduce leakage. Regular cleaning and sizing by a clinician are critical.  - Obesity may make self-insertion/removal challenging, but properly fitted pessaries are effective in 60–80% of cases.  #### **4. Pharmacotherapy**  - **Duloxetine** (SNRI):  - Off-label use for SUI; reduces leakage by enhancing urethral sphincter tone.  - **Side effects**: Nausea (20–30%), fatigue; start at 20 mg BID and titrate.  #### **5. Surgical Options**  - **Mid-Urethral Sling (MUS)**:  - Gold-standard surgery, but obesity increases risks of:  - **Sling failure** (10–15% higher risk vs. non-obese patients).  - **Wound complications** (e.g., infection, mesh exposure).  - **Outcomes**: Cure rates drop from ~85% (non-obese) to 70–75% (BMI > 30).  - **Prefer retropubic (TVT) over transobturator (TOT) approach** in obese patients (better long-term efficacy).  - **Autologous Fascial Sling**:  - Alternative for patients avoiding synthetic mesh; higher durability in obese women but requires longer recovery.  - **Perioperative Care**:  - Optimize glycemic control (if diabetic) and screen for sleep apnea.  ### **Key Considerations**  - **Comorbidities**: Address diabetes, hypertension, or chronic cough (e.g., from obesity hypoventilation), which worsen SUI.  - **Avoid Monotherapy**: Combine weight loss + PFMT + pessary for synergistic effects.  - **Shared Decision-Making**: Discuss realistic expectations—obesity lowers cure rates but does not preclude success.  ### **Prognosis with Weight Loss**  - A 5–10% weight loss (e.g., 9–18 lbs for a 180-lb woman) can:  - Reduce SUI episodes by 30–50%.  - Improve surgical outcomes if pursued later.  ### **Bottom Line**  1. **Weight loss is foundational**—refer to a weight management specialist.  2. **Intensify PFMT** with biofeedback/electrical stimulation.  3. **Pessaries or duloxetine** for interim symptom control.  4. **Surgery is viable but requires careful patient selection and counseling** about higher risks.  Obesity complicates SUI management but does not eliminate effective treatment—multimodal strategies yield the best outcomes. |
| 1. **I am a 65-year-old woman who underwent a TVT 3 months ago, with marked improvement in my symptoms. How often and what should I schedule my follow-up visits? What are the potential long-term complications?** |
| **ChatGPT-4.0:**  It's great to hear that you've experienced marked improvement following your tension-free vaginal tape (TVT) procedure. Follow-up care is an important aspect of ensuring long-term success and addressing any complications should they arise. Here's a general guideline for follow-up visits and potential long-term considerations:  ### Follow-Up Schedule:  1. **Initial Postoperative Follow-Up:**  - Typically, your first follow-up visit is scheduled around 6-8 weeks post-surgery. Since you are 3 months post-op, you should have already had this visit. If not, it’s advisable to schedule one soon.  2. **Subsequent Visits:**  - After the initial postoperative visit, follow-up visits are usually scheduled at 6 months and then annually, unless issues arise that require more frequent monitoring.  3. **As Needed:**  - If you experience any new symptoms or have concerns, schedule an appointment with your healthcare provider outside of the regular schedule.  ### Potential Long-Term Complications:  While TVT is generally considered safe and effective, there are potential complications that can occur in the long term:  1. **Urinary Tract Infections (UTIs):**  - Some women may experience recurrent UTIs after the procedure.  2. **Voiding Dysfunction:**  - Difficulty with urination, such as a slow stream or incomplete emptying, can occur. This might require further evaluation and management.  3. **Tape Erosion or Exposure:**  - Rarely, the tape can erode into the vagina or urethra, which may necessitate surgical intervention.  4. **Chronic Pain:**  - Some women may experience pelvic or groin pain, which should be evaluated by a healthcare provider.  5. **Recurrence of Incontinence:**  - Although the procedure is highly effective, there is a possibility of recurrence of stress urinary incontinence over time.  6. **Sexual Dysfunction:**  - Some women report changes in sexual function post-surgery, which should be discussed with a healthcare provider if it occurs.  ### Monitoring and Management:  - **Regular Assessments:**  - During follow-up visits, your healthcare provider will assess your urinary function, check for any signs of complications, and address any concerns you may have.  - **Prompt Reporting:**  - Report any symptoms such as pain, difficulty urinating, or signs of infection promptly to your healthcare provider.  - **Lifestyle and Pelvic Health:**  - Continue with pelvic floor exercises if recommended, maintain a healthy weight, and manage any other health conditions that may impact your urinary health.  By adhering to a regular follow-up schedule and being proactive about any new symptoms, you can help ensure the long-term success of your TVT procedure. Always feel free to reach out to your healthcare provider with any questions or concerns you may have. |
| **^&^DeepSeek-R1:**  chat_to_reasoning>Okay, let's see. The user is a 65-year-old woman who had a TVT procedure three months ago and has seen significant improvement. She wants to know about follow-up schedules and long-term complications.  >  >First, I need to recall what TVT is. TVT stands for tension-free vaginal tape, a common surgical treatment for stress urinary incontinence. It's a type of mid-urethral sling. The procedure involves placing a synthetic mesh tape under the urethra to support it and prevent leakage when there's stress like coughing or sneezing.  >  >For follow-up visits, standard post-surgical follow-up protocols for TVT usually involve an initial check-up within the first few weeks to monitor healing and address any immediate post-op issues. Since she's already three months out, the immediate post-op period is over. But she might still need periodic check-ups. I should check guidelines. Typically, follow-up might be at 6 weeks, 6 months, 1 year, and then annually or as needed. However, since she's had marked improvement, maybe less frequent after the first year. But it's important to monitor for any late complications.  >  >Long-term complications of TVT can include mesh erosion (where the mesh protrudes into the vagina), urinary retention, recurrent incontinence, pain (especially during intercourse), urinary tract infections, and rarely, tape erosion into the bladder or urethra. There's also the possibility of tape migration over time. Additionally, there's the risk of developing overactive bladder symptoms post-surgery, though her symptoms improved, so that might not be an issue.  >  >She might also need to be aware of signs that require immediate attention, like severe pain, signs of infection (fever, discharge), difficulty urinating, or recurrent incontinence. But since the question is about scheduled follow-ups, the focus is on routine checks rather than emergency signs.  >  >I should structure the answer with recommended follow-up schedule and then list potential long-term complications with brief explanations. Maybe also include lifestyle advice or things to watch for between visits. Also, consider her age; at 65, other health factors might influence follow-up frequency, but unless specified, stick to general TVT guidelines.  >  >Wait, some sources mention that after the initial follow-ups, annual check-ups with a urogynecologist or urologist are advisable to monitor for any late complications. Also, pelvic exams might be part of these visits to check for mesh erosion. It's important to mention that even if she's symptom-free, regular check-ups help catch issues early.  >  >I should also note that while TVT has high success rates, complications can arise years later, so long-term monitoring is key. However, many patients don't need frequent visits if asymptomatic. But given her age and the fact that she's three months post-op, the next follow-up might be at 6 months, then annually unless issues arise.  >  >Potential long-term complications to list: mesh erosion/exposure, urinary retention (though usually early), de novo overactive bladder, recurrent SUI, pelvic pain, dyspareunia (painful intercourse), tape extrusion into bladder/urethra, and rare cases of chronic infection or inflammation related to the mesh.  >  >Need to present this clearly, maybe in bullet points, and advise her on when to seek immediate care versus routine follow-up. Also, reassure that most patients do well long-term, but awareness of possible complications is important.  >chat_end_reasoning  For a 65-year-old woman who underwent a **tension-free vaginal tape (TVT)** procedure 3 months ago with marked symptom improvement, here’s a structured plan for follow-up and long-term considerations:  ### **Follow-Up Schedule**  1. **Short-Term (Within 6 Months Post-Op)**  - **6-week visit**: Assess initial healing, confirm symptom resolution, and screen for early complications (e.g., urinary retention, infection).  - **3–6 month visit**: Evaluate long-term symptom control and rule out delayed complications (e.g., mesh exposure).  2. **Long-Term (Annual Monitoring)**  - **Annual visits**: Even if asymptomatic, schedule yearly check-ups with a **urogynecologist or urologist** to:  - Perform a pelvic exam (screen for mesh erosion or tape migration).  - Assess for recurrent stress incontinence (SUI) or new overactive bladder (OAB) symptoms.  - **Pelvic floor health**: Consider periodic pelvic floor physical therapy to maintain muscle strength and prevent recurrence.  3. **As-Needed Visits**  - Promptly schedule appointments if new symptoms arise (e.g., pain, recurrent leakage, dyspareunia).  ### **Potential Long-Term Complications**  While TVT has a **~85–90% long-term success rate**, be aware of these risks:  1. **Mesh Erosion/Exposure** (1–5% of cases):  - Mesh protruding into the vagina, causing pain, discharge, or bleeding.  - Managed with topical estrogen or surgical revision if symptomatic.  2. **Recurrent SUI** (5–10% over 10 years):  - Due to age-related tissue changes, weight gain, or tape loosening.  - Options: Reinforce with pelvic floor therapy, pessary, or repeat surgery.  3. **De Novo Overactive Bladder (OAB)** (5–15%):  - New urgency/frequency; treated with behavioral therapy or medications (e.g., anticholinergics, mirabegron).  4. **Chronic Pelvic Pain or Dyspareunia** (1–3%):  - May require tape removal, pain management, or physical therapy.  5. **Tape Migration/Erosion into Bladder/Urethra** (<1%):  - Causes recurrent UTIs, hematuria, or obstruction; requires cystoscopy and surgical removal.  6. **Urinary Retention** (rare after 3 months):  - Sudden inability to void warrants immediate evaluation.  ### **When to Seek Immediate Care**  - Severe pelvic/abdominal pain.  - Fever, foul discharge, or signs of infection.  - Inability to empty the bladder.  - Blood in urine or recurrent UTIs.  ### **Lifestyle and Prevention**  - **Weight management**: Maintain a healthy BMI to reduce intra-abdominal pressure on the pelvic floor.  - **Avoid heavy lifting**: Straining increases SUI recurrence risk.  - **Stay hydrated**: Prevent constipation (chronic straining weakens pelvic muscles).  ### **Key Takeaway**  Most patients experience **durable symptom relief** after TVT. While long-term complications are rare, annual monitoring ensures early detection and management. Continue celebrating your progress—success rates are high, and proactive care minimizes risks! |
| 1. **I am a 59-year-old woman with dysuria after TVT. How can I tell whether this is temporary urinary retention or a long-term complication of the procedure? What should be done?** |
| **ChatGPT-4.0:**  Difficult urination) after a tension-free vaginal tape (TVT) procedure can be concerning, and it's important to determine the underlying cause. Dysuria can be a symptom of temporary urinary retention, a urinary tract infection (UTI), or a longer-term complication related to the procedure. Here's how you can differentiate between these possibilities and the steps you should take:  ### Possible Causes of Dysuria After TVT:  1. **Temporary Urinary Retention:**  - **Symptoms:** Difficulty starting urination, a weak urinary stream, incomplete emptying, or a feeling of fullness in the bladder.  - **Resolution:** This is often temporary and may resolve as swelling decreases and tissues heal after the procedure.  2. **Urinary Tract Infection (UTI):**  - **Symptoms:** Dysuria, increased frequency and urgency of urination, cloudy or strong-smelling urine, possible fever.  - **Diagnosis:** A urinalysis and urine culture can confirm the presence of an infection.  3. **Long-Term Complications:**  - **Voiding Dysfunction:** Persistent difficulty with urination, which may require further evaluation and intervention.  - **Tape-Related Issues:** Tape erosion or improper placement could cause irritation or obstruction.  ### Steps to Take:  1. **Consult Your Healthcare Provider:**  - Schedule an appointment with your healthcare provider to discuss your symptoms. They can perform a physical examination and order tests to determine the cause of your dysuria.  2. **Diagnostic Tests:**  - **Urinalysis and Urine Culture:** To rule out or confirm a UTI.  - **Post-Void Residual (PVR) Test:** Measures the amount of urine left in the bladder after urination to assess for urinary retention.  - **Imaging or Cystoscopy:** In some cases, imaging or a direct visual examination of the bladder and urethra may be necessary.  3. **Treatment Options:**  - **For UTI:** Antibiotics will be prescribed if an infection is present.  - **For Urinary Retention:** Your provider may recommend techniques to help with urination, such as double voiding or using a catheter temporarily.  - **For Long-Term Complications:** If the tape is causing obstruction or irritation, surgical revision or removal may be considered.  4. **Monitoring and Follow-Up:**  - Keep track of your symptoms and any changes. Regular follow-up with your healthcare provider will ensure that any complications are addressed promptly.  5. **Lifestyle and Self-Care:**  - Stay hydrated, practice good hygiene, and avoid bladder irritants like caffeine and alcohol to support urinary health.  By working closely with your healthcare provider, you can determine the cause of your dysuria and find an appropriate treatment plan to address your symptoms. |
| **^&^DeepSeek-R1:**  chat_to_reasoning>Okay, let's tackle this. The user is a 59-year-old woman experiencing dysuria after a TVT procedure. She wants to know if it's temporary urinary retention or a long-term complication and what to do next.  >  >First, I need to recall what TVT involves. TVT, or tension-free vaginal tape, is a common surgery for stress urinary incontinence. Common complications include urinary retention, infection, mesh erosion, and bladder injury. Dysuria could be due to several factors here.  >  >Temporary urinary retention usually happens in the immediate post-op period. But since she's having dysuria, which is pain or burning during urination, it's important to differentiate between retention and other issues like UTI, mesh erosion, or obstruction.  >  >Urinary retention symptoms include difficulty starting urination, weak stream, feeling incomplete emptying, and possibly lower abdominal discomfort. Dysuria alone might point more towards infection, like a UTI, which is common after surgery. But if she's also having retention symptoms, that's a clue.  >  >Long-term complications could be mesh erosion into the urethra or bladder, causing irritation. That might present with dysuria, recurrent UTIs, hematuria, or pelvic pain. Another possibility is obstruction from the tape being too tight, leading to chronic retention and dysuria from straining.  >  >She needs to assess other symptoms. Fever, urgency, frequency, cloudy urine suggest UTI. Inability to void, abdominal distension suggest retention. Persistent pain, especially during intercourse, might indicate mesh erosion.  >  >The next steps should be seeking medical evaluation. A urinalysis and culture can check for infection. A post-void residual measurement via ultrasound can assess retention. If retention is confirmed, catheterization might be needed. Cystoscopy could visualize any mesh issues. Imaging like ultrasound or CT might help if obstruction is suspected.  >  >If it's a UTI, antibiotics would help. Temporary retention might require catheterization for a few days. If it's a long-term issue like mesh erosion or obstruction, surgical intervention might be necessary.  >  >I should structure the answer to guide her through symptom assessment, recommend immediate steps like seeing a doctor, outline possible tests, and explain potential treatments based on the cause. Also, emphasize not to delay evaluation because some complications can worsen if untreated.  >chat_end_reasoning  Dysuria (painful urination) after a tension-free vaginal tape (TVT) procedure can arise from multiple causes, ranging from temporary post-op issues to long-term complications. Here’s how to differentiate and manage this:  ### **Step 1: Assess Key Symptoms**  **Temporary Urinary Retention** (common in early post-op period):  - Difficulty initiating urination or weak stream.  - Sensation of incomplete bladder emptying.  - Lower abdominal discomfort/bloating.  - May resolve within days to weeks as swelling subsides.  **Long-Term Complications** (requires investigation):  1. **Urinary Tract Infection (UTI)**:  - Burning, urgency, frequency, cloudy urine, or fever.  - Occurs in ~10–20% of post-TVT patients.  2. **Mesh Erosion/Exposure**:  - Vaginal pain, discharge, bleeding, or dyspareunia (pain during sex).  - Rare (<5%) but can occur months to years later.  3. **Bladder Outlet Obstruction** (tape too tight):  - Chronic retention, straining to void, recurrent UTIs.  4. **Mesh-Related Inflammation/Irritation**:  - Persistent dysuria without infection.  ### **Step 2: Immediate Actions**  1. **Check for Red Flags**:  - Inability to void (seek emergency care).  - Fever, flank pain, or hematuria (suggests pyelonephritis or obstruction).  2. **Urinalysis and Culture**:  - Rule out UTI (most common cause of dysuria).  3. **Post-Void Residual (PVR) Measurement**:  - Use a bladder scan or catheter to check for retention.  - **Normal PVR**: <100 mL. **Retention**: >200 mL.  ### **Step 3: Diagnostic Workup**  1. **If UTI is confirmed**:  - Treat with antibiotics (e.g., nitrofurantoin or trimethoprim-sulfamethoxazole).  2. **If Retention is present**:  - **Temporary**: Short-term catheterization + alpha-blockers (e.g., tamsulosin) to relax bladder outlet.  - **Persistent**: Refer to a urogynecologist/urologist for urodynamics or cystoscopy.  3. **Cystoscopy**:  - Gold standard to assess for mesh erosion into the bladder/urethra or obstruction.  4. **Pelvic Exam**:  - Check for vaginal mesh exposure.  ### **Step 4: Management Based on Cause**  1. **Temporary Retention**:  - Usually resolves within 4–6 weeks with timed voiding, pelvic floor relaxation techniques, or medications.  2. **Bladder Outlet Obstruction**:  - **Tape Loosening**: Rarely needed but may require surgical tape adjustment.  3. **Mesh Erosion**:  - Partial or full tape excision if erosion is confirmed.  4. **Chronic Inflammation**:  - Topical estrogen (if vaginal atrophy) or anti-inflammatory agents.  ### **When to Seek Emergency Care**  - Complete inability to void.  - Fever >100.4°F (38°C), shaking chills, or flank pain.  - Severe hematuria or worsening abdominal pain.  ### **Prognosis**  - Most early dysuria/retention resolves with conservative care.  - Long-term complications (e.g., mesh erosion) are rare but require prompt treatment to prevent damage to nearby organs.  ### **Bottom Line**  1. **Rule out UTI first** (simple urinalysis).  2. **Measure post-void residual** to assess retention.  3. **Persistent symptoms?** Demand a cystoscopy to evaluate for mesh complications.  Don’t delay evaluation—early intervention prevents long-term harm. Schedule an appointment with your surgeon or urogynecologist ASAP. |
| 1. **I am a 70-year-old woman presenting with symptoms of stress urinary incontinence 2 years after TVT. How should I assess possible causes of recurrence? What are the recommended management strategies?** |
| **ChatGPT-4.0:**  Experiencing a recurrence of stress urinary incontinence (SUI) two years after undergoing a tension-free vaginal tape (TVT) procedure can be frustrating. It's important to assess the possible causes of recurrence and explore management strategies to address the issue. Here's a structured approach to evaluating and managing recurrent SUI:  ### Assessment of Possible Causes:  1. **Clinical Evaluation:**  - **Medical History:** Review any changes in your health status, medications, or lifestyle that might contribute to incontinence.  - **Physical Examination:** A pelvic exam can help assess any anatomical changes, such as pelvic organ prolapse, that may contribute to SUI.  2. **Diagnostic Tests:**  - **Urinalysis:** To rule out a urinary tract infection, which can exacerbate incontinence symptoms.  - **Urodynamic Testing:** This can provide detailed information about bladder and urethral function and help determine if there are any underlying issues affecting continence.  - **Imaging:** Ultrasound or other imaging modalities may be used to assess the position of the tape and any anatomical changes.  3. **Assessment of Tape Function:**  - Consider whether the tape has loosened or shifted, which might contribute to the recurrence of symptoms.  ### Recommended Management Strategies:  1. **Conservative Management:**  - **Pelvic Floor Muscle Training (PFMT):** Engage in regular pelvic floor exercises (Kegel exercises) to strengthen the muscles that support the bladder and urethra.  - **Lifestyle Modifications:** Maintain a healthy weight, manage fluid intake, and avoid bladder irritants like caffeine and alcohol.  2. **Behavioral Therapies:**  - **Bladder Training:** Techniques to increase the interval between voiding can help reduce the frequency of incontinence episodes.  3. **Pharmacological Treatment:**  - While there are limited medications specifically for SUI, addressing any concurrent overactive bladder symptoms with medication might be beneficial.  4. **Surgical Options:**  - **Tape Revision or Replacement:** If the tape has loosened or shifted, a surgical revision may be considered.  - **Alternative Surgical Procedures:** Other surgical options, such as autologous fascial sling or bulking agents, may be explored if the TVT is no longer effective.  5. **Follow-Up Care:**  - Regular follow-up with your healthcare provider is important to monitor your symptoms and make any necessary adjustments to your treatment plan.  By conducting a thorough assessment and working closely with your healthcare provider, you can identify the underlying causes of recurrent SUI and explore effective management strategies tailored to your needs. |
| **^&^DeepSeek-R1:**  chat_to_reasoning>Okay, the user is a 70-year-old woman experiencing stress urinary incontinence (SUI) symptoms two years after a TVT procedure. She wants to know how to assess the possible causes of recurrence and the recommended management strategies. Let me start by recalling what TVT is. TVT stands for tension-free vaginal tape, a common surgical treatment for SUI. It's usually effective, but recurrence can happen.  >  >First, I need to think about why SUI might come back after a successful TVT. Possible causes include age-related factors like tissue atrophy or weakening of pelvic muscles, weight gain, chronic coughing (maybe from smoking or COPD), hormonal changes post-menopause leading to vaginal atrophy, or even a failure of the tape itself like loosening or breaking. There's also the possibility of new-onset detrusor overactivity or another type of incontinence mixed in, which might be confused with SUI.  >  >The user is 70, so age-related factors are significant. Atrophy due to decreased estrogen is common in postmenopausal women. She might not have used local estrogen therapy, which could contribute to tissue weakness. Also, pelvic floor muscles can weaken over time, especially without regular exercises. If she had any chronic conditions like COPD or a chronic cough, that increases intra-abdominal pressure and could stress the tape.  >  >She mentioned the recurrence is after two years, so it's not an immediate post-op issue. I should consider both patient-related factors and tape-related factors. Patient factors include weight gain, new medical conditions, or lifestyle changes. Tape-related factors include erosion, migration, or mechanical failure.  >  >Assessment should start with a detailed history: when did the symptoms start, any associated symptoms like urgency or frequency, presence of cough or sneeze incontinence, any changes in weight, chronic cough, or other medical issues. A physical exam would check for pelvic organ prolapse, vaginal atrophy, cough stress test, and the position/integrity of the tape. Tests like urinalysis to rule out UTI, post-void residual measurement, urodynamic studies to confirm SUI and rule out other types of incontinence, and possibly imaging or cystoscopy if tape complications are suspected.  >  >Management strategies depend on the cause. If it's due to pelvic floor weakness, pelvic floor physiotherapy might help. If there's vaginal atrophy, topical estrogen could be beneficial. Weight loss advice if applicable. If the tape has failed, options include repeat surgery (maybe a different sling or a colposuspension), or less invasive options like urethral bulking agents. For patients not wanting surgery, pessaries might be an option. Also, addressing chronic cough or other contributing factors is important.  >  >I should make sure to mention red flags like signs of tape erosion (pain, discharge, dyspareunia) which would need prompt evaluation. Also, consider referral to a specialist like a urogynecologist or urologist for further assessment, especially if initial measures don't work.  >  >Wait, did the previous interactions mention anything about her current symptoms? In the history, she had a TVT 2 years ago with improvement, now SUI is back. So no mention of urgency or other symptoms. Need to differentiate pure SUI from mixed incontinence. Urodynamics can help with that. Also, check if the cough stress test is positive now. If the tape is intact but loose, maybe a repeat sling. If the tape is eroded or causing obstruction, that's a different scenario.  >  >In the answer, I should structure the assessment steps first, then management. Make sure to cover both non-surgical and surgical options, and emphasize the importance of a thorough evaluation to determine the exact cause. Also, mention lifestyle modifications and possible referrals.  >chat_end_reasoning  For a 70-year-old woman with recurrent stress urinary incontinence (SUI) 2 years after a tension-free vaginal tape (TVT) procedure, the following approach is recommended to assess causes and guide management:  ### **Assessment of Recurrence**  1. **Detailed History**:  - Timing/pattern of symptoms (e.g., leakage with coughing/sneezing vs. urgency).  - Weight changes, new chronic cough (e.g., COPD), constipation, or mobility issues.  - Menopausal vaginal atrophy symptoms (dryness, dyspareunia).  - Red flags: pelvic pain, vaginal discharge, or dyspareunia (suggestive of tape erosion).  2. **Physical Exam**:  - **Cough stress test** (confirm SUI recurrence).  - Pelvic exam for:  - Pelvic organ prolapse.  - Vaginal atrophy (pale, thin mucosa).  - Tape position or erosion (palpate for tape in vaginal wall).  3. **Diagnostic Testing**:  - **Urinalysis and culture**: Rule out UTI.  - **Post-void residual (PVR)**: Assess incomplete emptying.  - **Urodynamic studies**: Confirm recurrent SUI and exclude detrusor overactivity or voiding dysfunction.  - **Cystoscopy**: If tape erosion/migration is suspected (e.g., hematuria, recurrent UTIs).  ### **Common Causes of Recurrence**  1. **Patient-Related Factors**:  - Age-related tissue atrophy (low estrogen).  - Weight gain or chronic cough (increased intra-abdominal pressure).  - Pelvic floor muscle weakness.  2. **Tape-Related Factors**:  - Tape loosening (mechanical failure).  - Tape erosion/migration into bladder/urethra (rare).  ### **Management Strategies**  #### **Non-Surgical Options**  1. **Lifestyle Modifications**:  - Weight loss (if BMI >30).  - Smoking cessation (if applicable).  - Treat chronic cough/constipation.  2. **Pelvic Floor Muscle Training**:  - Referral to a pelvic floor physical therapist for strengthening.  3. **Topical Estrogen Therapy**:  - For vaginal atrophy (e.g., vaginal estradiol cream).  4. **Pessary**:  - Mechanical support for urethra (useful if surgery is declined).  5. **Urethral Bulking Agents**:  - Injectable agents (e.g., hyaluronic acid) to improve urethral coaptation.  #### **Surgical Options**  1. **Repeat Midurethral Sling**:  - Consider a different approach (e.g., retropubic TVT if prior was transobturator).  2. **Autologous Fascial Sling**:  - Preferred in cases of prior mesh complications.  3. **Tape Adjustment/Removal**:  - If erosion/migration is confirmed (requires cystoscopy and specialist referral).  4. **Colposuspension**:  - Burch procedure for refractory cases.  ### **When to Refer to a Specialist**  - Suspected tape erosion, obstruction, or complex pelvic floor dysfunction.  - Failed conservative management.  - Need for advanced diagnostics (e.g., urodynamics, cystoscopy).  ### **Key Takeaway**  Recurrence of SUI after TVT is often multifactorial. A structured evaluation identifies treatable causes (e.g., atrophy, weight gain) or tape failure. Conservative measures or repeat surgery can restore continence in most cases. Prioritize pelvic floor health and address modifiable risk factors to optimize outcomes. |

^&^DeepSeek-R1 analysis and reasoning processes are presented in gray.
