# Supplementary material for: Battle of the artificial intelligence: a comprehensive comparative analysis of DeepSeek and ChatGPT for urinary incontinence-related questions
Source: Front Public Health. 2025 Jul 23;13:1605908. doi: 10.3389/fpubh.2025.1605908 (PMC12325333; doi:10.3389/fpubh.2025.1605908)
Supplement: Supplementary file 1 [file Data_Sheet_1.zip › Supplementary materials/Supplementary Table 2-6.docx]

**Supplementary Table 2.** Contents of mDISCERN criteria

| **mDISCERN criteria** | **5-Point Score** | | | | |
| --- | --- | --- | --- | --- | --- |
|  | **NO Partially YES** | | | | |
|  | **1** | **2** | **3** | **4** | **5** |
| 1. Are the aims clear? |  |  |  |  |  |
| 2. Does it achieve its aims? |  |  |  |  |  |
| 3. Is it relevant? |  |  |  |  |  |
| 4. Is it clear what sources of information were used to compile the publication (other than the author or producer)? |  |  |  |  |  |
| 5. Is it clear when the information used or reported in the publication was produced? |  |  |  |  |  |
| 6. Is it balanced and unbiased? |  |  |  |  |  |
| 7. Does it provide details of additional sources of support and information? |  |  |  |  |  |
| 8. Does it refer to areas of certainty? |  |  |  |  |  |

mDISCERN: modified DISCERN

**Supplementary Table 3.** Contents of GQS criteria

| **GQS criteria** | **Score** |
| --- | --- |
| Poor quality, poor flow of the site, most information missing, not at all useful for patients | 1 |
| Generally poor quality and poor flow, some information listed but many important topics missing, of very limited use to patients | 2 |
| Moderate quality, suboptimal flow, some important information is adequately discussed but others poorly discussed, somewhat useful for patients | 3 |
| Good quality and generally good flow, most of the relevant information is listed, but some topics not covered, useful for patients | 4 |
| Excellent quality and excellent flow, very useful for patients | 5 |

GQS: global quality score

**Supplementary Table 4.** Formulas of readability indexes

| **Readability indexes** | **Formula** |
| --- | --- |
| Flesch reading ease score (FRE) | 206.835 − 1.015 × (NW/NS) − 84.6 × (NSY/NW) |
| Simple measure of Gobbledygook (SMOG) | 1.043 × √ (CW ×30/NS) + 3.1291 |

NW: number of words; NS: number of sentences; NSY: number of syllables; CW: complex words (≥3 syllables)

**Supplementary Table 5.** The inter-rater reliability analysis results for the mDISCERN scores

|  | Evaluator 1^*^ | Evaluator 2^*^ | Evaluator 3^*^ | *Kappa* (95%*CI*) | *P*-value |
| --- | --- | --- | --- | --- | --- |
| ChatGPT-4.0 | 28.24 ± 1.01 | 28.20 ± 0.96 | 28.28 ± 0.89 | 0.773 (0.614, 0.883) | ＜0.001 |
| DeepSeek | 28.68 ± 1.41 | 28.76 ± 1.59 | 28.84 ± 1.89 | 0.863 (0.755, 0.932) | ＜0.001 |

^*^Mean ± standard deviation

mDISCERN: modified DISCERN; 95%CI: 95% confidence interval

**Supplementary Table 6.** The inter-rater reliability analysis results for the GQS scores

|  | Evaluator 1^*^ | Evaluator 2^*^ | Evaluator 3^*^ | *Kappa* (95%*CI*) | *P*-value |
| --- | --- | --- | --- | --- | --- |
| ChatGPT-4.0 | 4.32 ± 0.69 | 4.40 ± 0.65 | 4.24 ± 0.93 | 0.726 (0.545, 0.856) | ＜0.001 |
| DeepSeek | 4.72 ± 0.68 | 4.84 ± 0.37 | 4.72 ± 0.61 | 0.759 (0.594, 0.876) | ＜0.001 |

^*^Mean ± standard deviation

GQS: global quality score; 95%CI: 95% confidence interval
